# Supplementary material for: Phosphorylation of the DNA damage repair factor 53BP1 by ATM kinase controls neurodevelopmental programs in cortical brain organoids
Source: PLoS Biol. 2024 Sep 3;22(9):e3002760. doi: 10.1371/journal.pbio.3002760 (PMC11398655; doi:10.1371/journal.pbio.3002760)
Supplement: S1 Raw Images — (PDF) [file pbio.3002760.s002.pdf]

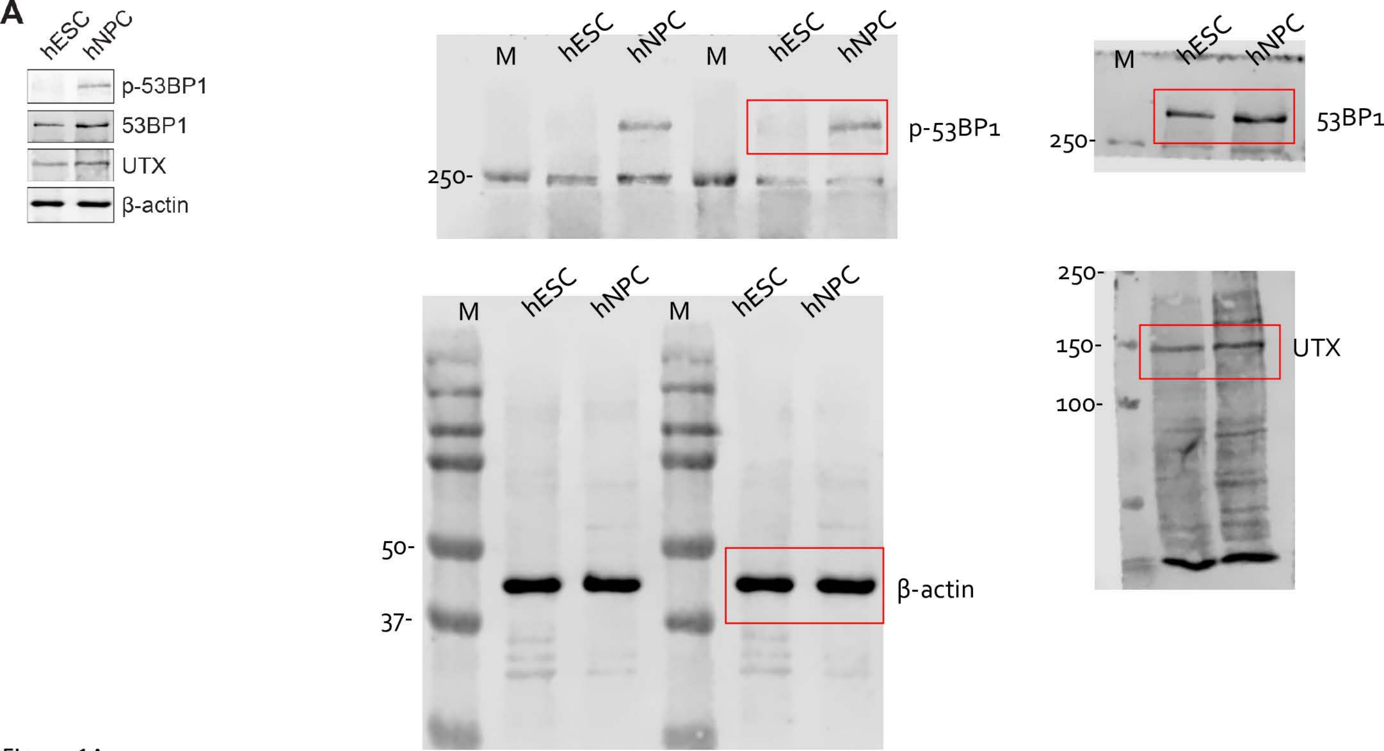

Figure 1A

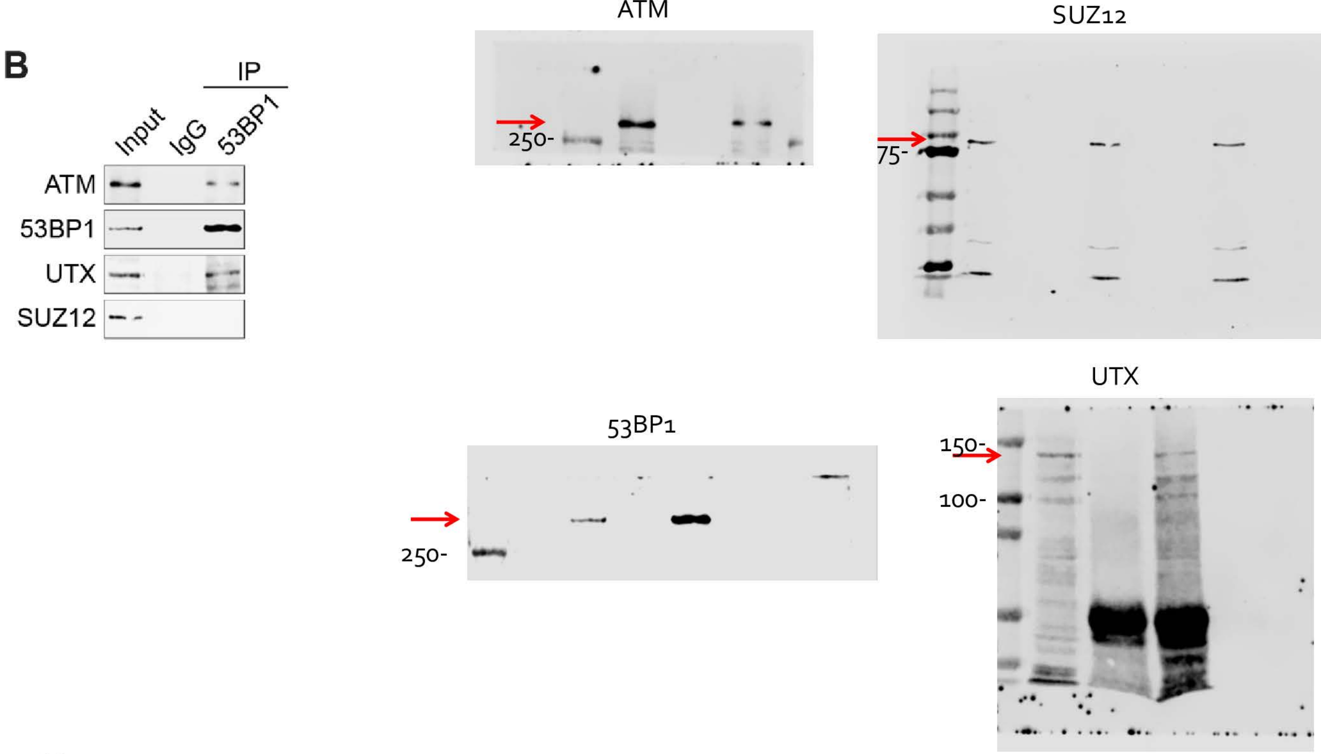

B
IP
Input
IgG
53BP1
ATM
53BP1
UTX
SUZ12
ATM
250-
SUZ12
75-
53BP1
250-
UTX
150-
100-

Figure 1B

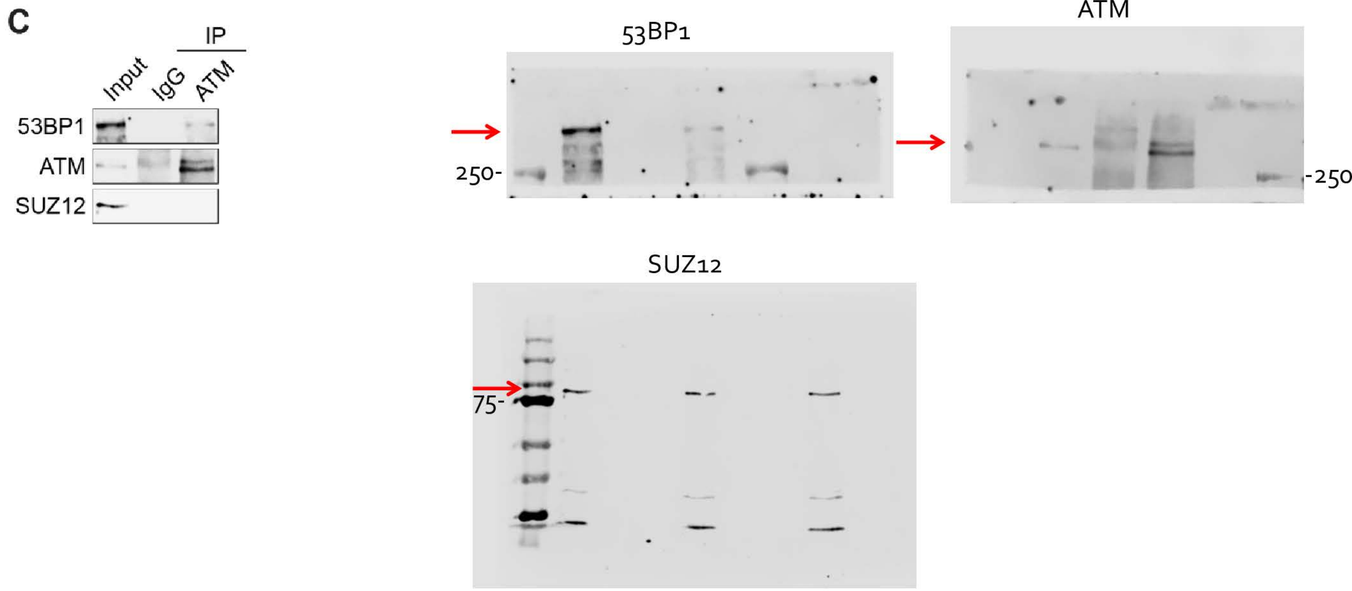

Figure 1C

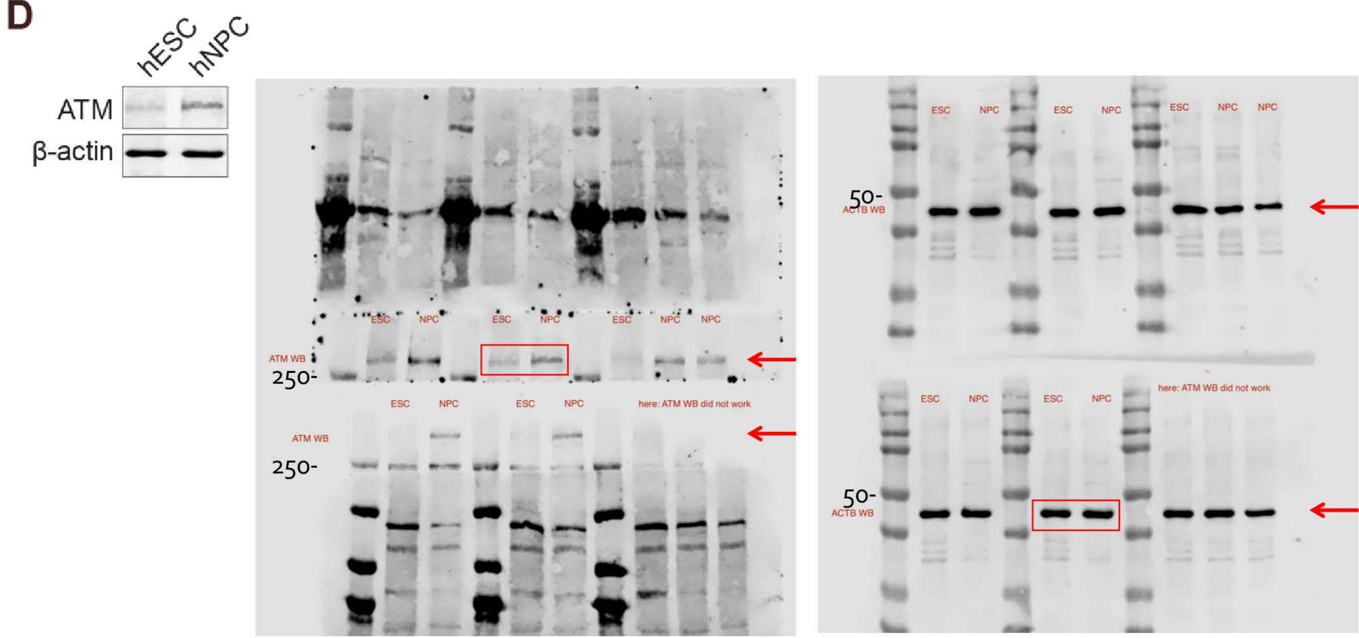
D
hESC
hNPC
ATM
β-actin
ESC
NPC
ESC
NPC
ESC
NPC
NPC
ATM WB
250-
ESC
NPC
ESC
NPC
here: ATM WB did not work
ATM WB
250-
ESC
NPC
ESC
NPC
ESC
NPC
NPC
50-
ACTB WB
ESC
NPC
ESC
NPC
here: ATM WB did not work
50-
ACTB WB

Figure 1D

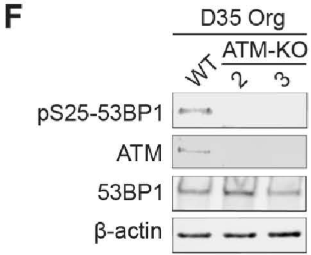
F
D35 Org
WT
ATM-KO
2
3
pS25-53BP1
ATM
53BP1
β-actin

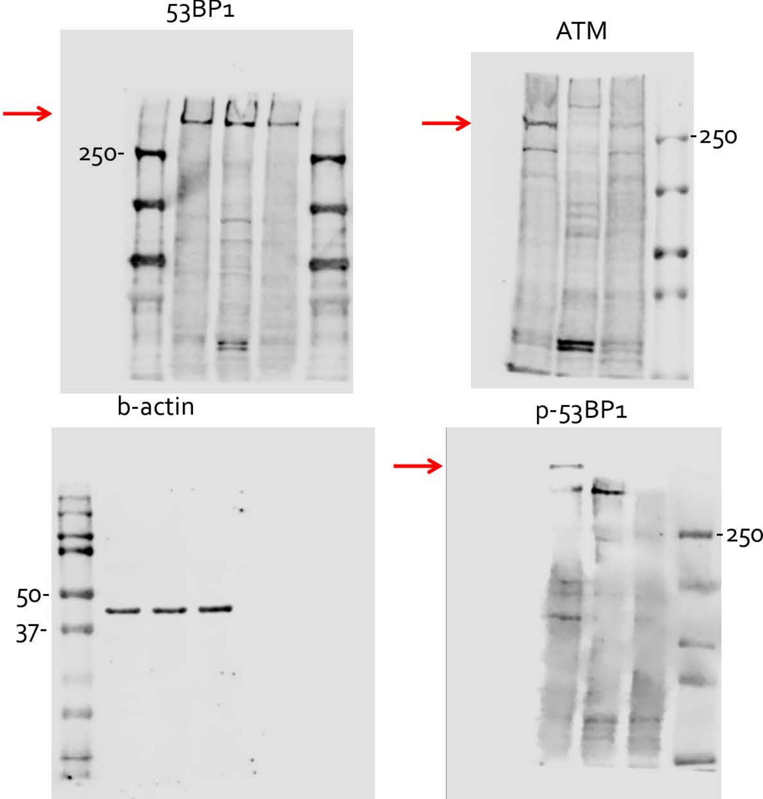
53BP1
250-
ATM
-250
b-actin
50-
37-
p-53BP1
-250

Figure 1F

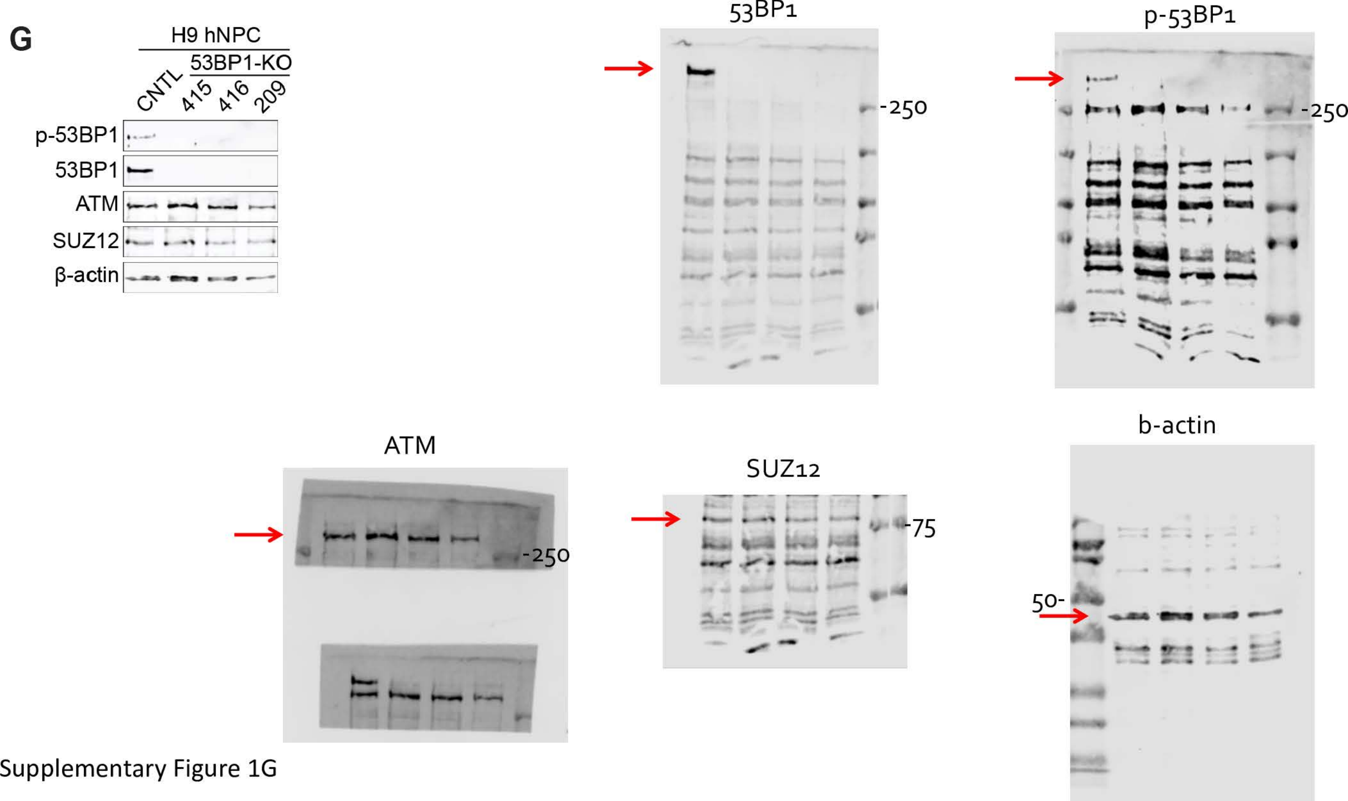

Supplementary Figure 1G

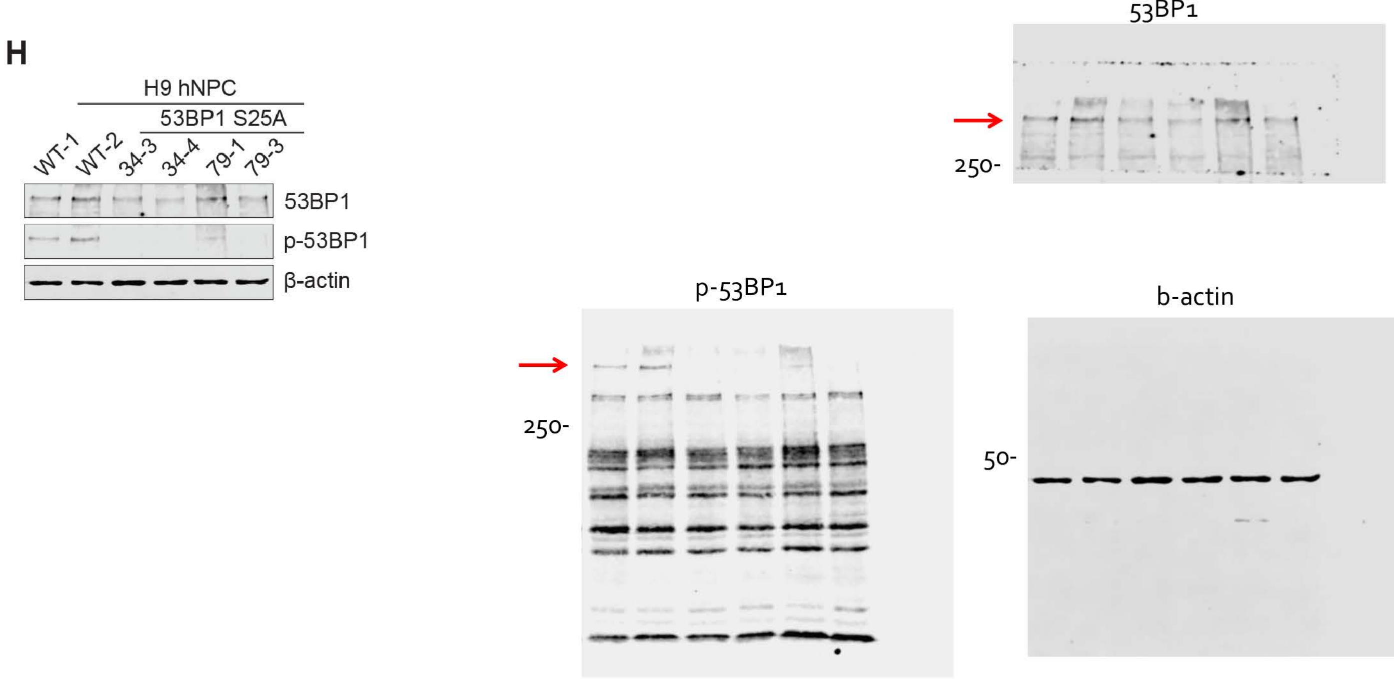

Supplementary Figure 1H

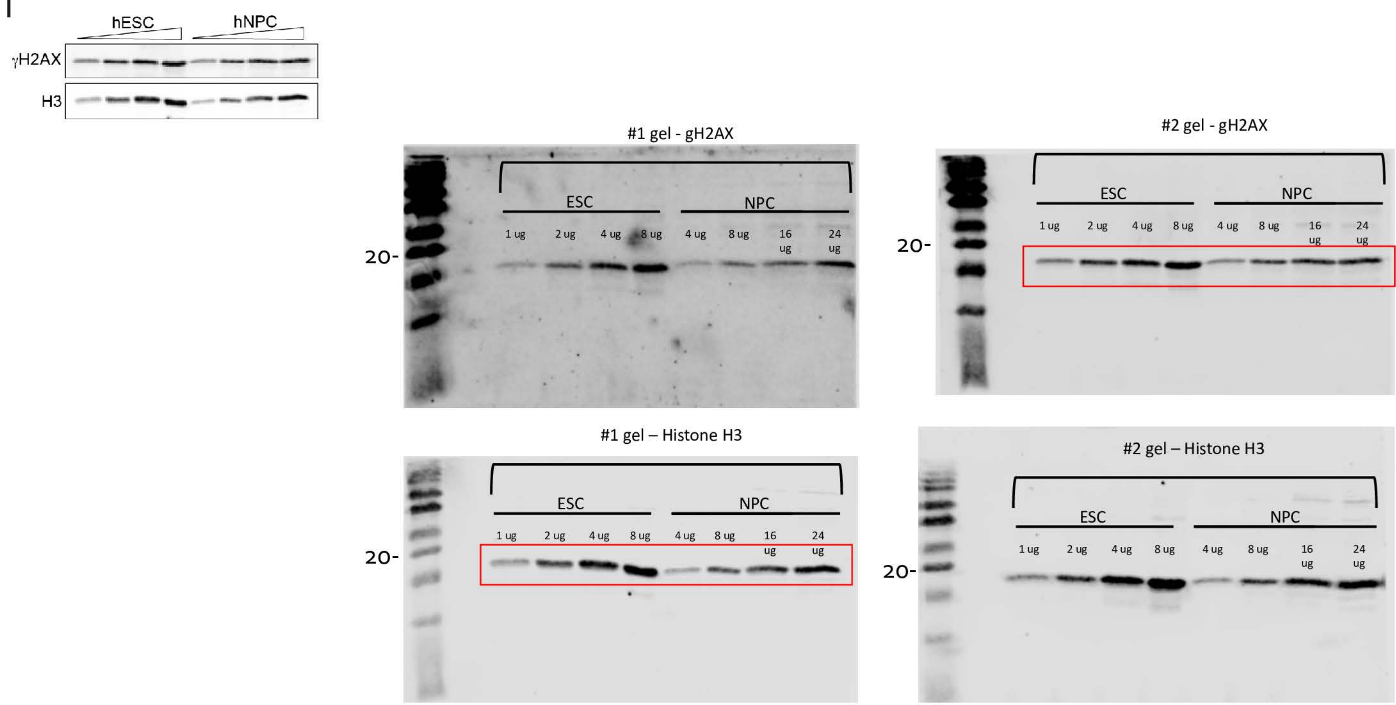

Supplementary Figure 1I

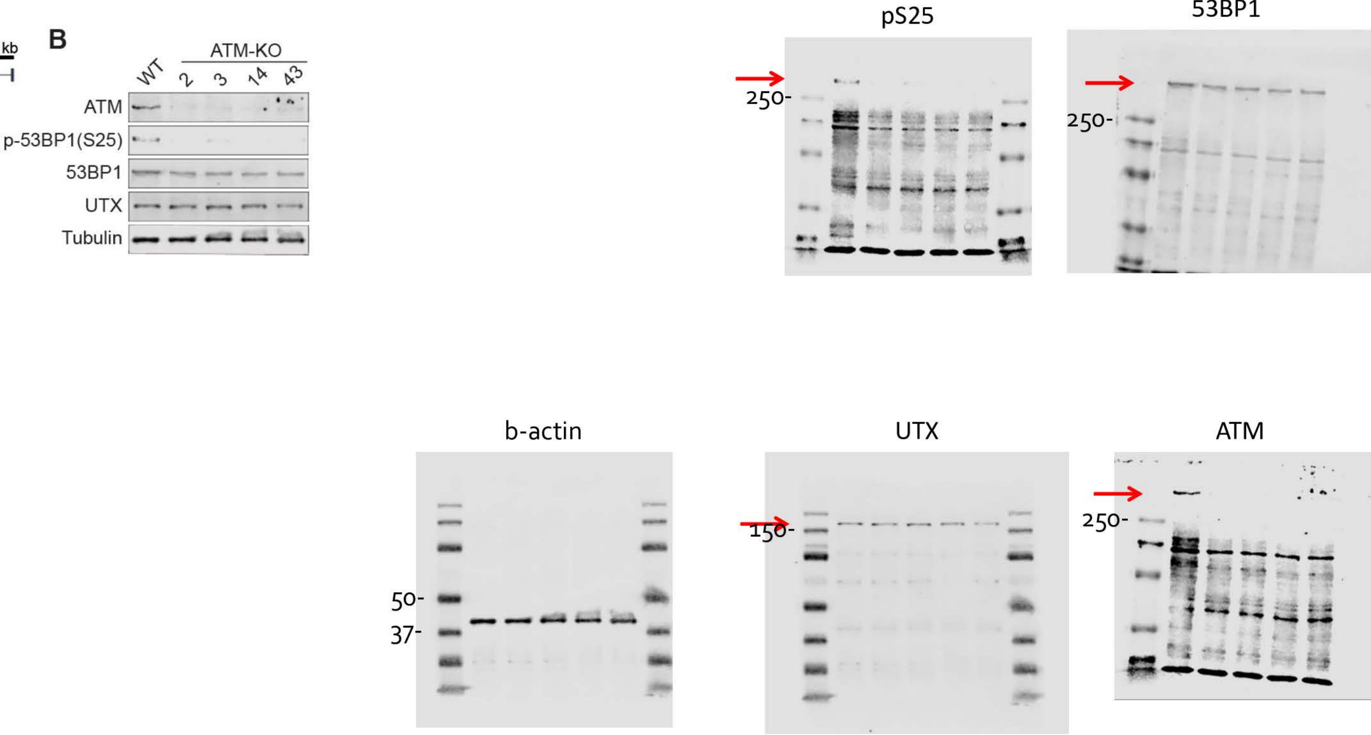

Supplementary Figure 2B

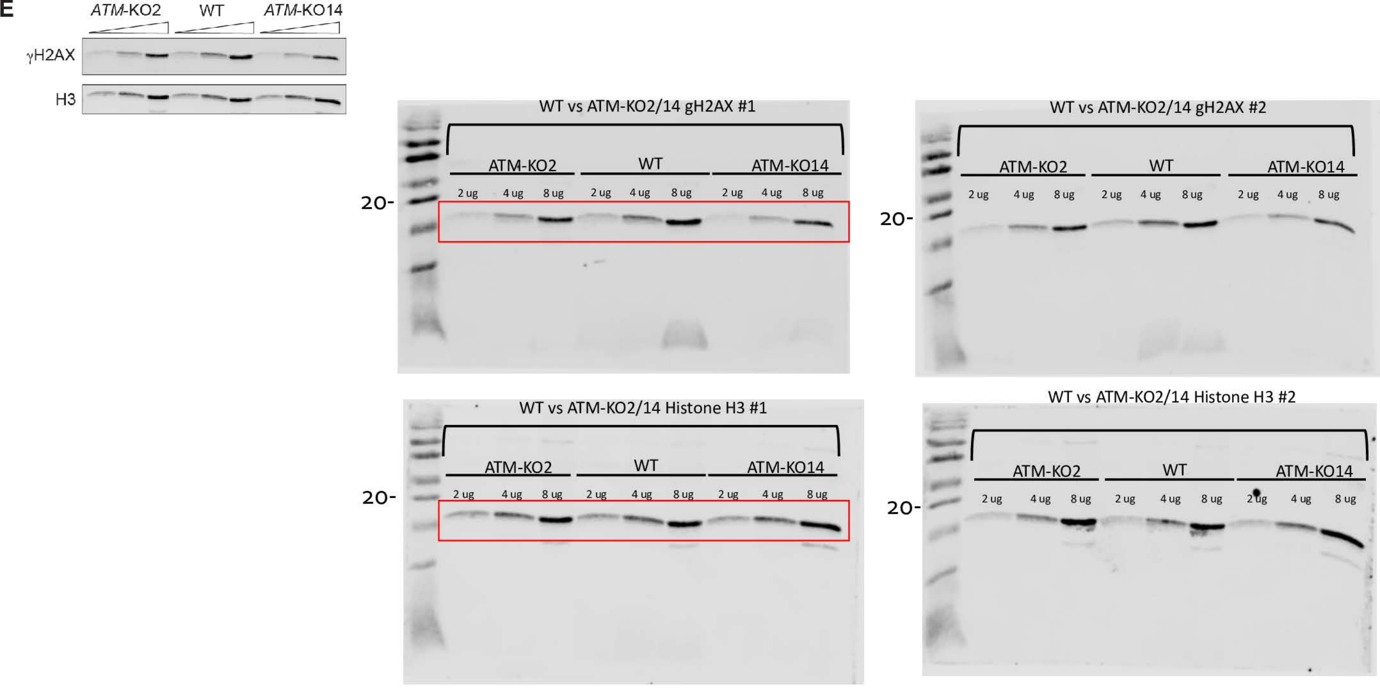

Supplementary Figure 2E

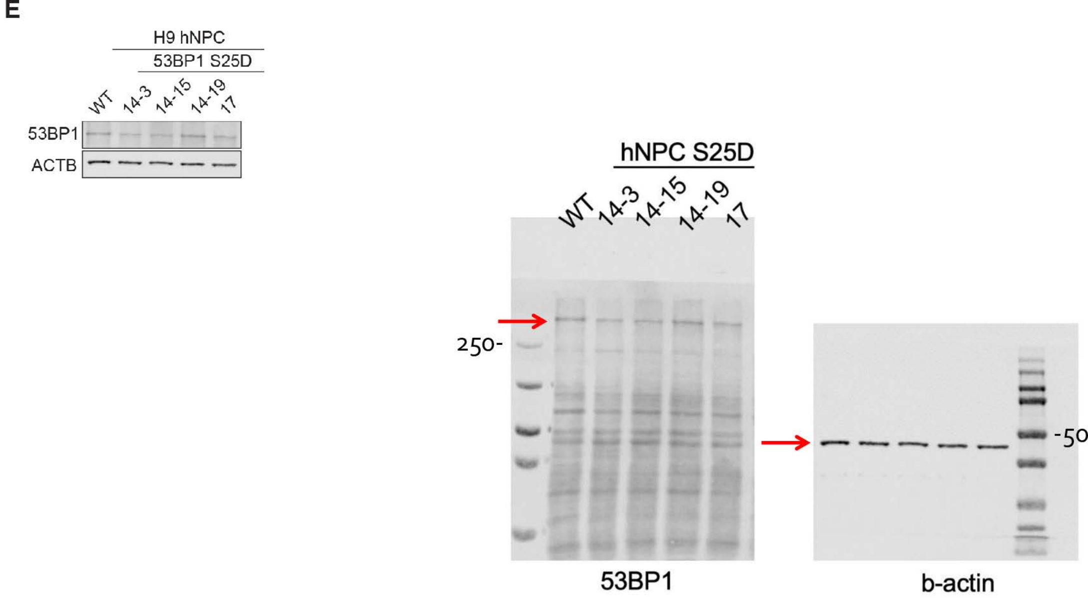

Supplementary Figure 7E

B

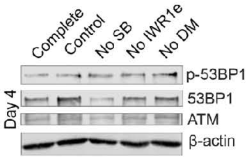

C

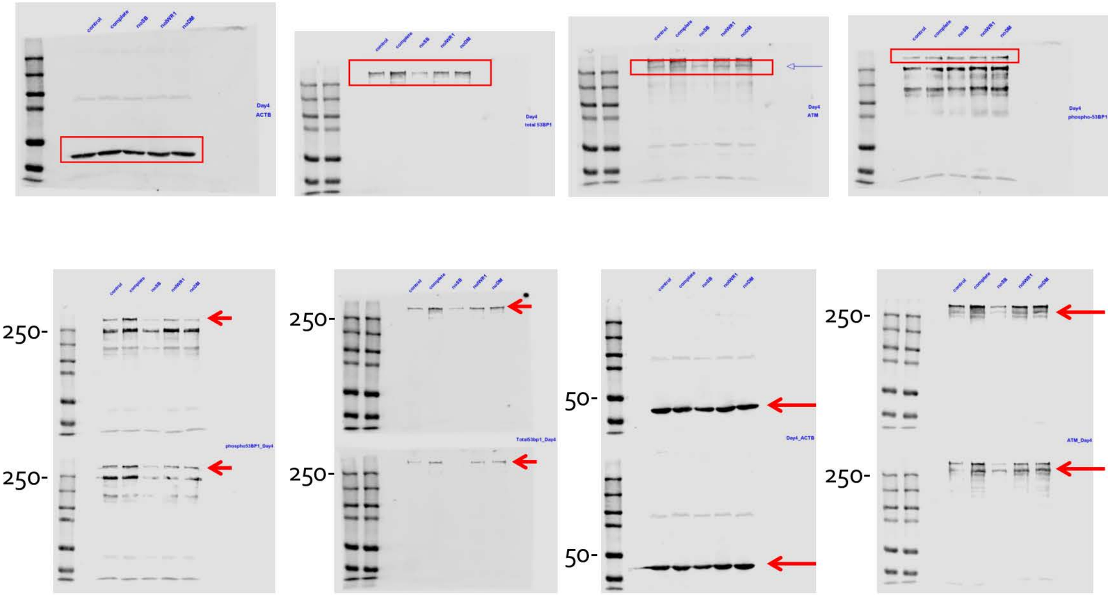

Supplementary Figure 16

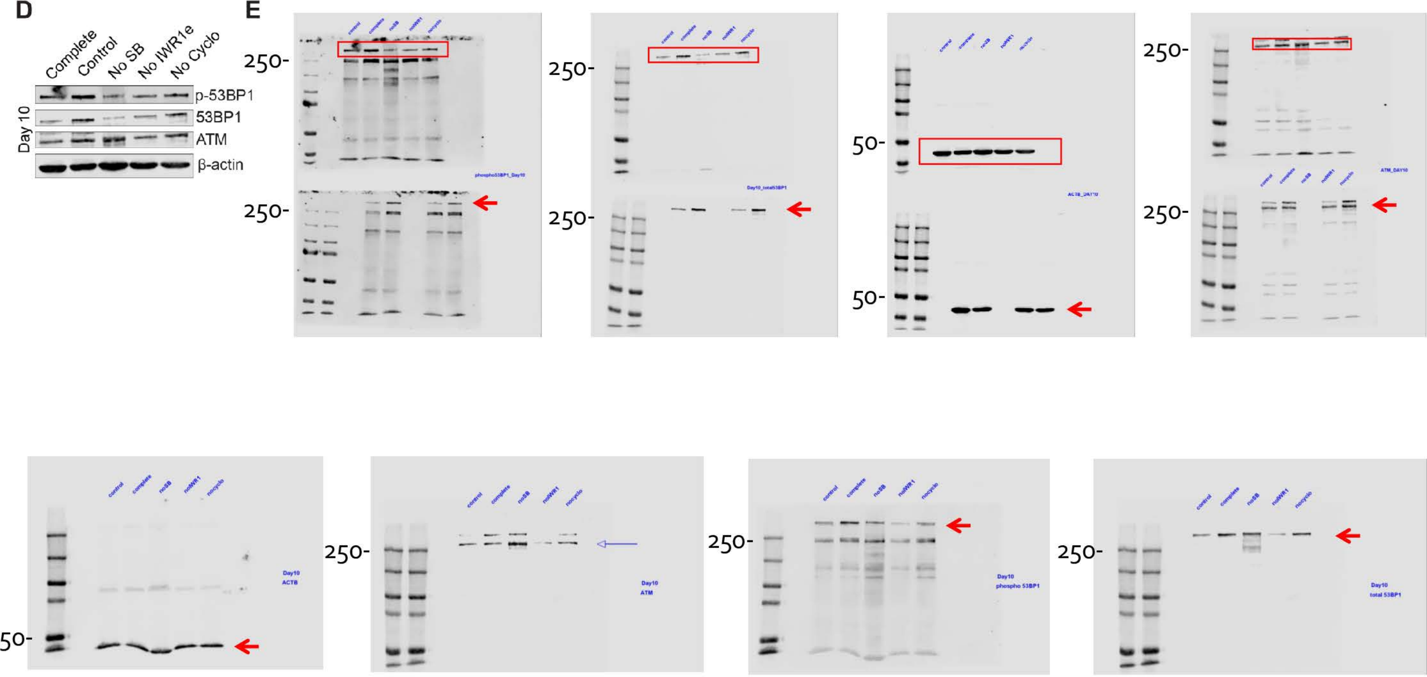

Supplementary Figure 16

B

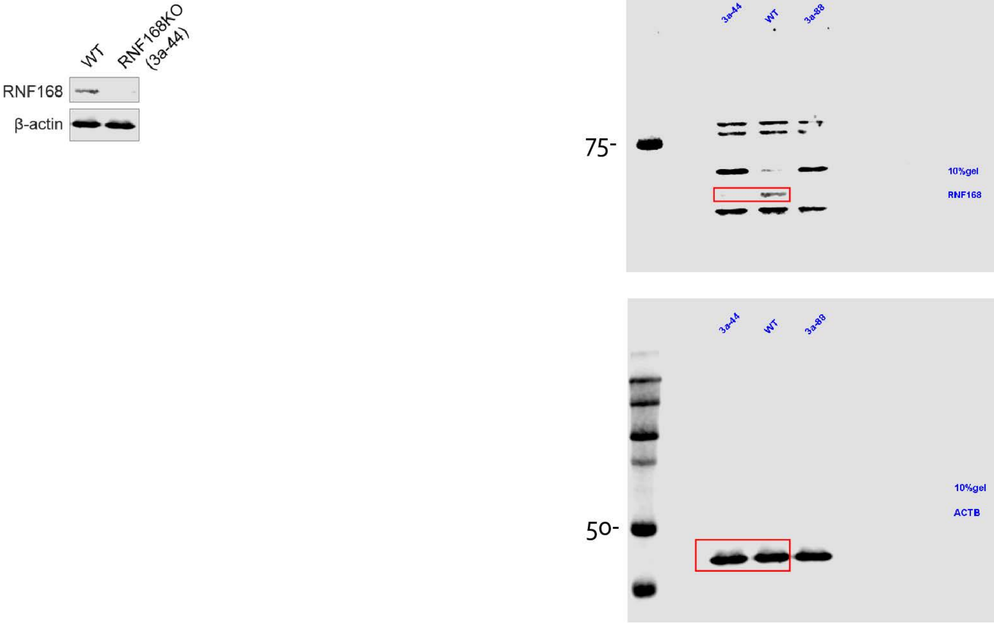

Supplementary Figure 17
