# Supplementary material for: Phosphorylation of the DNA damage repair factor 53BP1 by ATM kinase controls neurodevelopmental programs in cortical brain organoids
Source: PLoS Biol. 2024 Sep 3;22(9):e3002760. doi: 10.1371/journal.pbio.3002760 (PMC11398655; doi:10.1371/journal.pbio.3002760)
Supplement: S1 Fig — (A) Schematic diagram of neural differentiation of hESCs: neural induction, differentiation, and maturation media to form EBs, rosettes, NPCs, and neurons. (B) Principal component analysis of WT ESCs, NPCs, day 10 (D10) cortical organoids, and D17 cortical organoids. GSEA terms that are highly enriched in significantly (C) down-regulated and (D) up-regulated genes in WT NPCs compared to ESCs. % Match, % of genes in the enriched term that overlap the differentially expressed genes or proteins. (E) Immunofluorescence of NPC markers PAX6 and NESTIN. Bar, 50 μm. (F) Quantification of 53BP1-pS25-positive hESCs or hNPCs. Data are presented as the mean ± SEM, with p < 0.0001. (G) WB analysis of control cells and 53BP1-KO clones 415, 416, and 209, which are clones KO1, KO2, and KO3 in Yang and colleagues’ study [6]. (H) WB analysis of control and 53BP1-S25A hNPCs. The S25A mutation prohibits phosphorylation. (I) WB analysis of hESCs and hNPCs and quantification. (J) Schematic diagram of genome editing in hESCs. Guide RNA 6 were complexed with Cas9 proteins and used along single-stranded nucleotide donors to transfect hESCs. Individual clones from transfection were cultured, sequenced by mi-seq across the targeted 53BP1 locus, and established as >99% pure clonal lines. Diagram was generated using open-sourced images available at biorender.com. Underlying numerical values for figures are found in S1 Data. EB, embyoid body; ESC, embryonic stem cell; GSEA, gene set enrichment analysis; hESC, human embryonic stem cell; hNPC, human neural progenitor cell; KO, knockout; NES, normalized enrichment score; NPC, neural progenitor cell; WB, western blot; WT, wild type; 53BP1-pS25, 53BP1 phosphorylated at serine 25. (PDF) [file pbio.3002760.s003.pdf]

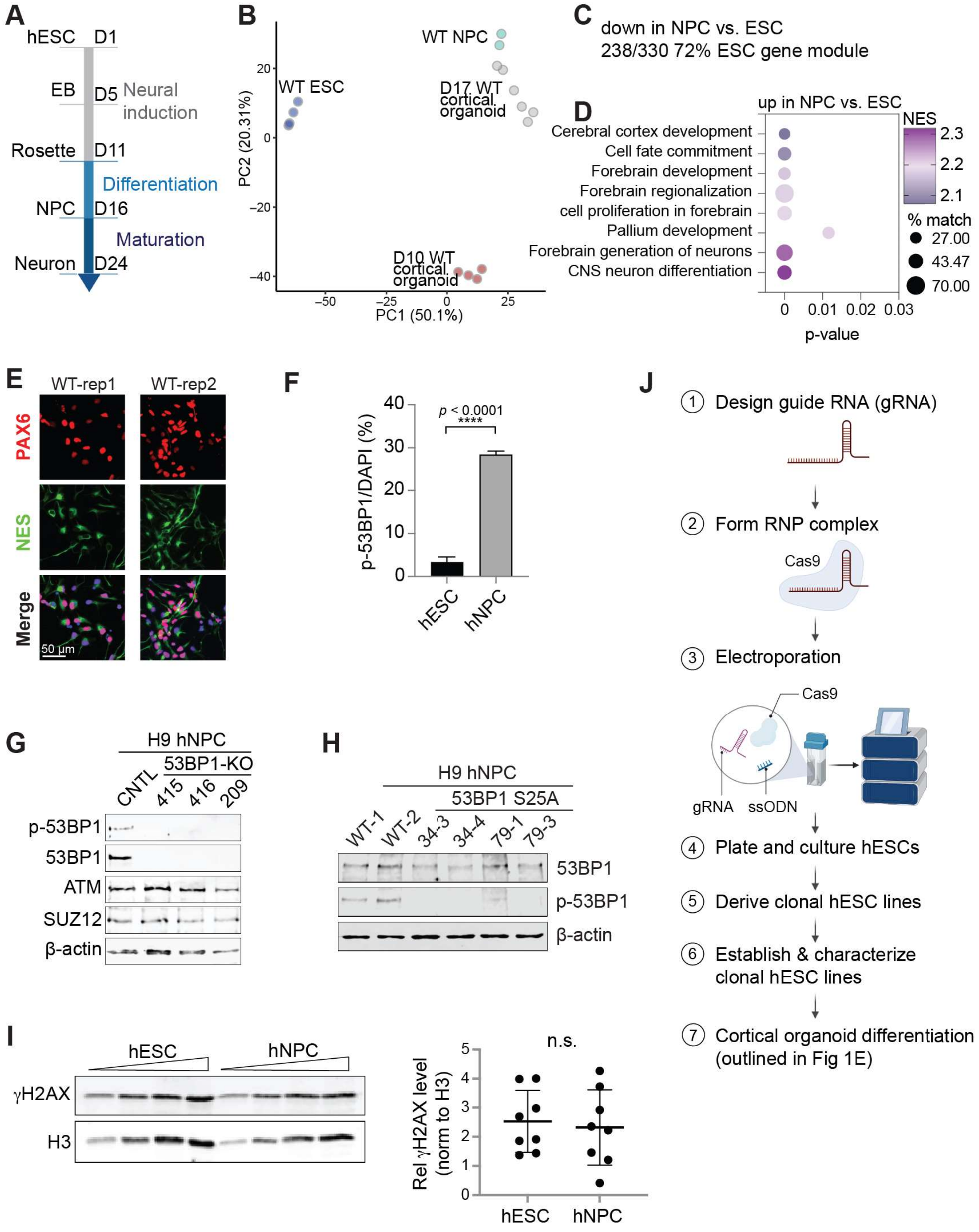

S1 Fig. Characterization of 53BP1-pS25 and NPCs and genome editing of hESCs.
(A) Schematic diagram of neural differentiation of hESCs: neural induction, differentiation, and maturation media to form EBs (embryoid bodies), rosettes, NPCs, and neurons.
(B) Principal component analysis of WT ESCs, NPCs, day 10 (D10) cortical organoids, and D17 cortical organoids.

GSEA terms that are highly enriched in significantly (C) downregulated and (D) upregulated genes in WT NPCs compared to ESCs. NES, normalized enrichment score. % Match, % of genes in the enriched term that overlap the differentially expressed genes or proteins. ESC gene module was published by Wong et al. (PMID 18397753).
(E) Immunofluorescence of NPC markers PAX6 and NESTIN. Bar, 50 μm.
(F) Quantification of 53BP1-pS25-positive hESCs or hNPCs. Data are presented as the mean ± SEM, with $p<0.0001$.
(G) WB analysis of control cells and 53BP1-KO clones 415, 416, and 209, which are clones KO1, KO2, and KO3 in Yang, Xu et al. 2019.
(H) WB analysis of control and 53BP1-S25A hNPCs. The S25A mutation prohibits phosphorylation.
(I) WB analysis of hESCs and hNPCs and quantification.
(J) Schematic diagram of genome editing in hESCs. Guide RNA 6 were complexed with Cas9 proteins and used along single-stranded nucleotide donors to transfect hESCs. Individual clones from transfection were cultured, sequenced by mi-seq across the targeted *53BP1* locus, and established as >99% pure clonal lines. Diagram was generated using open-sourced images available at biorender.com.
Underlying numerical values for figures are found in S1_Data.xlsx.
