# Supplementary material for: Phosphorylation of the DNA damage repair factor 53BP1 by ATM kinase controls neurodevelopmental programs in cortical brain organoids
Source: PLoS Biol. 2024 Sep 3;22(9):e3002760. doi: 10.1371/journal.pbio.3002760 (PMC11398655; doi:10.1371/journal.pbio.3002760)
Supplement: S2 Fig — (A) Alignment of WT and ATM-KO mutation sequences on 2 alleles (al) in the ATM locus. Red indicates the gRNA sequence. (B) WB analysis of WT and 4 ATM-KO hNPCs. (C) Principal component analysis showed the intermixing and similar RNA-seq profiles from hESCs of 7 WT, 4 53BP1-S25A, 4 53BP1-S25D, 4 ATM-KO, and 4 53BP1-KO lines. (D) Immunofluorescence showed similar expression of OCT4 and SSEA4 proteins in control and ATM-KO hESCs. Bar, 100 μm. (E) WB analysis of WT and 2 ATM-KO hNPCs. Quantification suggests reduction of γH2AX in ATM-KO hNPCs. Welch’s t test was used to perform pairwise comparisons of WT and ATM-KO. Underlying numerical values for figures are found in S1 Data. ATM, ataxia telangiectasia mutated; hESC, human embryonic stem cell; hNPC, human neural progenitor cell; KO, knockout; WB, western blot; WT, wild type. (PDF) [file pbio.3002760.s004.pdf]

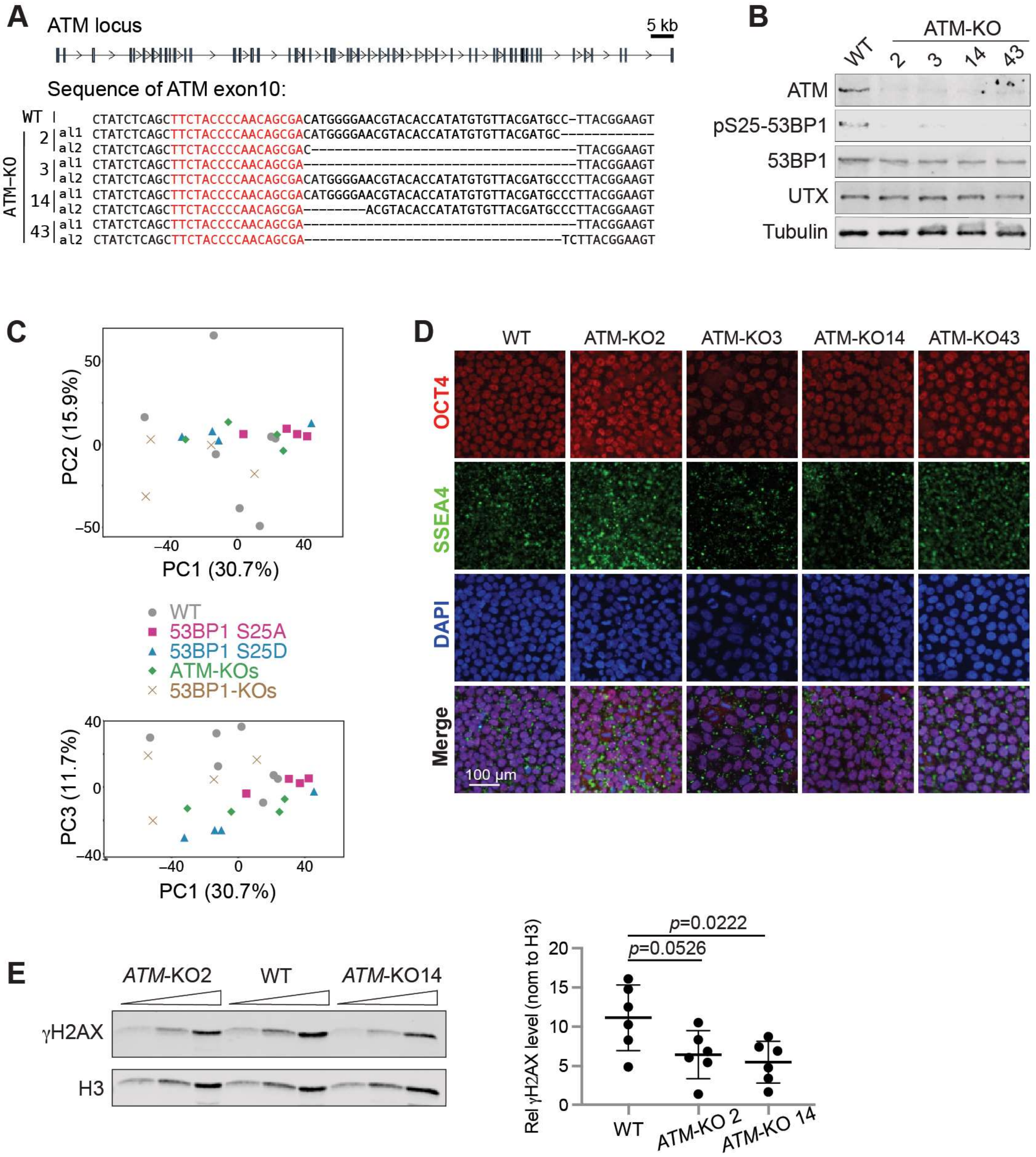

S2 Fig. Generation and analyses of ATM-KO hESCs and cortical organoids.

(A) Alignment of WT and *ATM*-KO mutation sequences on 2 alleles (al) in the *ATM* locus. Red indicates the gRNA sequence.

(B) WB analysis of WT and 4 *ATM*-KO hNPCs.

(C) Principal component analysis showed the intermixing and similar RNA-seq profiles from hESCs of 7 WT, 4 53BP1-S25A, 4 53BP1-S25D, 4 ATM-KO, and 4 53BP1-KO lines.

(D) Immunofluorescence showed similar expression of OCT4 and SSEA4 proteins in control and *ATM*-KO hESCs. Bar, 100 µm.

(E) WB analysis of WT and 2 *ATM*-KO hNPCs. Quantification suggests reduction of γH2AX in *ATM*-KO hNPCs. Welch's t test was used to perform pairwise comparisons of WT and *ATM*-KO.

Underlying numerical values for figures are found in S1_Data.xlsx.
