# Supplementary material for: Phosphorylation of the DNA damage repair factor 53BP1 by ATM kinase controls neurodevelopmental programs in cortical brain organoids
Source: PLoS Biol. 2024 Sep 3;22(9):e3002760. doi: 10.1371/journal.pbio.3002760 (PMC11398655; doi:10.1371/journal.pbio.3002760)
Supplement: S3 Fig — (A) Immunofluorescence showed D35 ATM-KO and WT cortical organoids had similar γH2AX foci. Bar, 100 μm. FACS analysis of CC3 in (B) D21 and (C) D28 cortical organoids. Two biological replicates were done, and each data point was based on 3 technical replicate analyses of 10–12 cortical organoids. (D, E) Immunofluorescence and quantification of CC3 in D28 cortical organoids. Bar, 100 μm. Graphs are presented in ratios (out of 1), with **, p < 0.01; ****, p < 0.0001; ns, not significant by two-way ANOVA test. Underlying numerical values for figures are found in S1 Data. ATM, ataxia telangiectasia mutated; CC3, cleaved-caspase 3; KO, knockout; WT, wild type. (PDF) [file pbio.3002760.s005.pdf]

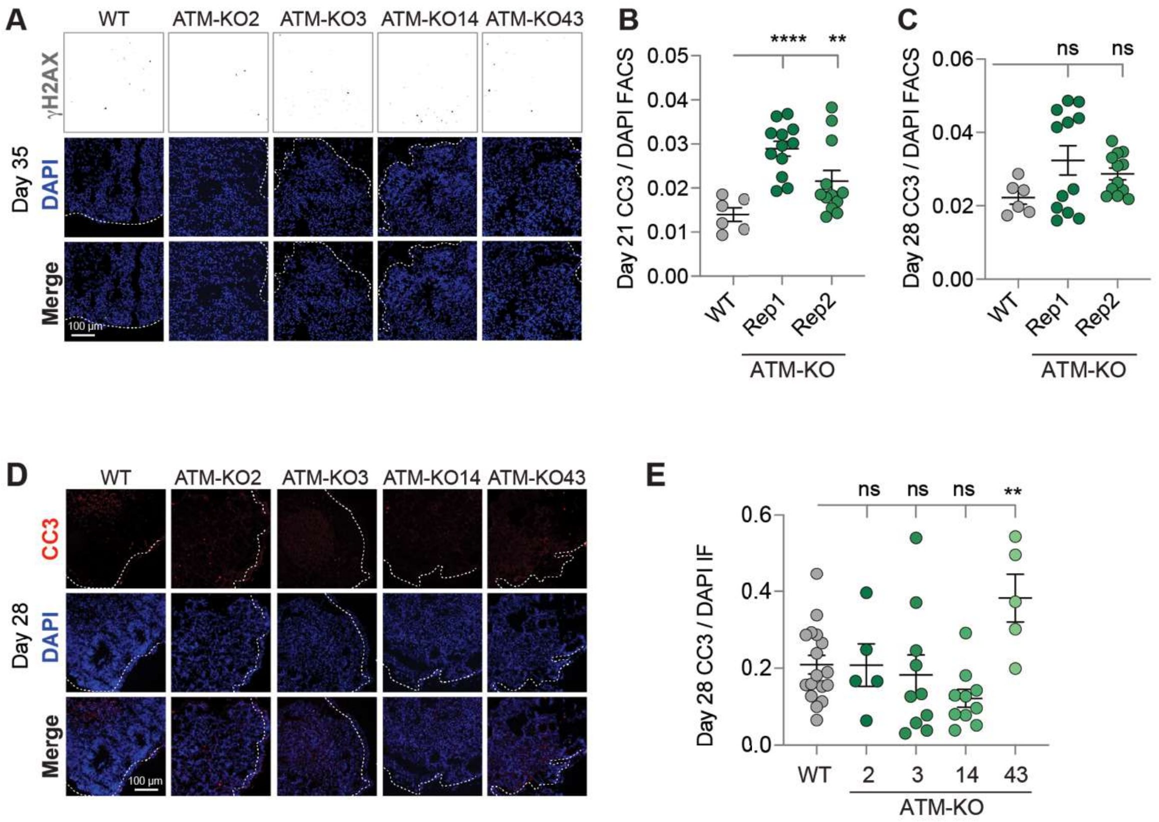

S3 Fig. Analysis of γH2AX and cleaved-caspase 3 (CC3) in cortical organoids.

(A) Immunofluorescence showed D35 *ATM*-KO and WT cortical organoids had similar γH2AX foci. Bar, 100 µm.

FACS analysis of CC3 in (B) D21 and (C) D28 cortical organoids. Two biological replicates were done, and each data point was based on 3 technical replicate analyses of 10-12cortical organoids.

(D, E) Immunofluorescence and quantification of CC3 in D28 cortical organoids. Bar, 100 µm.

Graphs are presented in ratios (out of 1), with **, $p<0.01$; ****, $p<0.0001$; ns, not significant by Two-way ANOVA test.

Underlying numerical values for figures are found in S1_Data.xlsx.
