# Supplementary material for: Phosphorylation of the DNA damage repair factor 53BP1 by ATM kinase controls neurodevelopmental programs in cortical brain organoids
Source: PLoS Biol. 2024 Sep 3;22(9):e3002760. doi: 10.1371/journal.pbio.3002760 (PMC11398655; doi:10.1371/journal.pbio.3002760)
Supplement: S4 Fig — FACS analysis of PAX6 and KI67 in (A, B) D28 and (C) D35 cortical organoids. Each data point was based on the 3 technical replicate analyses of 10 cortical organoids. (D) Quantification of KI67/PAX6 ratios in immunofluorescence of D35 cortical organoids. Each data point represents quantification of cells in 1 cortical organoid. (E, F) Immunofluorescence and quantification of H3-pS10 (PH3) in D28 cortical organoids. Bar, 100 μm. (F-H) Immunofluorescence and quantification of PH3 and KI67 in D35 cortical organoids. Bar, 100 μm. ***, p < 0.001; ns, not significant by two-way ANOVA test. Underlying numerical values for figures are found in S1 Data. (PDF) [file pbio.3002760.s006.pdf]

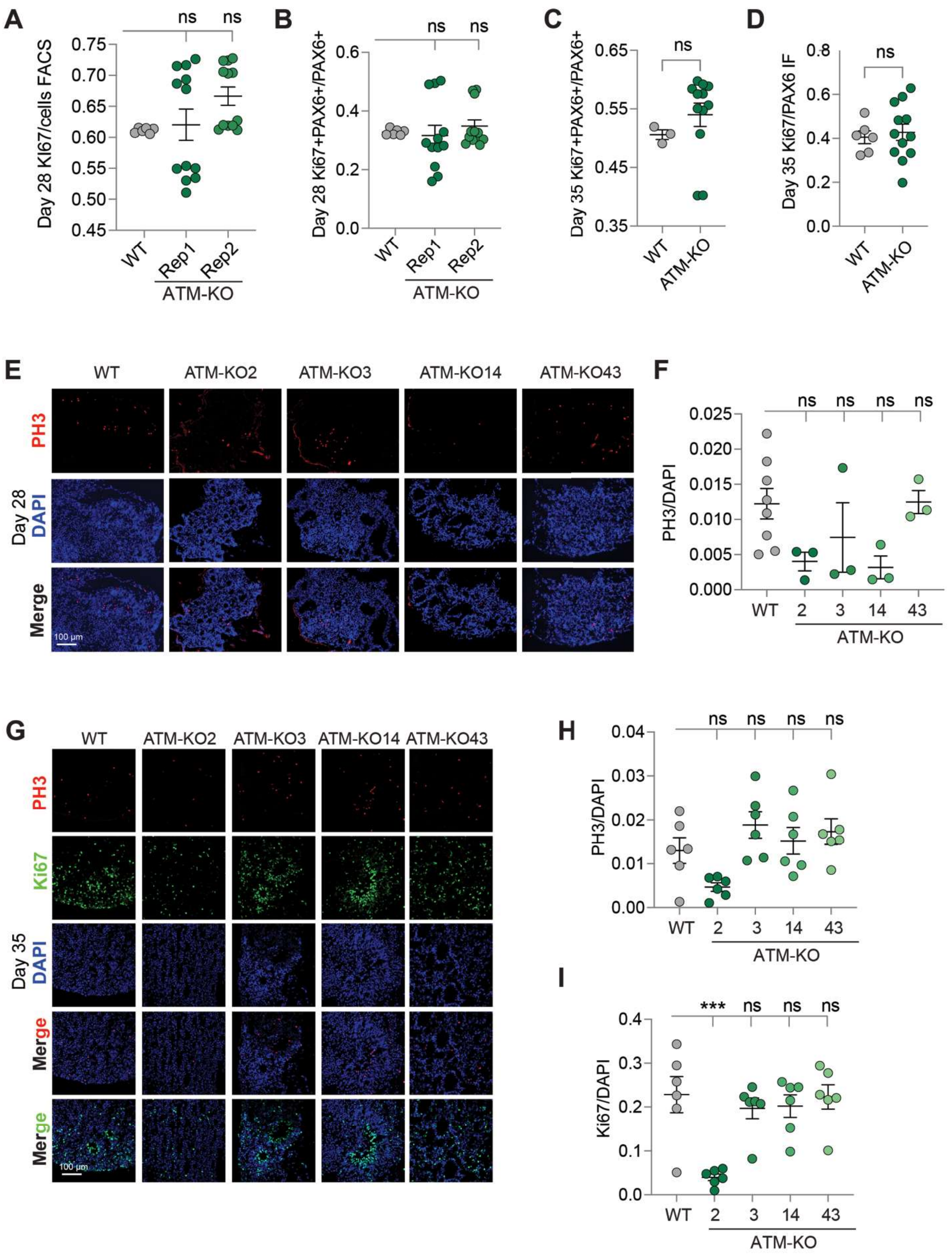

S4 Fig. Analysis of cell proliferation in cortical organoids.

FACS analysis of PAX6 and KI67 in (A, B) D28 and (C) D35 cortical organoids. Each data point was based on the 3 technical replicate analyses of 10 cortical organoids.

(D) Quantification of KI67/PAX6 ratios in immunofluorescence of D35 cortical organoids. Each data point represents quantification of cells in 1 cortical organoid.
(E, F) Immunofluorescence and quantification of H3-pS10 (PH3) in D28 cortical organoids. Bar, 100 μm.
(F-H) Immunofluorescence and quantification of PH3 and KI67 in D35 cortical organoids. Bar, 100 μm.
***, $p<0.001$; ns, not significant by Two-way ANOVA test.
Underlying numerical values for figures are found in S1_Data.xlsx.
