# Supplementary material for: Phosphorylation of the DNA damage repair factor 53BP1 by ATM kinase controls neurodevelopmental programs in cortical brain organoids
Source: PLoS Biol. 2024 Sep 3;22(9):e3002760. doi: 10.1371/journal.pbio.3002760 (PMC11398655; doi:10.1371/journal.pbio.3002760)
Supplement: S5 Fig — Immunofluorescence of (A) NEUN and (E) ZO-1 and PAX6 in D37 cortical organoids. Bar, 100 μm. (B) Immunofluorescence of ZO-1 in D28 cortical organoids. Bar, 100 μm. Quantification of the (C) number and (D) surface area of ZO-1-positive ventricles in D28 cortical organoids. *, p < 0.05; ***, p <0 .001; ns, not significant by two-way ANOVA test. (F) Bright-field images of cortical organoids formed by ATM-KO2, 3, 14, 43, and WT control at day 55 of differentiation. Bar, 1.5 mm. (G) The size of cortical organoids was compared between groups by one-way ANOVA with Dunnett’s multiple comparisons test, with ns, not significant and ***, p < 0.001. n = 13 organoids/group. Underlying numerical values for figures are found in S1 Data. ATM, ataxia telangiectasia mutated; NPC, neural progenitor cell; WT, wild type. (PDF) [file pbio.3002760.s007.pdf]

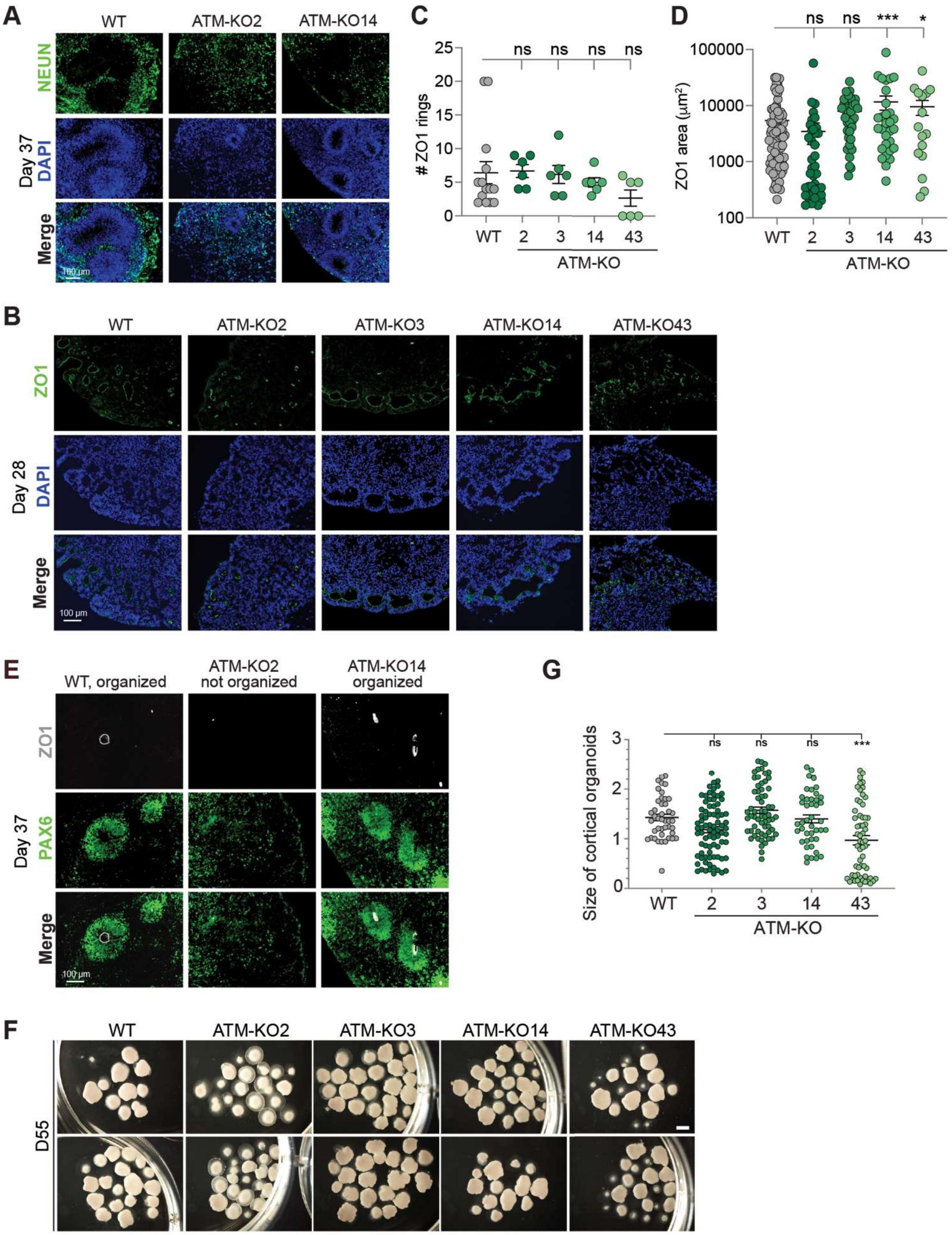

S5 Fig. Immunofluorescence analyses of cortical organoids and NPCs.

Immunofluorescence of (A) NEUN and (E) ZO-1 and PAX6 in D37 cortical organoids. Bar, 100 µm.

(B) Immunofluorescence of ZO-1 in D28 cortical organoids. Bar, 100 μm.
Quantification of the (C) number and (D) surface area of ZO-1-positive ventricles in D28 cortical organoids. *, $p<0.05$; ***, $p<0.001$; ns, not significant by Two-way ANOVA test.
(F) Bright-field images of cortical organoids formed by *ATM*-KO2, 3, 14, 43, and WT control at day 55 of differentiation. Bar, 1.5 mm.
(G) The size of cortical organoids was compared between groups by one-way ANOVA with Dunnett's multiple comparisons test, with ns, not significant and ***, $p<0.001$. n = 13 organoids/group.
Underlying numerical values for figures are found in S1_Data.xlsx.
