# Supplementary material for: Phosphorylation of the DNA damage repair factor 53BP1 by ATM kinase controls neurodevelopmental programs in cortical brain organoids
Source: PLoS Biol. 2024 Sep 3;22(9):e3002760. doi: 10.1371/journal.pbio.3002760 (PMC11398655; doi:10.1371/journal.pbio.3002760)
Supplement: S6 Fig — (A) Immunofluorescence of PAX6 and NES in NPCs. Bar, 50 μm. (B) Principal component analysis of proteomics data of D35 WT and ATM-KO cortical organoids. GSEA terms that are highly enriched in significantly (C) higher and (D) lower total proteins in D35 ATM-KO versus WT cortical organoids. (E) GSEA terms that are highly enriched in significantly higher phosphoproteins, which were normalized to total proteomics, in D35 ATM-KO versus WT cortical organoids. Underlying numerical values for figures are found in S1 Data. ATM, ataxia telangiectasia mutated; GSEA, gene set enrichment analysis; KO, knockout; NES, normalized enrichment score; NPC, neural progenitor cell; WT, wild type. (PDF) [file pbio.3002760.s008.pdf]

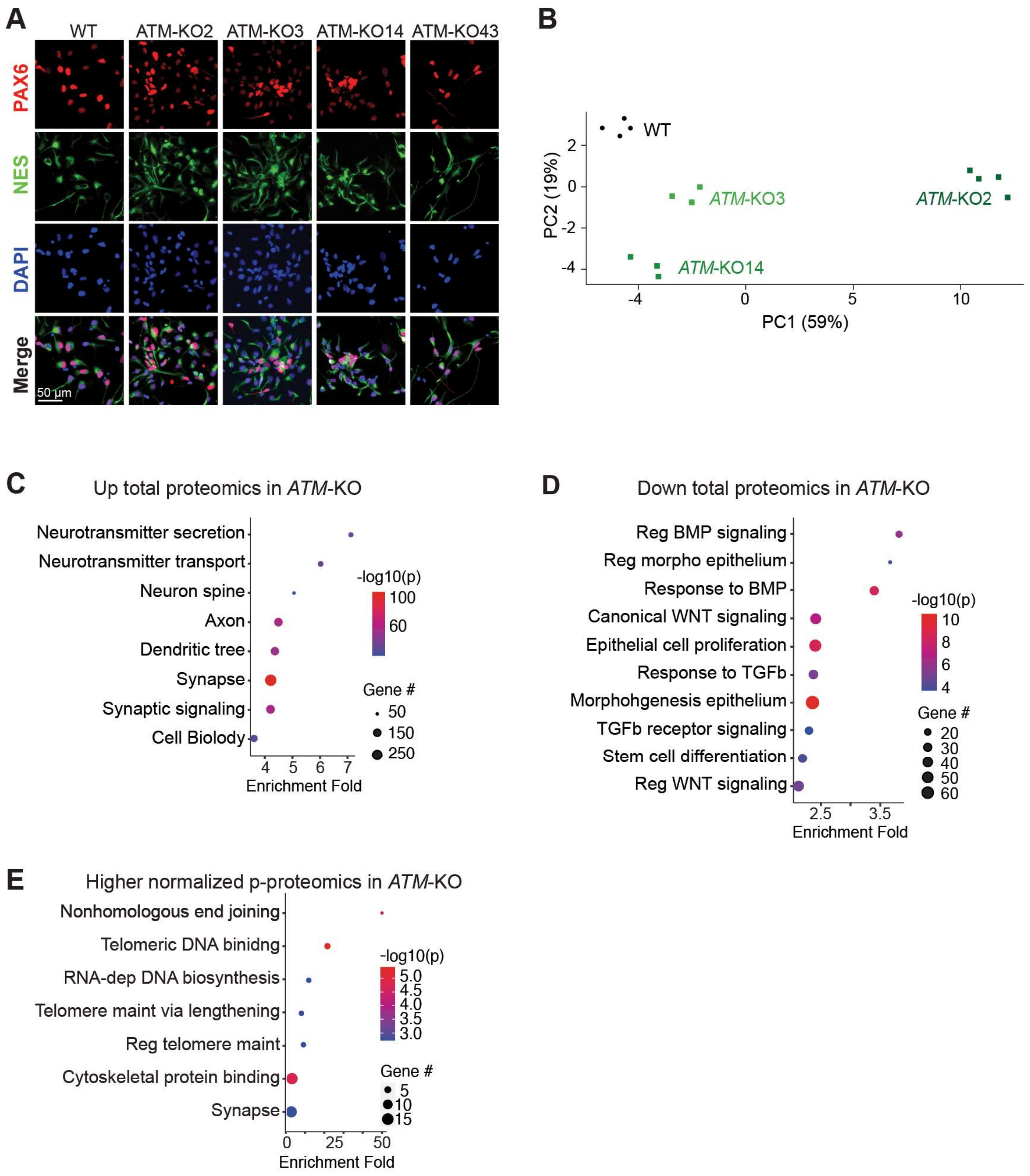

S6 Fig. Characterization of NPCs and D35 cortical organoids.

(A) Immunofluorescence of PAX6 and NES in NPCs. Bar, 50 µm.

(B) Principal component analysis of proteomics data of D35 WT and *ATM*-KO cortical organoids.

GSEA terms that are highly enriched in significantly (C) higher and (D) lower total proteins in D35 *ATM*-KO versus WT cortical organoids.

(E) GSEA terms that are highly enriched in significantly higher phospho-proteins, which were normalized to total proteomics, in D35 *ATM*-KO versus WT cortical organoids.

Underlying numerical values for figures are found in S1_Data.xlsx.
