# Supplementary material for: Phosphorylation of the DNA damage repair factor 53BP1 by ATM kinase controls neurodevelopmental programs in cortical brain organoids
Source: PLoS Biol. 2024 Sep 3;22(9):e3002760. doi: 10.1371/journal.pbio.3002760 (PMC11398655; doi:10.1371/journal.pbio.3002760)
Supplement: S7 Fig — Heatmaps showing relative phosphorylation levels of (A) 7 MAPK9 substrates that are significantly lower and (B) 7 CDK5 substrates that are significantly higher in D35 ATM-KO versus WT cortical organoids. (C) Heatmaps showing activity of selected protein kinases between ATM-KO3, ATM-KO4, and WT cell lines. (D) Alignment of WT and 53BP1-S25A and S25D mutation sequences on 2 alleles (al). Red indicates the gRNA sequence. Underline indicates codon encoding the WT serine 25, mutant alanine, or mutant aspartic acid. (E) WB analysis of control and 53BP1-S25D hNPCs, which have comparable levels of 53BP1 protein. (F) Transcripts per million values of 10 pluripotency genes were used for comparison to show that control, 53BP1-S25A, and 53BP1-S25D hESCs did not differ in pluripotency. Underlying numerical values for figures are found in S1 Data. ATM, ataxia telangiectasia mutated; hESC, human embryonic stem cell; hNPC, human neural progenitor cell; KO, knockout; WB, western blot; WT, wild type; 53BP1, p53 binding protein 1. (PDF) [file pbio.3002760.s009.pdf]

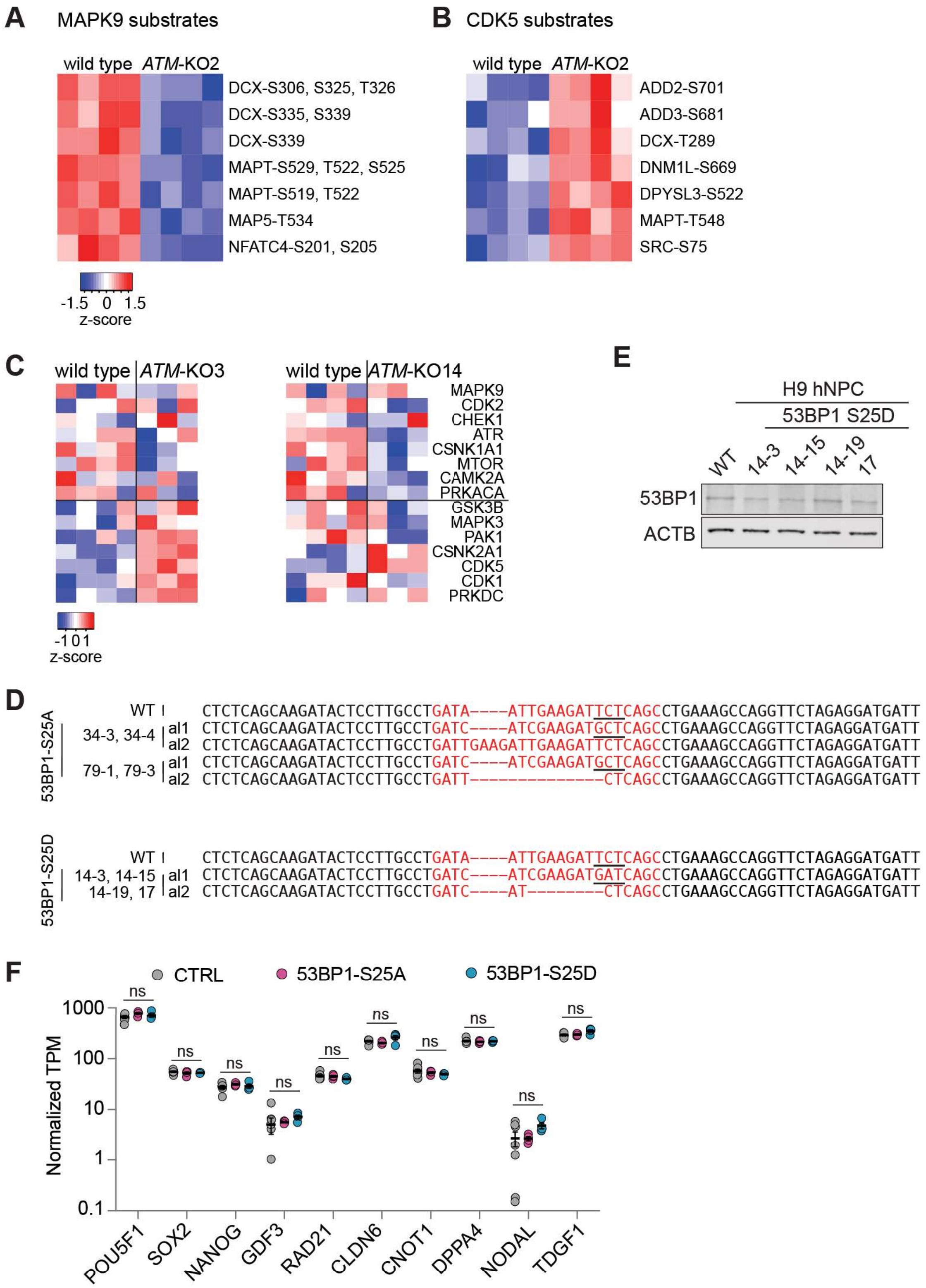

S7 Fig. Kinase activities in cortical organoids and characterization of the 53BP1-S25A and -S25D hESCs.

Heatmaps showing relative phosphorylation levels of (A) 7 MAPK9 substrates that are significantly lower and (B) 7 CDK5 substrates that are significantly higher in D35 *ATM*-KO versus WT cortical organoids.

(C) Heatmaps showing activity of selected protein kinases between ATM-KO3, ATM-KO4, and wild type cell lines.
(D) Alignment of WT and 53BP1-S25A and S25D mutation sequences on 2 alleles (al). Red indicates the gRNA sequence. Underline indicates codon encoding the wild-type serine 25, mutant alanine, or mutant aspartic acid.
(E) WB analysis of control and 53BP1-S25D hNPCs, which have comparable levels of 53BP1 protein.
(F) Transcripts per million values of 10 pluripotency genes were used for comparison to show that control, 53BP1-S25A, and 53BP1-S25D hESCs did not differ in pluripotency.
Underlying numerical values for figures are found in S1_Data.xlsx.
