# Supplementary material for: Phosphorylation of the DNA damage repair factor 53BP1 by ATM kinase controls neurodevelopmental programs in cortical brain organoids
Source: PLoS Biol. 2024 Sep 3;22(9):e3002760. doi: 10.1371/journal.pbio.3002760 (PMC11398655; doi:10.1371/journal.pbio.3002760)
Supplement: S8 Fig — (A) Immunofluorescence showed similar expression of OCT4 and SSEA4 proteins in WT, 53BP1-S25A, and 53BP1-S25D hESCs. Bar, 100 μm. Immunofluorescence of (B) KI67 and (D) PH3 in cryosections of cortical organoids at day 35 of differentiation. Bar, 100 μm. (C) Quantification of KI67-positive cells in D35 cortical organoids. Data points represent single organoids. The mean ± SEM values were compared by one-way ANOVA with Dunnett’s multiple comparisons test to yield ****, ***, and ** indicating p < 0.0001, 0.001, and 0.01, respectively. n = 3 organoids/group. Underlying numerical values for figures are found in S1 Data. (PDF) [file pbio.3002760.s010.pdf]

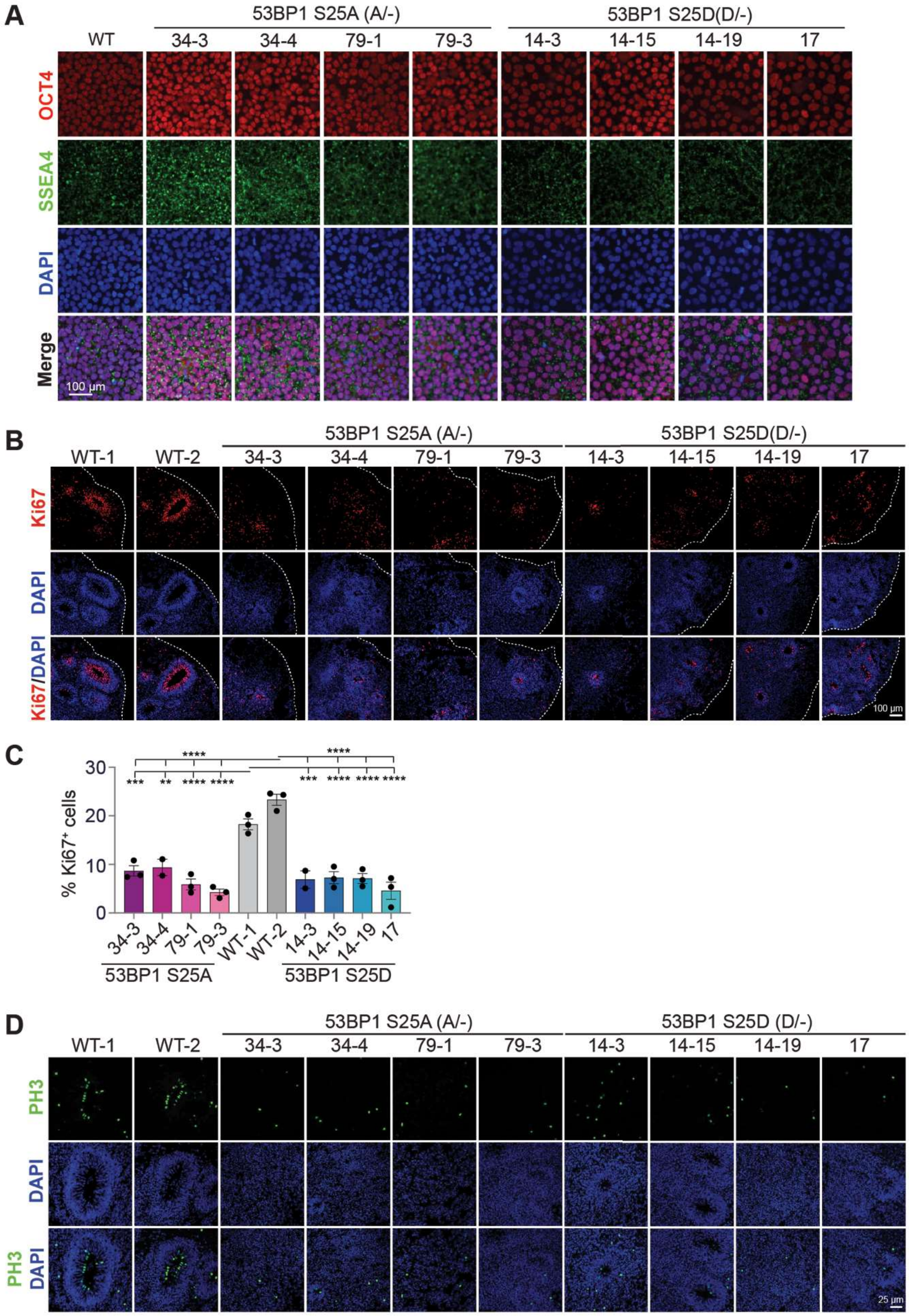

S8 Fig. Characterization of the 53BP1-S25A and -S25D hESCs and cortical organoids.

(A) Immunofluorescence showed similar expression of OCT4 and SSEA4 proteins in WT, 53BP1-S25A, and 53BP1-S25D hESCs. Bar, 100 μm.
Immunofluorescence of (B) KI67 and (D) PH3 in cryosections of cortical organoids at day 35 of differentiation. Bar, 100 μm.
(C) Quantification of KI67-positive cells in D35 cortical organoids. Data points represent single organoids. The mean ± SEM values were compared by one-way ANOVA with Dunnett's multiple comparisons test to yield ****, ***, and ** indicating $p < 0.0001$, 0.001, and 0.01, respectively. n = 3 organoids/group.
Underlying numerical values for figures are found in S1_Data.xlsx.
