# Supplementary material for: Phosphorylation of the DNA damage repair factor 53BP1 by ATM kinase controls neurodevelopmental programs in cortical brain organoids
Source: PLoS Biol. 2024 Sep 3;22(9):e3002760. doi: 10.1371/journal.pbio.3002760 (PMC11398655; doi:10.1371/journal.pbio.3002760)
Supplement: S9 Fig — Immunofluorescence of (A) γH2AX in D35 cortical organoids and (D) CC3 in D28 cortical organoids. Bar, 100 μm. CC3 quantification by FACS of (B) D21 and (C) D28 cortical organoids. For each datapoint, 10–12 organoids from each line were analyzed via 3 technical replicates, and data from 4 mutant lines were consolidated to achieve rigorous comparisons. **, p < 0.01 and ns, not significant by two-way ANOVA test. (E) CC3 quantification of immunofluorescence images of D28 cortical organoids. For each line, 4–6 images and >10,000 cells were analyzed. *, p < 0.05; **, p < 0.01; ns, not significant by two-way ANOVA test. Graphs in (B, C, E) are presented in ratios (out of 1). Underlying numerical values for figures are found in S1 Data. (PDF) [file pbio.3002760.s011.pdf]

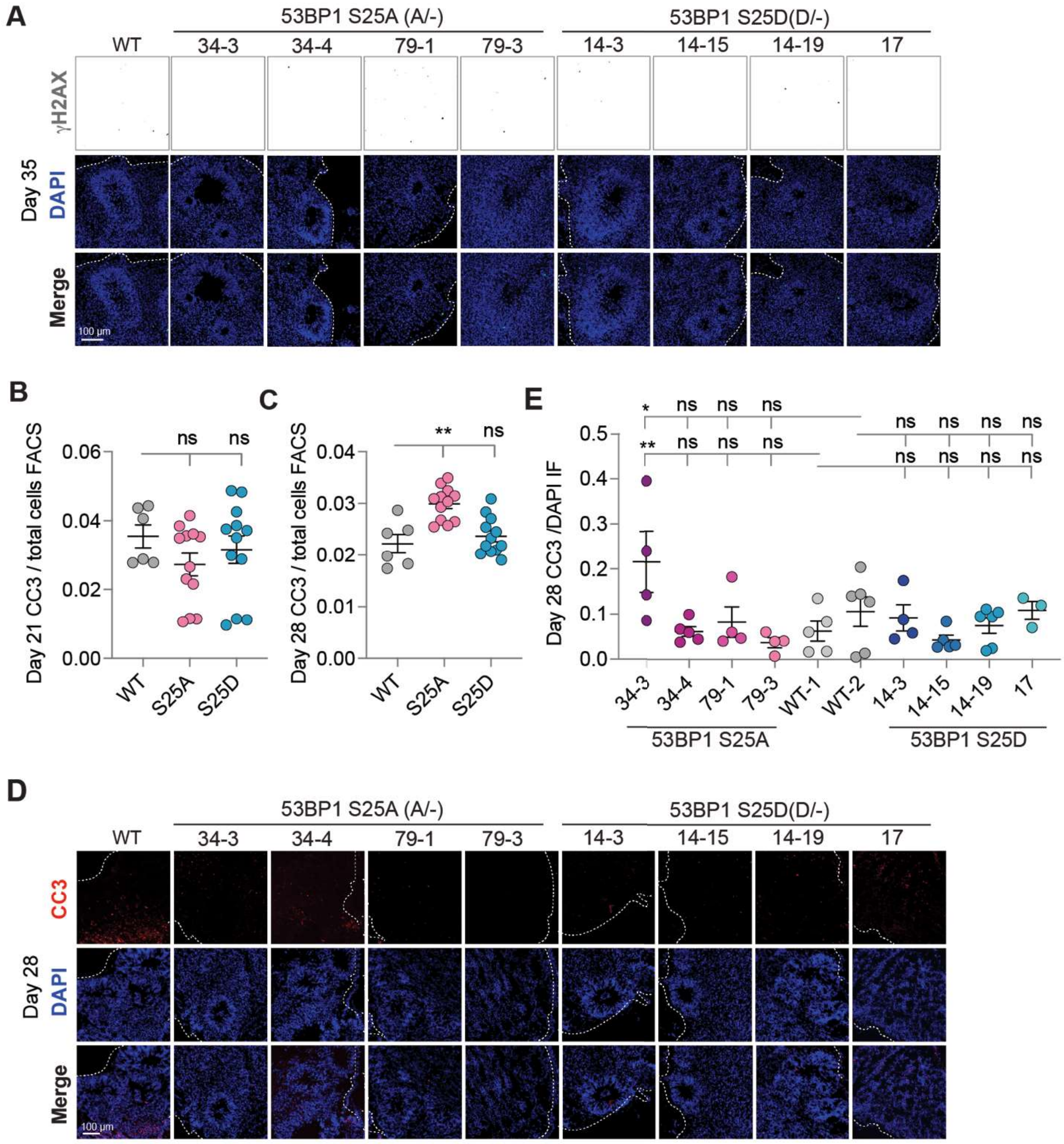

S9 Fig. Analysis of γH2AX and cleaved-caspase 3 (CC3) in cortical organoids.

Immunofluorescence of (A) γH2AX in D35 cortical organoids and (D) cleaved caspase 3 (CC3) in D28 cortical organoids. Bar, 100 μm.

CC3 quantification by FACS of (B) D21 and (C) D28 cortical organoids. For each datapoint, 10-12 organoids from each line were analyzed via 3 technical replicates, and data from 4 mutant lines were consolidated to achieve rigorous comparisons. **, p<0.01 and ns, not significant by Two-way ANOVA test.

(E) CC3 quantification of immunofluorescence images of D28 cortical organoids. For each line, 4-6 images and >10,000 cells were analyzed. *, p<0.05; **, p<0.01; ns, not significant by Two-way ANOVA test.

Graphs in (B, C, E) are presented in ratios (out of 1).

Underlying numerical values for figures are found in S1_Data.xlsx.
