# Supplementary material for: Phosphorylation of the DNA damage repair factor 53BP1 by ATM kinase controls neurodevelopmental programs in cortical brain organoids
Source: PLoS Biol. 2024 Sep 3;22(9):e3002760. doi: 10.1371/journal.pbio.3002760 (PMC11398655; doi:10.1371/journal.pbio.3002760)
Supplement: S10 Fig — Quantification of the (A) number and (B) surface area of ZO-1-positive ventricles. (C) Immunofluorescence of ZO-1 in D28 cortical organoids. Bar, 100 μm. *, p < 0.05; **, p < 0.01; ***, p < 0.001; ns, not significant by two-way ANOVA test. Underlying numerical values for figures are found in S1 Data. (PDF) [file pbio.3002760.s012.pdf]

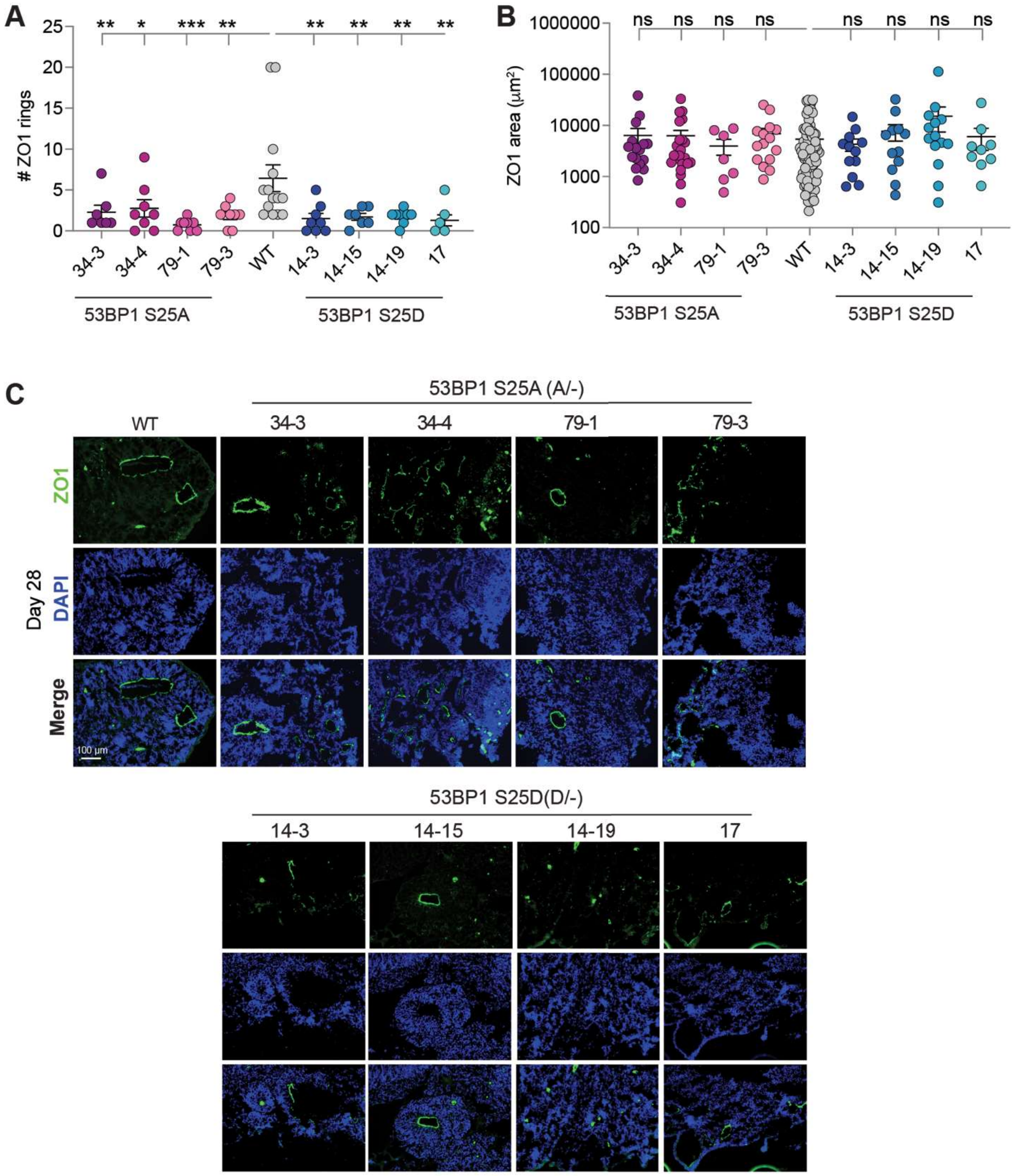

S10 Fig. 53BP1-pS25 promotes ventricle formation in cortical organoids.
Quantification of the (A) number and (B) surface area of ZO-1-positive ventricles.
(C) Immunofluorescence of ZO-1 in D28 cortical organoids. Bar, 100 μm.
*, $p<0.05$; **, $p<0.01$; ***, $p<0.001$; ns, not significant by Two-way ANOVA test.
Underlying numerical values for figures are found in S1_Data.xlsx.
