# Supplementary material for: Phosphorylation of the DNA damage repair factor 53BP1 by ATM kinase controls neurodevelopmental programs in cortical brain organoids
Source: PLoS Biol. 2024 Sep 3;22(9):e3002760. doi: 10.1371/journal.pbio.3002760 (PMC11398655; doi:10.1371/journal.pbio.3002760)
Supplement: S11 Fig — (A) Bright-field images of cortical organoids formed by cell lines 53BP1-S25A 34–3, 34–4, 79–1, 79–3 and S25D 14–3, 14–15, 14–19, 17, and 2 WT control at day 55 of differentiation. Bar, 1.5 mm. Blue transparent structures around organoids are Matrigel embedment. (B) At day 55 of differentiation, the size of cortical organoids was compared between groups. (C) The growth (comparing organoids at days 35 and 55) of cortical organoids were compared between groups. Data points represent single organoids. The mean ± SEM values were compared by one-way ANOVA with Dunnett’s multiple comparisons test to yield **** and ** indicating p < 0.0001 and 0.01, respectively. n = 15–36 organoids/group. (D) Two genes overlapped between up-regulated genes in 53BP1-S25A versus WT and down-regulated genes in 53BP1-S25D versus WT cortical organoids. No gene overlapped between down-regulated genes in 53BP1-S25A versus WT and up-regulated genes in 53BP1-S25D versus WT cortical organoids. (E) Down-regulated GSEA terms between 53BP1-S25A versus WT and 53BP1-S25D versus WT were not highly overlapped. Ten GSEA terms were specific to 53BP1-S25D versus WT. Underlying numerical values for figures are found in S1 Data. (PDF) [file pbio.3002760.s013.pdf]

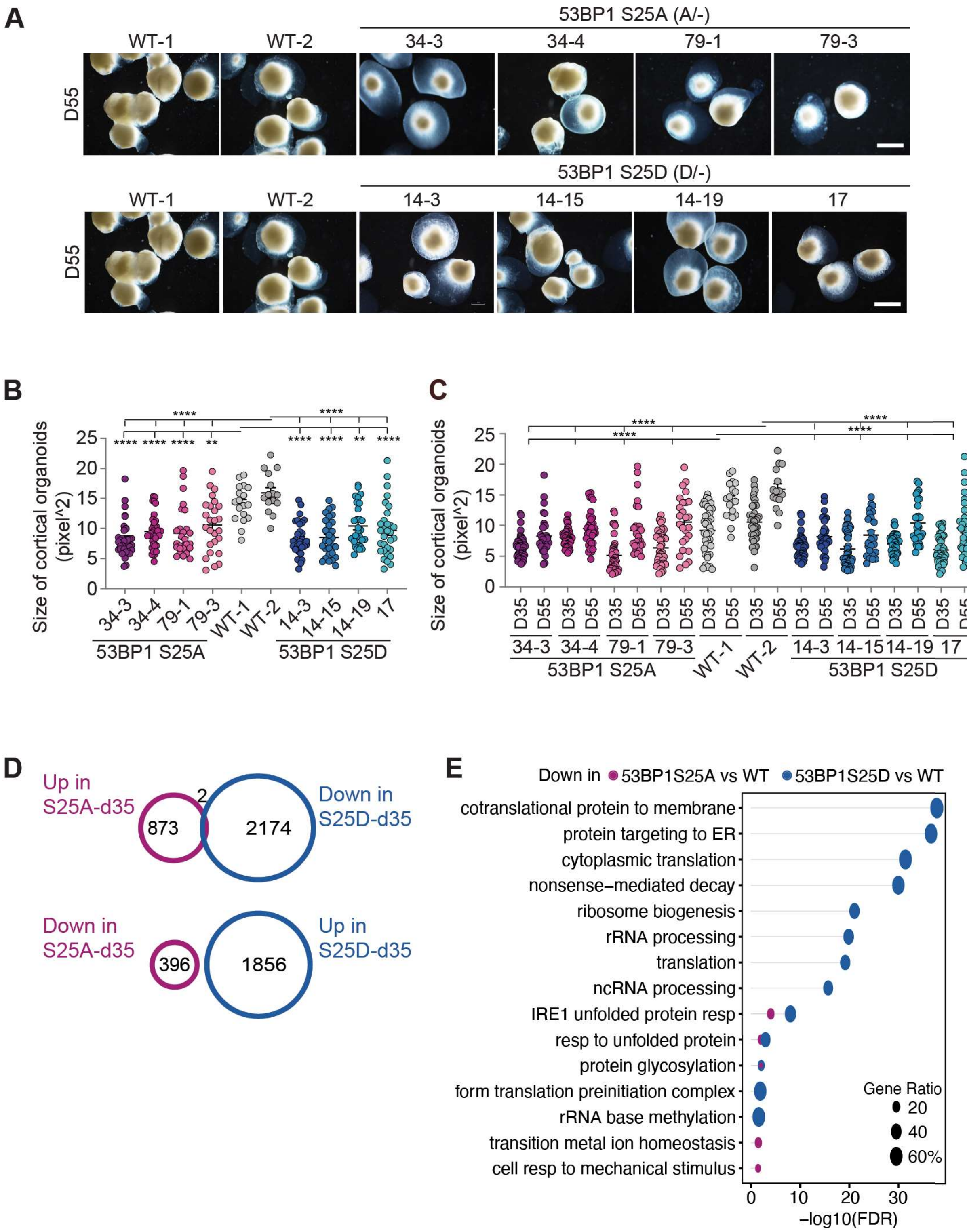

S11 Fig. Characterization of 53BP1-S25A and -S25D cortical organoids.

(A) Bright-field images of cortical organoids formed by cell lines 53BP1-S25A 34-3, 34-4, 79-1, 79-3 and S25D 14-3, 14-15, 14-19, 17, and 2 WT control at day 55 of differentiation. Bar, 1.5 mm. Blue transparent structures around organoids are Matrigel embedment.

(B) At day 55 of differentiation, the size of cortical organoids was compared between groups.

(C) The growth (comparing organoids at days 35 and 55) of cortical organoids were compared between groups. Data points represent single organoids. The mean ± SEM values were compared by one-way ANOVA with Dunnett's multiple comparisons test to yield **** and ** indicating p< 0.0001 and 0.01, respectively. n = 15 – 36 organoids/group.
(D) Two genes overlapped between upregulated genes in 53BP1-S25A versus WT and downregulated genes in 53BP1-S25D versus WT cortical organoids. No gene overlapped between downregulated genes in 53BP1-S25A versus WT and upregulated genes in 53BP1-S25D versus WT cortical organoids.
(E) Downregulated GSEA terms between 53BP1-S25A versus WT and 53BP1-S25D versus WT were not highly overlapped. Ten GSEA terms were specific to 53BP1-S25D versus WT.
Underlying numerical values for figures are found in S1_Data.xlsx.
