# Supplementary material for: Phosphorylation of the DNA damage repair factor 53BP1 by ATM kinase controls neurodevelopmental programs in cortical brain organoids
Source: PLoS Biol. 2024 Sep 3;22(9):e3002760. doi: 10.1371/journal.pbio.3002760 (PMC11398655; doi:10.1371/journal.pbio.3002760)
Supplement: S12 Fig — (A) Immunofluorescence of NPC markers PAX6 and NESTIN. Bar, 50 μm. GSEA identified top enrichment of differentially expressed genes in (B, D) 53BP1-S25A or (C, E) S25D versus WT NPCs. % Match, % of genes in the enriched term that overlap the differentially expressed genes or proteins. Venn diagrams depict overlaps between down-regulated genes in ATM-KO with 53BP1- (F) S25A or (G) S25D cortical organoids. Underlying numerical values for figures are found in S1 Data. ATM, ataxia telangiectasia mutated; GSEA, gene set enrichment analysis; KO, knockout; NES, normalized enrichment score; NPC, neural progenitor cell; WT, wild type. (PDF) [file pbio.3002760.s014.pdf]

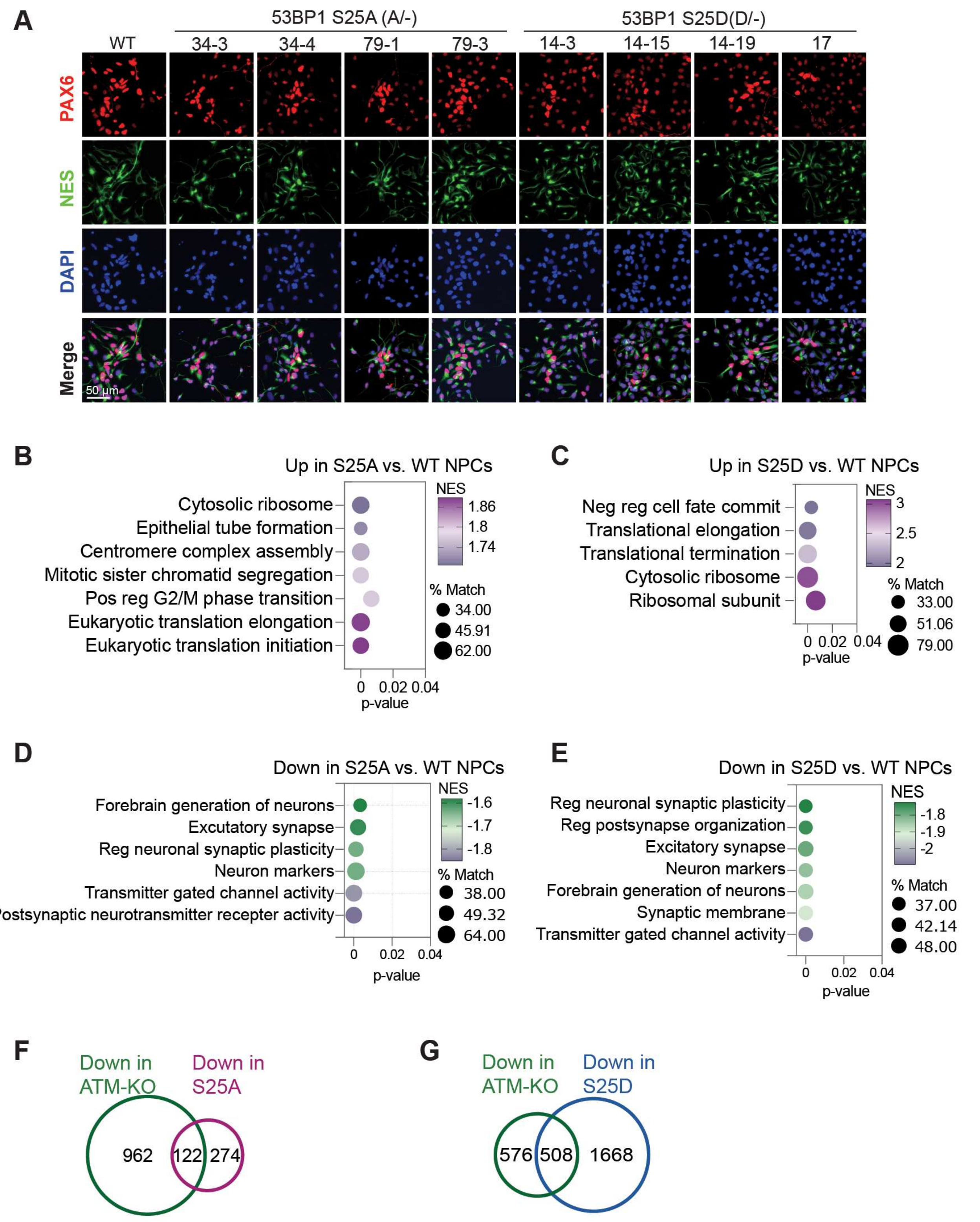

S12 Fig. Characterization of NPCs and comparative analyses of RNA-seq data.
(A) Immunofluorescence of NPC markers PAX6 and NESTIN. Bar, 50 μm.
GSEA identified top enrichment of differentially expressed genes in (B, D) 53BP1-S25A or (C, E) S25D versus WT NPCs. NES, normalized enrichment score. % Match, % of genes in the enriched term that overlap the differentially expressed genes or proteins.

Venn diagrams depict overlaps between downregulated genes in *ATM*-KO with 53BP1- (F) S25A or (G) S25D cortical organoids.
