# Supplementary material for: Phosphorylation of the DNA damage repair factor 53BP1 by ATM kinase controls neurodevelopmental programs in cortical brain organoids
Source: PLoS Biol. 2024 Sep 3;22(9):e3002760. doi: 10.1371/journal.pbio.3002760 (PMC11398655; doi:10.1371/journal.pbio.3002760)
Supplement: S13 Fig — (A) GSEA graphs showed that up-regulated genes in 53BP1-S25A or S25D vs. WT had significant enrichment in down-regulated genes of ATM-KO vs. WT cortical organoids. P values were calculated by the hypergeometric test, assuming normal data distribution. (B) Concordantly differential expression of genes in 53BP1-S25D vs. WT were enriched in those in 53BP1-S25A vs. WT. (C) Concordantly differential expression of genes in 53BP1-S25A vs. WT were enriched in those in 53BP1-S25D vs. WT. For (A-C), P values were calculated by the hypergeometric test, assuming normal data distribution. (D) Proportions of 53BP1 binding to genomic features. 53BP1 ChIP-seq tracks at loci of representative (E) up-regulated and (F) down-regulated genes in 53BP1-S25A and S25D versus WT D35 cortical organoids. (G) S25A and S25D down-regulate 53BP1 targets that are enriched in IRE1-mediated unfolded protein response, regulation of cellular response to stress, iron import into cells, and regulation of apoptosis. Underlying numerical values for figures are found in S1 Data. ATM, ataxia telangiectasia mutated; GSEA, gene set enrichment analysis; KO, knockout; WT, wild type. (PDF) [file pbio.3002760.s015.pdf]

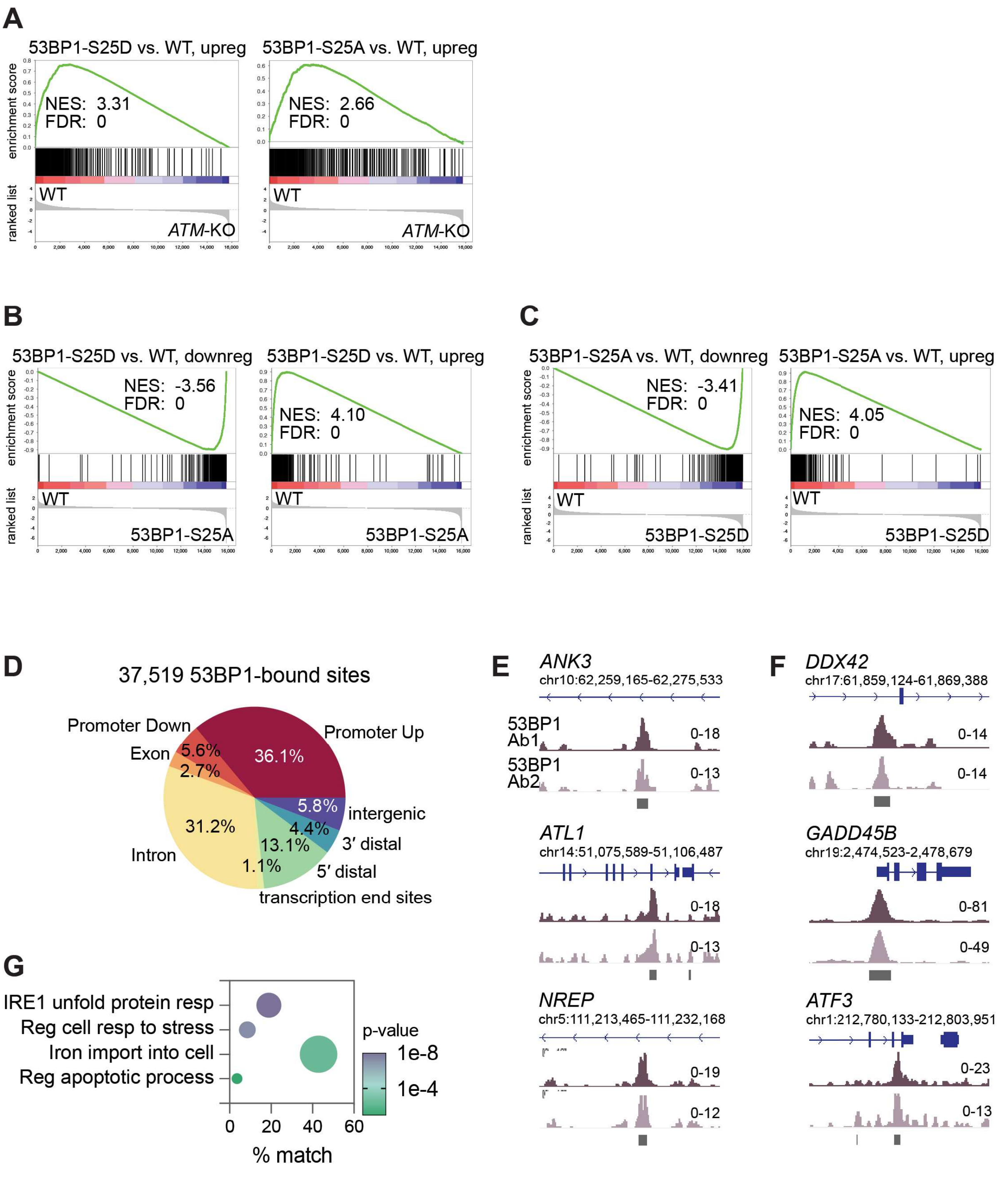

S13 Fig. Comparisons of RNA-seq data and 53BP1 ChIP-seq analyses.

(A) GSEA graphs showed that upregulated genes in 53BP1-S25A or S25D vs. WT had significant enrichment in downregulated genes of ATM-KO vs. WT cortical organoids. *P* values were calculated by the hypergeometric test, assuming normal data distribution.

(B) Concordantly differential expression of genes in 53BP1-S25D vs. WT were enriched in those in 53BP1-S25A vs. WT.

(C) Concordantly differential expression of genes in 53BP1-S25A vs. WT were enriched in those in 53BP1-S25D vs. WT.

For (A-C), *P* values were calculated by the hypergeometric test, assuming normal data distribution.

(D) Proportions of 53BP1 binding to genomic features.
53BP1 ChIP-seq tracks at loci of representative (E) upregulated and (F) downregulated genes in 53BP1-S25A and S25D versus WT D35 cortical organoids.
(G) S25A and S25D downregulate 53BP1 targets that are enriched in IRE1-mediated unfolded protein response, regulation of cellular response to stress, iron import into cells, and regulation of apoptosis.
