# Supplementary material for: Phosphorylation of the DNA damage repair factor 53BP1 by ATM kinase controls neurodevelopmental programs in cortical brain organoids
Source: PLoS Biol. 2024 Sep 3;22(9):e3002760. doi: 10.1371/journal.pbio.3002760 (PMC11398655; doi:10.1371/journal.pbio.3002760)
Supplement: S14 Fig — (A) MA plot displays 53BP1 ChIP-seq signals at genomic sites that are significantly different in ATM-KO vs. WT NPCs. Proportions of genomic features and gene ontology of genes with (B) higher or (C) lower 53BP1 binding in ATM-KO vs. WT NPCs. (D) Proportions of 53BP1-pS25 binding to genomic features. (E) GSEA identified top enrichment of genes occupied by 53BP1-pS25 in WT NPCs. % Match, % of genes in the enriched term that overlap the differentially expressed genes or proteins. Underlying numerical values for figures are found in S1 Data. ATM, ataxia telangiectasia mutated; GSEA, gene set enrichment analysis; KO, knockout; NES, normalized enrichment score; NPC, neural progenitor cell; WT, wild type. (PDF) [file pbio.3002760.s016.pdf]

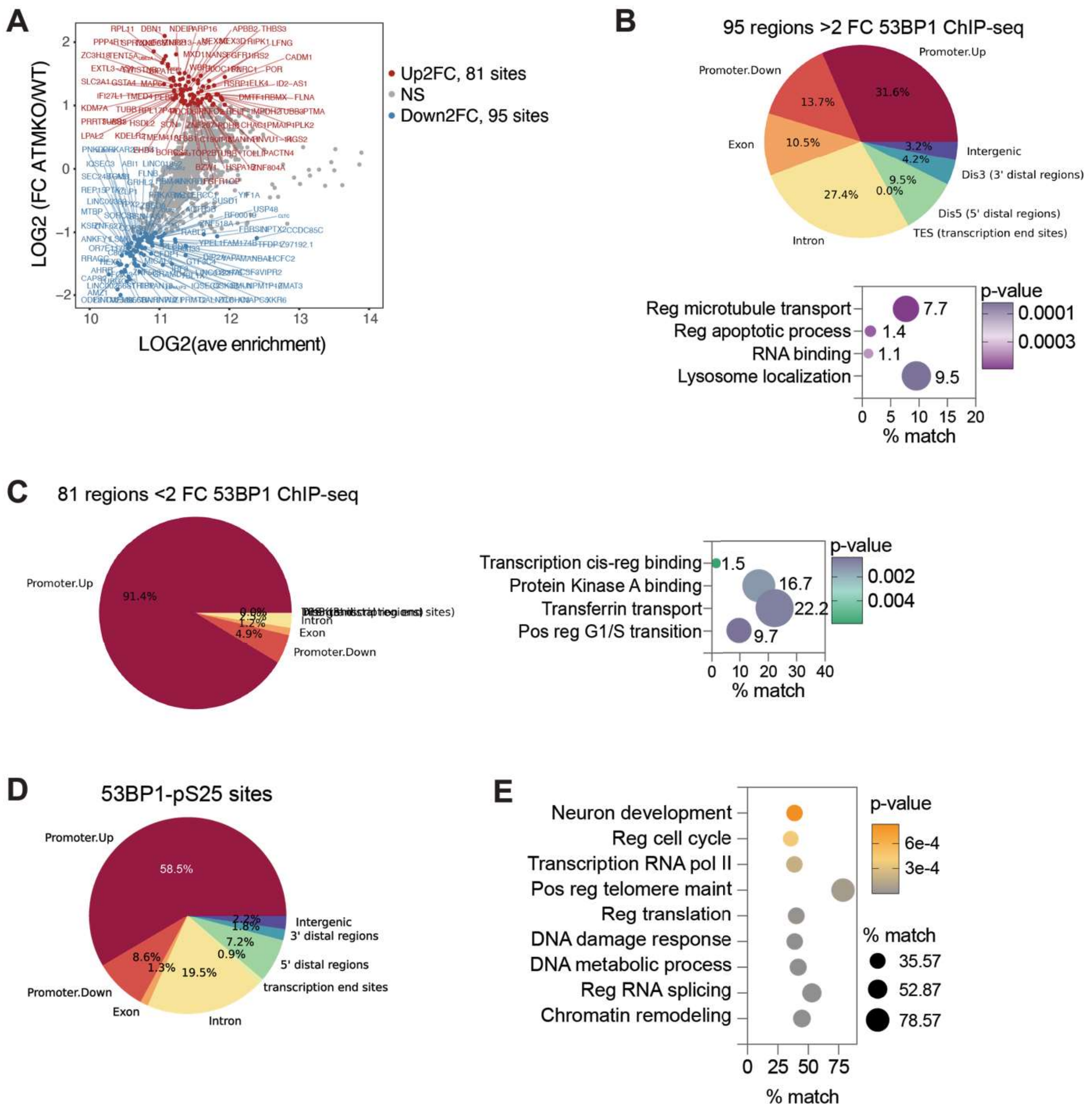

S14 Fig. 53BP1 ChIP-seq and 53BP1-pS25 CUT&RUN.

(A) MA plot displays 53BP1 ChIP-seq signals at genomic sites that are significantly different in *ATM*-KO vs. WT NPCs.

Proportions of genomic features and gene ontology of genes with (B) higher or (C) lower 53BP1 binding in *ATM*-KO vs. WT NPCs.

(D) Proportions of 53BP1-pS25 binding to genomic features.

(E) GSEA identified top enrichment of genes occupied by 53BP1-pS25 in WT NPCs. NES, normalized enrichment score. % Match, % of genes in the enriched term that overlap the differentially expressed genes or proteins.
