# Supplementary material for: Phosphorylation of the DNA damage repair factor 53BP1 by ATM kinase controls neurodevelopmental programs in cortical brain organoids
Source: PLoS Biol. 2024 Sep 3;22(9):e3002760. doi: 10.1371/journal.pbio.3002760 (PMC11398655; doi:10.1371/journal.pbio.3002760)
Supplement: S15 Fig — (A) Principal component analysis of top 3,000 most variable peaks in 53BP1 ChIP-seq of 53BP1-WT, S25A, and S25D NPCs. Two independent cell lines for each group were used for ChIP-seq. Proportions of genomic features in regions with significantly different 53BP1 ChIP-seq in (B) 53BP1-S25A vs. WT and (C) 53BP1-S25D vs. WT, using the criterion of FC>2 and p < 0.05. (D) Heatmaps aligning peaks with significantly different 53BP1 ChIP-seq in 53BP1-S25A vs. S25D. Control regions are those, after voom normalization, showed the least changes and served as semi-independent validation of differential ChIP-seq analysis. Bubble graphs present top enriched categories of genes that had significantly lower 53BP1 ChIP-seq in (E) 53BP1-S25A vs. WT and (F) 53BP1-S25D vs. WT. Underlying numerical values for figures are found in S1 Data. FC, fold-change; NPC, neural progenitor cell; WT, wild type. (PDF) [file pbio.3002760.s017.pdf]

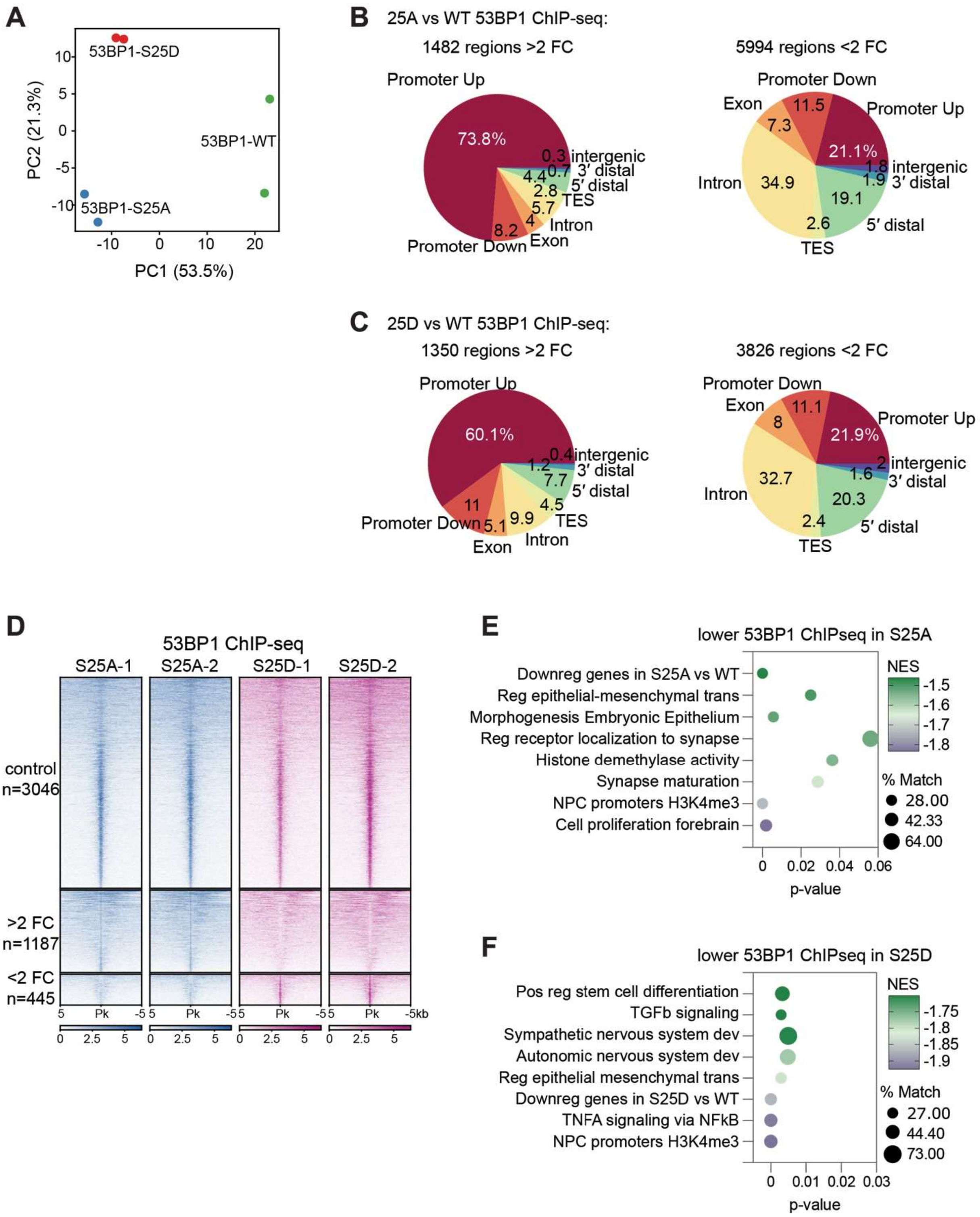

S15 Fig. Differential 53BP1 ChIP-seq in 53BP1-WT, S25A, and S25D NPCs.

(A) Principal component analysis of top 3000 most variable peaks in 53BP1 ChIP-seq of 53BP1-WT, S25A, and S25D NPCs. Two independent cell lines for each group were used for ChIP-seq. Proportions of genomic features in regions with significantly different 53BP1 ChIP-seq in (B) 53BP1-S25A vs. WT and (C) 53BP1-S25D vs. WT, using the criterion of fold-change (FC)>2 and p<0.05.

(D) Heatmaps aligning peaks with significantly different 53BP1 ChIP-seq in 53BP1-S25A vs. S25D. Control regions are those, after voom normalization, showed the least changes and served as semi-independent validation of differential ChIP-seq analysis.

Bubble graphs present top enriched categories of genes that had significantly lower 53BP1 ChIP-seq in (E) 53BP1-S25A vs. WT and (F) 53BP1-S25D vs. WT.
