# Supplementary material for: Phosphorylation of the DNA damage repair factor 53BP1 by ATM kinase controls neurodevelopmental programs in cortical brain organoids
Source: PLoS Biol. 2024 Sep 3;22(9):e3002760. doi: 10.1371/journal.pbio.3002760 (PMC11398655; doi:10.1371/journal.pbio.3002760)
Supplement: S16 Fig — (A) Schematic diagram of neural specification of hESCs with HH (SB421542), TGFβ (dorsomorphin), and WNT (IWR1e and cyclopamine) signaling inhibitors. Nuclear extract was harvested on day 4 and day 10. (B) WB analysis of day 4 samples. (C) Quantification of day 4 WB. Data are presented as the mean ± SEM, and Student t test was performed for pairwise comparisons. n.s., *, and ** indicate not significant, p < 0.05, and p < 0.01, respectively. (D) WB analysis of day 10 samples. (E) Quantification of day 10 WB. Data are presented as the mean ± SEM, and Student t test was performed for pairwise comparisons. n.s., *, and ** indicate not significant, p < 0.05, and p < 0.01, respectively. Underlying numerical values for figures are found in S1 Data. ATM, ataxia telangiectasia mutated; hESC, human embryonic stem cell; WB, western blot. (PDF) [file pbio.3002760.s018.pdf]

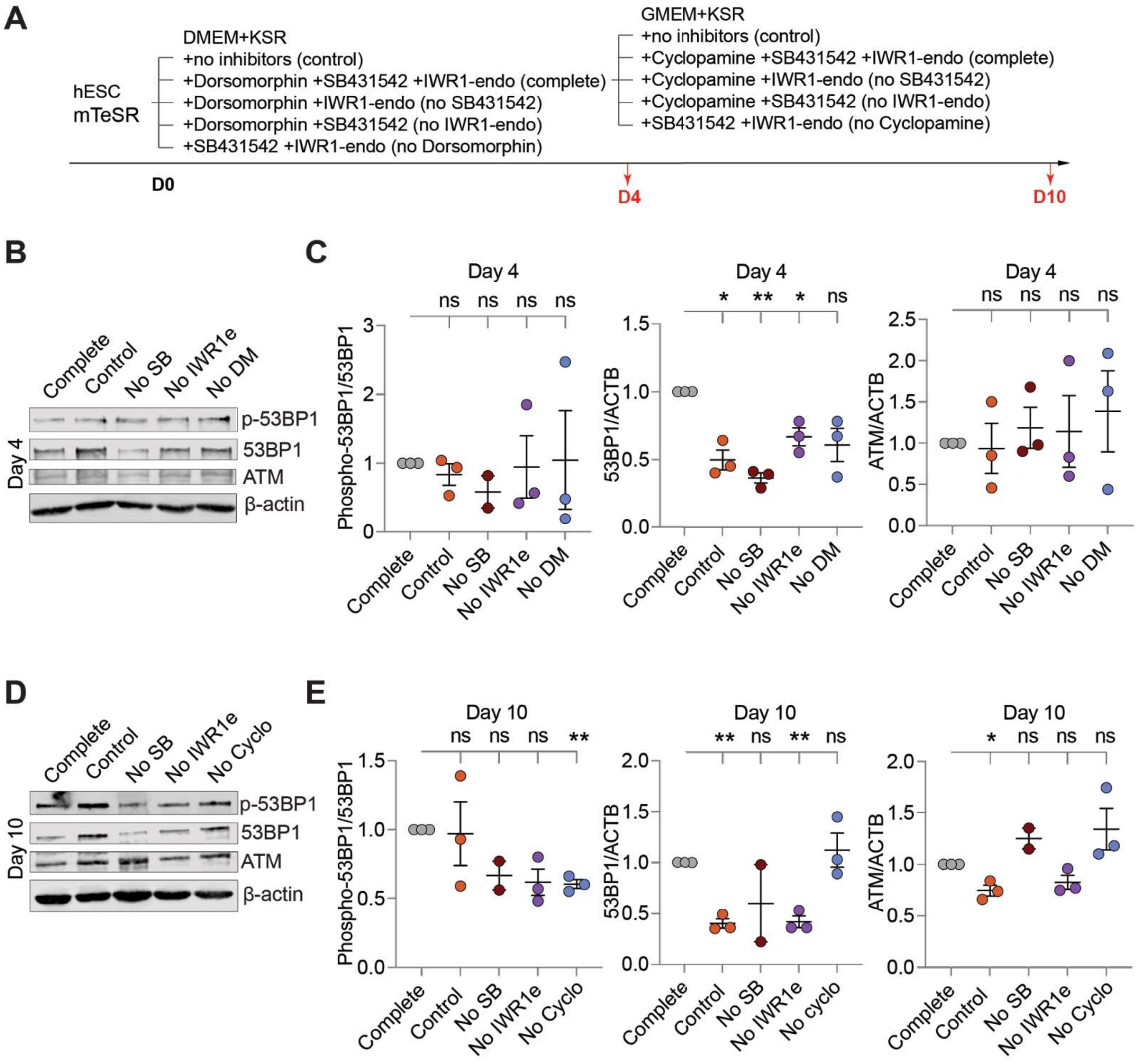

S16 Fig. Analysis of ATM activities during the inhibition of TGFβ, WNT, and HH signaling.

(A) Schematic diagram of neural specification of hESCs with HH (SB421542), TGFβ (dorsomorphin) and WNT (IWR1e & cyclopamine) signaling inhibitors. Nuclear extract was harvested on Day 4 and Day 10.

(B) WB analysis of Day 4 samples.

(C) Quantification of Day 4 WB. Data are presented as the mean ± SEM and Student's t test was performed for pairwise comparisons. n.s., *, and ** indicate not significant, $p<0.05$, and $p<0.01$, respectively.

(D) WB analysis of Day 10 samples.

(E) Quantification of Day 10 WB. Data are presented as the mean ± SEM and Student's t test was performed for pairwise comparisons. n.s., *, and ** indicate not significant, $p<0.05$, and $p<0.01$, respectively.

Underlying numerical values for figures are found in S1_Data.xlsx.
