# Supplementary material for: Phosphorylation of the DNA damage repair factor 53BP1 by ATM kinase controls neurodevelopmental programs in cortical brain organoids
Source: PLoS Biol. 2024 Sep 3;22(9):e3002760. doi: 10.1371/journal.pbio.3002760 (PMC11398655; doi:10.1371/journal.pbio.3002760)
Supplement: S17 Fig — (A) Alignment of WT and RNF168-KO mutation sequences in the RNF168 locus. Red indicates the gRNA sequences. (B) WB analysis of WT and RNF168-KO hESCs. (C) RT-qPCR analysis showing that pluripotent genes in RNF168-KO were expressed higher or the same as those in WT. RNF168-KO did not reduce pluripotent gene expression. *, p < 0.05; ns, not significant by two-way ANOVA text. (D) Immunofluorescence showed similar expression of OCT4 and SSEA4 proteins in WT and RNF168-KO hESCs. Bar, 100 μm. (E) Immunofluorescence of showed similar expression of PAX6 and NES in NPCs. WT and RNF168-KO NPCs. Bar, 50 μm. Functional terms that are highly enriched in (F) up-regulated and (G) down-regulated genes in RNF168-KO D35 cortical organoids. % Match, % of genes in the enriched term that overlap the differentially expressed genes or proteins. (H) Heatmaps aligning peaks with 53BP1-pS25 CUT&RUN signals that were gained, the same, or lost in RNF168-KO vs. WT NPCs, using the criterion of FC>2 and p < 0.05. n = numbers of peaks. Regions with the same signals, are n = 899, which showed the least changes after voom normalization and served as semi-independent validation of differential ChIP-seq analysis. (I) Functional terms of 53BP1-pS25-bound genes in WT NPCs. % Match, % of genes in the enriched term that overlap the differentially bound genes. (J) Number of differentially expressed genes identified by comparison of RNF168-KO vs. WT NPCs at p < 0.05. Of these genes, we list the numbers of 53BP1-pS25-bound targets and targets with higher or lower 53BP1-pS25 CUT&RUN signals in RNF168-KO NPCs. Underlying numerical values for figures are found in S1 Data. FC, fold-change; hESC, human embryonic stem cell; KO, knockout; NES, normalized enrichment score; NPC, neural progenitor cell; RT-qPCR, quantitative reverse transcription PCR; WB, western blot; WT, wild type; 53BP1-pS25, 53BP1 phosphorylated at serine 25. (PDF) [file pbio.3002760.s019.pdf]

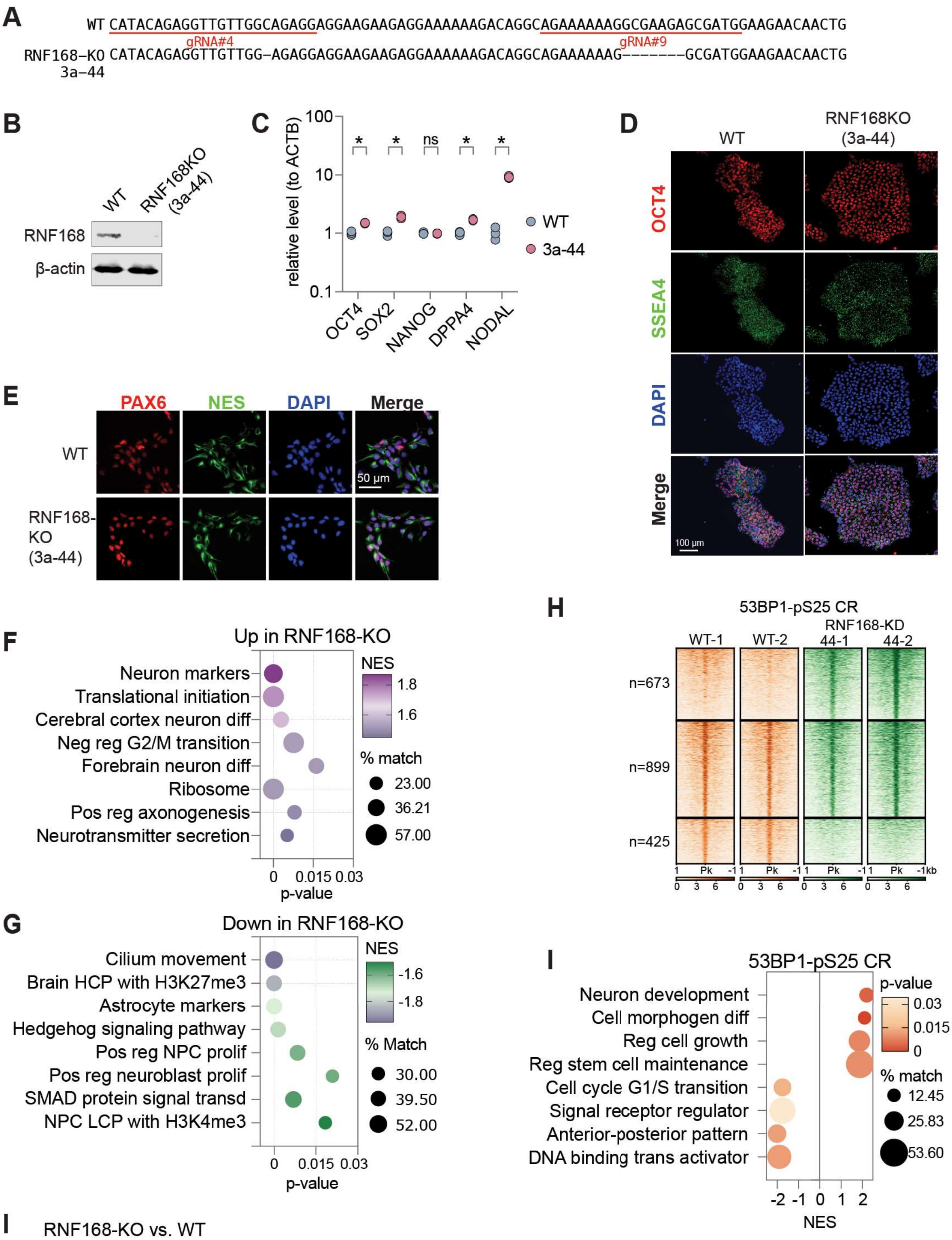

I RNF168-KO vs. WT

	DEG	targets	higher	lower CR
Up	838	187	59	27
Down	923	171	17	57

p<0.05

S17 Fig. *RNF168*-KO alters key genetic programs and 53BP1-pS25 binding to chromatin.

(A) Alignment of WT and *RNF168*-KO mutation sequences in the *RNF168* locus. Red indicates the gRNA sequences.
(B) WB analysis of WT and *RNF168*-KO hESCs.
(C) RT-qPCR analysis showing that pluripotent genes in *RNF168*-KO were expressed higher or the same as those in WT. *RNF168*-KO did not reduce pluripotent gene expression. *, p<0.05; ns, not significant by Two-way ANOVA text.
(D) Immunofluorescence showed similar expression of OCT4 and SSEA4 proteins in WT and *RNF168*-KO hESCs. Bar, 100 μm.
(E) Immunofluorescence of showed similar expression of PAX6 and NES in NPCs. WT and *RNF168*-KO NPCs. Bar, 50 μm.
Functional terms that are highly enriched in (F) upregulated and (G) downregulated genes in *RNF168*-KO D35 cortical organoids. NES, normalized enrichment score. % Match, % of genes in the enriched term that overlap the differentially expressed genes or proteins.
(H) Heatmaps aligning peaks with 53BP1-pS25 CUT&RUN signals that were gained, the same, or lost in *RNF168*-KO vs. WT NPCs, using the criterion of fold-change (FC)>2 and p<0.05. n= numbers of peaks. Regions with the same signals, are n=899, which showed the least changes after voom normalization and served as semi-independent validation of differential ChIP-seq analysis.
(I) Functional terms of 53BP1-pS25-bound genes in WT NPCs. NES, normalized enrichment score. % Match, % of genes in the enriched term that overlap the differentially bound genes.
(J) Number of differentially expressed genes identified by comparison of *RNF168*-KO vs. WT NPCs at p <0.05. Of these genes, we list the numbers of 53BP1-pS25-bound targets and targets with higher or lower 53BP1-pS25 CUT&RUN signals in *RNF168*-KO NPCs.
Underlying numerical values for figures are found in S1_Data.xlsx.
