# Supplementary material for: Phosphorylation of the DNA damage repair factor 53BP1 by ATM kinase controls neurodevelopmental programs in cortical brain organoids
Source: PLoS Biol. 2024 Sep 3;22(9):e3002760. doi: 10.1371/journal.pbio.3002760 (PMC11398655; doi:10.1371/journal.pbio.3002760)

Supplementary Table 1

Cell line	# cells analyzed	Karyotype normal	Karyotype abnormal
**ATM-KO 2**	30	97% 46,XX	3% 46,XX,del(11-q13)
**ATM-KO 3**	40	75% 46,XX	25% 47,XX,+8
**ATM-KO 14**	20	100% 46,XX	0%
**ATM-KO 43**	30	97% 46,XX	3% 46,XX,del(11-q21)
**53BP1-S25A 34-3**	20	100% 46, XX	0%
**53BP1-S25A 34-4**	20	100% 46, XX	0%
**53BP1-S25A 79-1**	20	100% 46, XX	0%
**53BP1-S25A 79-3**	20	100% 46, XX	0%
**53BP1-S25D 14-3**	20	100% 46, XX	0%
**53BP1-S25D 14-15**	20	100% 46, XX	0%
**53BP1-S25D 14-19**	20	100% 46, XX	0%
**53BP1-S25D 17**	20	100% 46, XX	0%
**RNF168-KO 3a-44**	20	100% 46, XX	0%

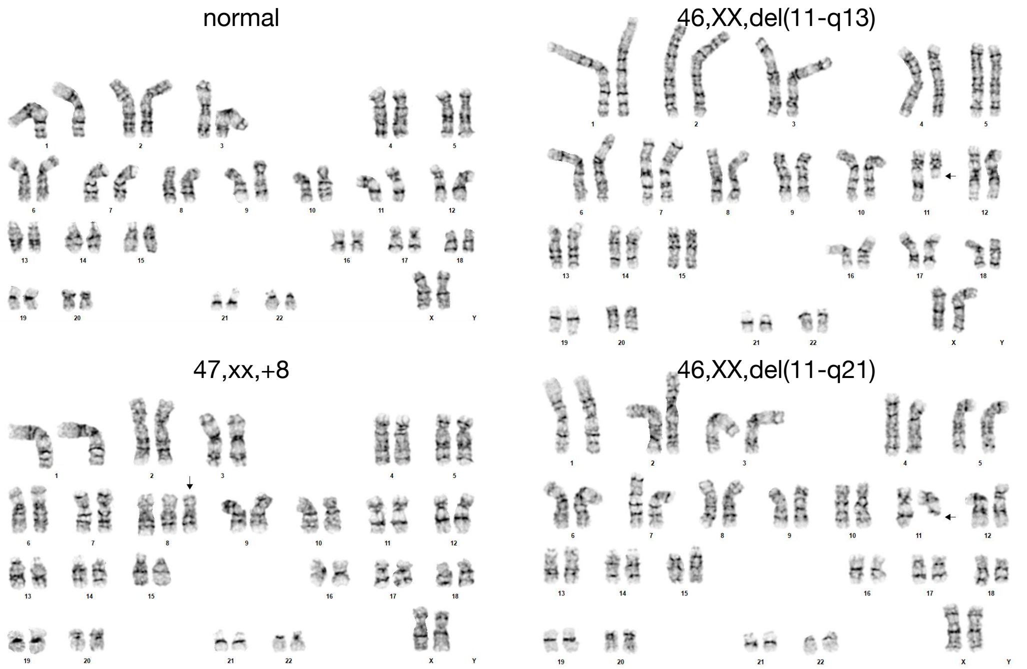

Supplement: S1 Table — Typically normal karyotypes and 3 abnormalities are shown. (PDF) [file pbio.3002760.s020.pdf]
