# Supplementary material for: Phosphorylation of the DNA damage repair factor 53BP1 by ATM kinase controls neurodevelopmental programs in cortical brain organoids
Source: PLoS Biol. 2024 Sep 3;22(9):e3002760. doi: 10.1371/journal.pbio.3002760 (PMC11398655; doi:10.1371/journal.pbio.3002760)
Supplement: S2 Table — Data suggest that D35 ATM-KO cortical organoids specified to the forebrain lineage. (PDF) [file pbio.3002760.s021.pdf]

Transcripts Per Million (TPM) values of each gene were listed.

	geneSymbol	***WT-1***	***WT-2***	***WT-3***	***WT-4***	***WT-5***	***WT-6***
Forebrain	PAX6	44.36	25.47	25.3	21.94	23.59	21.4
	LHX6	0.14	0.04	1.39	0.53	0.39	0.03
	DLX2	0.09	0.16	1.56	1.08	0.51	0.09
	SIX3	0.34	0.18	0.37	0.46	0.13	0.41
	FOXG1	21.48	12.46	16.2	12.89	16.58	13.21
	EMX1	7.15	6.25	5.08	4.31	5.96	7.78
	OTX2	16.69	11.5	3.36	10.55	4.67	29.26
	ZIC1	10.13	5.76	10.89	13.73	4.21	5.3
Midbrain	LMX1A	2.79	1.97	0.77	1.69	1.19	5.33
	LMX1B	0.08	0.06	0.14	0.15	0.16	0.05
	FOXA2	0.02	0.01	0.3	0.19	0.3	0
	TH	0.16	0.13	0.44	0.23	0.1	0.1
	LRRK2	0.2	0.07	0.26	0.23	0.27	0.09
	PRKN	1.15	1.52	1.39	0.99	2.17	0.67
	FOXA1	0.13	0.05	0.45	0.45	0.4	0
	EN1	0.1	0.08	0.06	0.11	0.42	0.13
	EN2	0.11	0.07	0.19	0.28	0.15	0.03
	LMX1B	0.08	0.06	0.14	0.15	0.16	0.05
	PITX3	0	0.01	0	0.05	0.09	0.02
	NR4A2	0.82	0.53	1.34	0.71	1.23	1.62
Hindbrain	EN1	0.1	0.08	0.06	0.11	0.42	0.13
	EN2	0.11	0.07	0.19	0.28	0.15	0.03
	GBX2	0.48	0.21	0.7	0.83	0.43	0.59
	HOXB4	0.13	0.02	0.08	0.35	1.07	0.06
	HOXB8	0.04	0.04	0.04	0.27	0.76	0.01
	HOXB9	0.07	0.02	0.03	0.46	0.57	0.04
	HOXA2	0.19	0.03	1.09	0.21	0.16	0.08
	GATA3	0.03	0.01	0.17	0.17	0.35	0.02
	FOXA2	0.02	0.01	0.3	0.19	0.3	0
	EGR2	0.22	0.09	0.63	0.15	0.26	0.09
	HOXB2	0.22	0	0.68	0.6	1.63	0.18
	FGF8	0.03	0.06	0.24	1.66	0.06	0

ttest vs. Ctrl		***ATM-KO***	***53BP1-S25A***	***53BP1-S25D***
Forebrain	PAX6	0.075	0.331	0.021
	LHX6	0.257	0.363	0.493
	DLX2	0.027	0.185	0.001
	SIX3	0.025	0.172	0.004
	FOXG1	0.463	0.048	0.031
	EMX1	0.127	0.756	0.104
	OTX2	0.313	0.750	0.108
	ZIC1	0.101	0.187	0.761
Midbrain	LMX1A	0.196	0.842	0.135
	LMX1B	0.387	0.516	0.094
	FOXA2	0.708	0.200	0.430
	TH	0.110	0.549	0.091
	LRRK2	0.272	0.358	0.984
	PRKN	0.085	0.477	0.604
	FOXA1	0.582	0.163	0.240
	EN1	0.283	0.688	0.538
	EN2	0.599	0.183	0.508
	LMX1B	0.387	0.516	0.094
	PITX3	0.221	0.177	0.305
	NR4A2	0.032	0.975	0.091
Hindbrain	EN1	0.283	0.688	0.538
	EN2	0.599	0.183	0.508
	GBX2	0.623	0.341	0.066
	HOXB4	0.618	0.305	0.373
	HOXB8	0.467	0.302	0.323
	HOXB9	0.215	0.197	0.177
	HOXA2	0.717	0.875	0.800
	GATA3	1.000	0.241	0.620
	FOXA2	0.708	0.200	0.430
	EGR2	0.567	0.267	0.689
	HOXB2	0.178	0.237	0.172
	FGF8	0.394	0.408	0.544

Student's t test was performed with assumption of unequal variance.

ATM-KO2_rep1	ATM-KO2_rep2	ATM-KO3_rep1	ATM-KO3_rep2	ATM-KO14_rep1	ATM-KO14_rep2	ATM-KO43_rep1	ATM-KO43_rep2
22.65	37.66	45.31	22.02	39.3	50.17	44.65	33.67
0.19	0.62	1	0.67	2.51	0.26	1.33	0.12
5.01	7.74	1.48	1.04	8.96	2.55	2.33	0.56
2.55	3.75	0.86	0.54	2.2	0.97	0.87	0.38
13.41	19.22	20.81	9	22.55	19.2	22.09	11.46
0.6	0.76	7.76	2.25	1.77	6.23	8.77	3.91
3.9	4.6	14.32	3.64	5.05	5.34	17.44	9.87
13.77	16.87	9.18	8.76	11.69	8.18	19.92	9.24
0.23	0.24	2.68	0.52	0.71	0.64	2.9	1.7
0.17	0.28	0.19	0.07	0.19	0.09	0.1	0.02
0.08	0.26	0.16	0.07	0.57	0.04	0.17	0.01
0.42	0.8	0.4	0.13	0.75	0.23	0.23	0.1
0.18	0.22	0.53	0.12	0.81	0.13	0.32	0.08
0.51	1.56	1	0.77	0.85	0.42	1.08	0.45
0.22	0.08	0.13	0.26	0.47	0.05	0.3	0
0.44	0.44	0.1	0.19	0.43	0.1	0.23	0.01
0.05	0.34	0.26	0.03	0.28	0.08	0.31	0.02
0.17	0.28	0.19	0.07	0.19	0.09	0.1	0.02
0	0	0	0	0.03	0	0.03	0
0.1	0.15	0.7	0.27	0.25	0.44	1.44	0.47
0.44	0.44	0.1	0.19	0.43	0.1	0.23	0.01
0.05	0.34	0.26	0.03	0.28	0.08	0.31	0.02
0.2	0.19	0.41	0.38	1.33	0.2	0.84	0.06
0.04	0.4	0.34	0.09	0.39	0.04	0.23	0.02
0.06	0.1	0.07	0.05	0.33	0.11	0.02	0.03
0.09	0.04	0.13	0.04	0.05	0.03	0.04	0.01
0.6	0.85	0.4	0.13	0.34	0.19	0.39	0.01
0.22	0.19	0.3	0.03	0.1	0.06	0.08	0.02
0.08	0.26	0.16	0.07	0.57	0.04	0.17	0.01
0.18	0.17	0.37	0.11	0.17	0.07	0.37	0.03
0.08	0.75	0.05	0.08	0.13	0.11	0.12	0
0.51	0.27	1.11	1.35	0.51	0.05	0.62	0.51

53BP1-S25A-34-3_rep1	53BP1-S25A-34-3_rep2	53BP1-S25A-34-4_rep1	53BP1-S25A-34-4_rep2	53BP1-S25A-79-1_rep1	53BP1-S25A-79-1_rep2	53BP1-S25A-79-3_rep1	53BP1-S25A-79-3_rep2
46.12	29.72	35.59	19.05	38.69	22.05	44.1	22.31
0.23	0.15	0.39	0.29	0.37	0.02	0.12	0.08
3.38	2.58	11.64	2.53	0.47	0.04	0.22	0.08
1.2	0.85	3.15	0.43	0.42	0.27	0.39	0.1
29.01	20.66	31.37	10.25	31.14	15.91	39.54	15.79
9.75	6.13	0.42	3.33	6.51	6.4	13.46	6.48
7.24	8.43	5.35	3.35	6.94	24.88	23.77	8.65
8.42	5.5	6.37	10.41	3.26	3.11	6.21	2.45
1.63	1.34	0.3	0.98	1.5	4.7	5.19	1.17
0.13	0.07	0.44	0.31	0.07	0.02	0.09	0.03
0.12	0.01	0.13	0.01	0.02	0	0.07	0.02
0.73	0.22	0.36	0.25	0.22	0.08	0.07	0.08
0.13	0.09	0.22	0.09	0.19	0.17	0.2	0.09
0.75	0.79	1.8	0.87	1.16	0.65	2.16	0.62
0.14	0.08	0.08	0.06	0.25	0.08	0.09	0.06
0.37	0.13	0.22	0.04	0.57	0	0.11	0.05
0.09	0.01	0.13	0.15	0.09	0.05	0.09	0.02
0.13	0.07	0.44	0.31	0.07	0.02	0.09	0.03
0	0	0	0	0	0	0.04	0
0.97	0.84	0.16	0.4	1.49	1.35	2.22	0.83
0.37	0.13	0.22	0.04	0.57	0	0.11	0.05
0.09	0.01	0.13	0.15	0.09	0.05	0.09	0.02
0.56	0.46	0.35	1.12	0.56	0.81	1.63	0.29
0.15	0.03	0.11	0.04	0.2	0.01	0.19	0.04
0.1	0.02	0.04	0.07	0.03	0.04	0.12	0.02
0.01	0.05	0.06	0	0.05	0	0.18	0.02
0.31	0.2	0.51	0.51	0.25	0.05	0.23	0.06
0.05	0.02	0.16	0.02	0.05	0.01	0.06	0.04
0.12	0.01	0.13	0.01	0.02	0	0.07	0.02
0.11	0.11	0.13	0.25	0.14	0.06	0.14	0.14
0.19	0.31	0.55	0.35	0.26	0	0.1	0.02
0.13	0.18	0.16	0.09	0.14	0.03	0.08	0

53BP1-S25D-14-3_rep1	53BP1-S25D-14-3_rep2	53BP1-S25D-14-15_rep1	53BP1-S25D-14-15_rep2	53BP1-S25D-14-19_rep1	53BP1-S25D-14-19_rep2	53BP1-S25D-17_rep1	53BP1-S25D-17_rep2
37.9	37.13	43.71	36.24	45.14	34.03	29.48	45.25
0.14	0.73	2.39	0.27	0.3	0.31	0.9	0.23
3.02	6.94	4.32	0.22	5.23	4.81	3.73	4.77
1.22	2.72	2.09	0.27	2.23	1.37	0.97	1.01
23.78	23.83	31.59	22.33	26.54	22.94	10.23	15.88
7.3	2.53	1.98	9.33	3.63	3.41	2.07	3.02
3.81	5.59	7.05	4.47	4.76	4.59	5.47	5.06
3.29	5.09	17.37	4.44	6.68	11.41	11.49	12.72
0.73	0.75	0.36	0.85	1.24	0.59	2.35	1.7
0.13	0.48	0.06	0.05	0.17	0.23	1.17	1.15
0.07	0.16	0.09	0.05	0.11	0.06	0.11	0.03
0.11	0.7	0.36	0.15	0.44	0.27	0.3	0.48
0.14	0.11	0.22	0.12	0.21	0.23	0.26	0.21
0.83	1.62	0.84	0.62	1.2	1.25	1.39	1.69
0.04	0.16	0.09	0.06	0.22	0.07	0.17	0.23
0.17	0.23	0.57	0.09	0.46	0.05	0.08	0.01
0.07	0.12	0.06	0.04	0.11	0.17	0.18	0.13
0.13	0.48	0.06	0.05	0.17	0.23	1.17	1.15
0.01	0	0.01	0.01	0.04	0	0	0.02
0.68	0.34	0.95	1.09	0.64	0.53	0.45	0.67
0.17	0.23	0.57	0.09	0.46	0.05	0.08	0.01
0.07	0.12	0.06	0.04	0.11	0.17	0.18	0.13
0.71	0.51	0.73	0.48	2.15	1.01	1.85	0.85
0.07	0.28	0.08	0.03	0.18	0.05	0.22	0.07
0.05	0.05	0.07	0.04	0.03	0.08	0.15	0.02
0	0.04	0.05	0.01	0.05	0.03	0.11	0.02
0.24	0.44	0.11	0.11	0.2	0.24	0.66	0.73
0.04	0.21	0.07	0.03	0.06	0.05	0.22	0.07
0.07	0.16	0.09	0.05	0.11	0.06	0.11	0.03
0.12	0.01	0.15	0.07	0.2	0.34	0.29	0.42
0.17	0.23	0.09	0.04	0.14	0.13	0.36	0.18
0	0.07	0.12	0.14	0.08	0.05	0.28	0.58
