# Supplementary material for: Phosphorylation of the DNA damage repair factor 53BP1 by ATM kinase controls neurodevelopmental programs in cortical brain organoids
Source: PLoS Biol. 2024 Sep 3;22(9):e3002760. doi: 10.1371/journal.pbio.3002760 (PMC11398655; doi:10.1371/journal.pbio.3002760)
Supplement: S5 Table — Data from WT and 53BP1 mutants are compared pairwise by using the two-sample t test. The sizes of organoids are significantly different between each comparison pair (all p < 0.05). (PDF) [file pbio.3002760.s024.pdf]

Two-sample T-test (WT vs. mutants)

| comparison | | t Value | Pr > \|t\| |
|---|---|---|---|
| WT-1 | S25A 34-3 | -10.36 | <.0001 |
| WT-1 | S25A 34-4 | -18.57 | <.0001 |
| WT-1 | S25A 79-1 | -4.24 | <.0001 |
| WT-1 | S25A 79-3 | -3.52 | 0.0004 |
| WT-1 | S25D 14-3 | -16.91 | <.0001 |
| WT-1 | S25D 14-15 | -12.76 | <.0001 |
| WT-1 | S25D 14-19 | -7.63 | <.0001 |
| WT-1 | S25D 17 | -6.35 | <.0001 |
| WT-2 | S25A 34-3 | -13.7 | <.0001 |
| WT-2 | S25A 34-4 | 19.72 | <.0001 |
| WT-2 | S25A 79-1 | -5.96 | <.0001 |
| WT-2 | S25A 79-3 | -5.16 | <.0001 |
| WT-2 | S25D 14-3 | -18.14 | <.0001 |
| WT-2 | S25D 14-15 | -14.46 | <.0001 |
| WT-2 | S25D 14-19 | -9.27 | <.0001 |
| WT-2 | S25D 17 | -8.04 | <.0001 |

**S5 Table.** Two-sample *t*-test examines the sizes of cortical organoids that change between day 35 and day 55 of differentiation. Data from wild-type and 53BP1 mutants are compared pair-wise by using the two-sample t-test. The sizes of organoids are significantly different between each comparison pair (all $p < 0.05$).
