# Supplementary material for: Phosphorylation of the DNA damage repair factor 53BP1 by ATM kinase controls neurodevelopmental programs in cortical brain organoids
Source: PLoS Biol. 2024 Sep 3;22(9):e3002760. doi: 10.1371/journal.pbio.3002760 (PMC11398655; doi:10.1371/journal.pbio.3002760)
Supplement: S6 Table — This table lists the calculation for different combinations of data and the descriptive statistics. (PDF) [file pbio.3002760.s025.pdf]

Frequency table of change values

Type	n pairs (D55-D35)	n organoids@ D35	n organoids@ D55	Mean	STDDEV	Min	Max
WT-1	720	40	18	4978.41	4652.28	-7099	16018
WT-2	585	39	15	5407.41	4475.87	-7464	19093
S25A 34-3	1692	47	36	2993.29	3351.85	-5833	16148
S25A 34-4	1505	43	35	1427.69	3125.62	-7267	9852
S25A 79-1	1204	43	28	4029.41	4813.68	-7445	17566
S25A 79-3	1066	41	26	4157.93	5089.87	-8628	17365
S25D 14-3	1408	44	32	1693.88	3281.67	-8699	10981
S25D 14-15	1232	44	28	2286.89	4212.01	-8321	12011
S25D 14-19	1247	43	29	3422.64	3795.38	-4957	13397
S25D 17	1333	43	31	3577.88	4830.71	-7145	19121

S6 Table.

The changes in organoid size at days 35 and 55 of differentiation were compared to yield Supplementary Figure 3C. This table lists the calculation for different combinations of data and the descriptive statistics.
