# Supplementary material for: Phosphorylation of the DNA damage repair factor 53BP1 by ATM kinase controls neurodevelopmental programs in cortical brain organoids
Source: PLoS Biol. 2024 Sep 3;22(9):e3002760. doi: 10.1371/journal.pbio.3002760 (PMC11398655; doi:10.1371/journal.pbio.3002760)
Supplement: S7 Table — (PDF) [file pbio.3002760.s026.pdf]

S7 Table.

Primary Antibodies	Company	Cat. No.	Experimental Condition
53BP1-pS25	Abcam	ab70323	WB (1:1000)
53BP1	Novus Biological	NB100-304	WB (1:1000)
UTX	Millipore /<br>Cell Signaling Technology	ABE409 /<br>33510	WB (1:1000)<br>WB (1:1000)
PAX6	BioLegend	901301	IF (1:200)
CTIP2	Abcam	Ab18465	IF (1:500)
KI67	Cell Signaling Technology	9129S	IF (1:100)
β-actin	Sigma Aldrich	A1978	WB (1:1000)
ATM	Abcam	Ab17995	WB (1:1000)
SUZ12	Cell signaling Technology	3737S	WB (1:1000)
OCT4	Cell signaling Technology	2840S	IF (1:200)
SSEA4	STEMCELL Technologies	60062AD	IF (1:100)
pS10H3/PH3	Cell Signaling Technology	3465S	IF (1:1000)
ZO-1	Thermo Fisher Scientific	33-9100	IF (1:300)
gH2AX	Millipore	05-636	WB (1:1000)
Histone H3	Rockland Immunochemicals	100-401-E81	WB (1:2000)
CC3	Cell Signaling Technologies	9661S	IF (1:200)
NESTIN	Santa Cruz Biotechnology	SC-23927	IF (1:300)
NEUN	Millipore	ABN90	IF (1:200)
TUJI1	Sigma	T8660	IF (1:300)
