# Supplementary material for: Phosphorylation of the DNA damage repair factor 53BP1 by ATM kinase controls neurodevelopmental programs in cortical brain organoids
Source: PLoS Biol. 2024 Sep 3;22(9):e3002760. doi: 10.1371/journal.pbio.3002760 (PMC11398655; doi:10.1371/journal.pbio.3002760)
Supplement: S8 Table — (PDF) [file pbio.3002760.s027.pdf]

S8 Table.

Name	Sequence (5' to 3')
CAGE299.TP53BP1.g6 spacer	gcugagaaucuucaauuauc
CAGE299.g6.S25A.anti.ssODN (S25A and silent g6-blocking changes in upper case)	gaaccagaatcatcctctagaacctggctttcaggctgagCatcttcGatGatcaggcaaggagtatcttgctgagagaaatctgaatcca
CAGE299.g6.S25D.anti.ssODN (S25D and silent g6-blocking changes in upper case)	gaaccagaatcatcctctagaacctggctttcaggctgaTCatcttcGatGatcaggcaaggagtatcttgctgagagaaatctgaatcca
CAGE299.F	gtcctgaagtggtcccgttt
CAGE299.R	aggtatcactcaccaacacagg
CAGE3031.RNF168.9.g4	catacagaggttgttggcagngg
CAGE3031.RNF168.9.g9	agaaaaaaggcgaagagcgangg
