# Supplementary material for: Phosphorylation of the DNA damage repair factor 53BP1 by ATM kinase controls neurodevelopmental programs in cortical brain organoids
Source: PLoS Biol. 2024 Sep 3;22(9):e3002760. doi: 10.1371/journal.pbio.3002760 (PMC11398655; doi:10.1371/journal.pbio.3002760)
Supplement: Attachment — Submitted filename: response.pdf [file pbio.3002760.s028.pdf]

We appreciate the reviewers' their thoughtful comments, which have significantly improved all sections of this manuscript. We have obtained new data to strengthen the following conclusions.

- Improved characterization of the cellular defects in *ATM*-KO cortical organoids.
- New findings about the temporal progression of *ATM*-KO, 53BP1-S25A, and 53BP1-S25D compared with WT cortical organoids, elucidating the role of ATM and 53BP1 in controlling neuronal differentiation and tissue organization.
- Identification of HH signaling in influencing ATM activities during neural differentiation.
- Identification of RNF168 as an additional player in this pathway, regulating neurodevelopmental genetic programs.

Reviewer #1: This manuscript from Peng and colleagues investigate the role of ATM-dependent 53BP1 ser25 phosphorylation in neural development. Significantly, this work represents the follow up to prior work from the same group (Nature Neuroscience 2019), in which they reported a role for promoter bound 53BP1 - UTX complexes in upregulating neurodevelopmental genes during the differentiation of human embryonic stem cells into neurons or into cortical organoids.

Here the paper starts with the observation that 53BP1 expression doesn't change during differentiation from hESCs to neurons/cortical organoids, so they speculate it might instead be controlled by 53BP1 phosphorylation ATM, and then concentrate on the well-known Ser25-phosphosite in 53BP1 (a known ATM target) since this modification appears to be reduced in differentiated NPCs relative to hESCs in western blotted lysates. In correlation of such a notion, they find ATM-KO cells also show perturbed/modified transcriptional expression profiles during the differentiation experiments, they show ATM interacts with 53BP1 (by IP), and they show NPCs with 53BP1 Ser25Ala/Asp knockin mutations also have altered expression profiles, including alterations in neural gene expression profiles. Lastly, they correlate these changes to the altered residency of 53BP1 -S25 mutant proteins on specific gene promoters relative to wild type 53BP1 (which over-accumulates relative to WT), on which they speculate that ATM-dependent 53BP1 phosphorylation, guided by upstream DDR signalling incl ATM-dependent gH2AX induction at TSSs which recruits 53BP1, then negatively regulates 53BP1 occupancy on bivalent gene promoters, thereby modulating expression and contributing to neuronal differentiation.

The paper is quite descriptive and correlative in nature, with the bulk of data using transcriptomics and proteomics to identify changes in protein/gene expression between the different genetic backgrounds, GSEA/similar to attribute this to different gene classes that include (but are not limited to) genes involved in neural differentiation. In then showing that differences occur both in ATM and 53BP1 mutant cells, there may be some correlation/overlap, but it is difficult to interpret this as the stated causality. The authors identify interesting differences in S25A/D mutant 53BP1 vs WT 53BP1 promoter binding patterns, but how these alters gene expression patterns is hard to discern from the way the data is presented. I also found the evidence backing for the proposed model (and sequence of events) to be lacking. If ATM indeed regulates 53BP1 promoter binding via pS25 phosphorylation, this could be easily demonstrated by ChIP'ing wild type and mutant p53 in the ATM-KO cells, as one would expect the effect to increase promoter binding in the case of the wild-type but the mutant protein would not be impacted. However, even if this was the case, perhaps an even more important gap in the model is the DNA-damage stimulus for ATM-dependent regulation of 53BP promoter interactions? IT is proposed that DDR signalling via ATM be both positively and negatively regulates 53BP1-promoter interactions,

via gH2AX-dependent recruitment, and Ser-25 phosphorylation of 53BP1, respectively. However, this is a counterintuitive concept to grasp, and since it is not supported by the included evidence, I'm left not really understanding the mechanism or how it might regulate neuronal identify or function.

We appreciate the excellent summation of this manuscript and agree that we had uncovered a noncanonical role of DNA damage signaling, involving ATM and 53BP1, in controlling specific genetic programs in NPCs and cortical organoids. However, the exact mechanistic details of this pathway remain unclear. We value the suggestion to explore the involvement of RNF168 in this pathway as this discovery introduces a new molecular player and provides an additional support for this pathway.

Specific Comments:

- As described above, causality is not established in the case of how wild-type 53BP1 promoter interactions are impacted by ATM status, and to what degree this occurs via Ser-25 phosphorylation, thus this conclusion is not substantiated.

We agree and compared 53BP1 ChIP-seq in WT and *ATM*-KO NPCs. This comparison identified a modest number of changes in 53BP1 binding to chromatin. *ATM*-KO strongly depleted 53BP1-pS25 and not dramatically changed 53BP1 binding to chromatin. We added in lines 328-331, "We wanted to test whether ATM alters 53BP1 binding, considering ATM is required for 53BP1-pS25 in D35 cortical organoids (Fig 1F) and NPCs (Supplementary Fig 2B). A comparison of 53BP1 ChIP-seq in WT and *ATM*-KO NPCs showed that *ATM*-KO altered 53BP1 binding to chromatin (Supplementary Fig 14A-C). *ATM*-KO reduced 53BP1 binding at specific sites, with 96.3% of these sites being promoters (Supplementary Fig 14C)."

We then examined 53BP1-S25 phosphorylation, finding that the antibody specific to 53BP1-pS25 is suitable for CUT&RUN but not for ChIP. To line 332-343, "To explore the impact of 53BP1-pS25, we performed CUT&RUN in 2 separate WT NPC lines. Our analysis revealed that 67.1% of 53BP1-pS25 targets localize to promoter regions, suggesting a transcriptional regulatory role (Supplementary Fig 14D). Under the criteria of fold-change >2 and $p<0.05$, 58.6% (3390/5789) of 53BP1-pS25 targets overlapped with 53BP1 targets (Fig 7C); the non-overlapped sites may be attributed to differences in ChIP versus CUT&RUN procedures and the accessibility of 53BP1 versus 53BP1-pS25 antibodies. 53BP1-pS25 targets were significantly enriched in 414 upregulated genes in NPCs versus ESCs (Fig 7D), suggesting a role of 53BP1-pS25 in promoting their expression in NPCs. Genes having overlapped 53BP1 ChIP-seq and 53BP1-pS25 CUT&RUN signals were enriched in chromatin remodeling, DNA metabolism, RNA splicing, translation, transcription, cell cycle, and neuron development (Supplementary Fig 14E). These data suggest that ATM can alter 53BP1 binding and that 53BP1-pS25 is enriched in the promoters of genes regulating cellular processes and neurodevelopment.

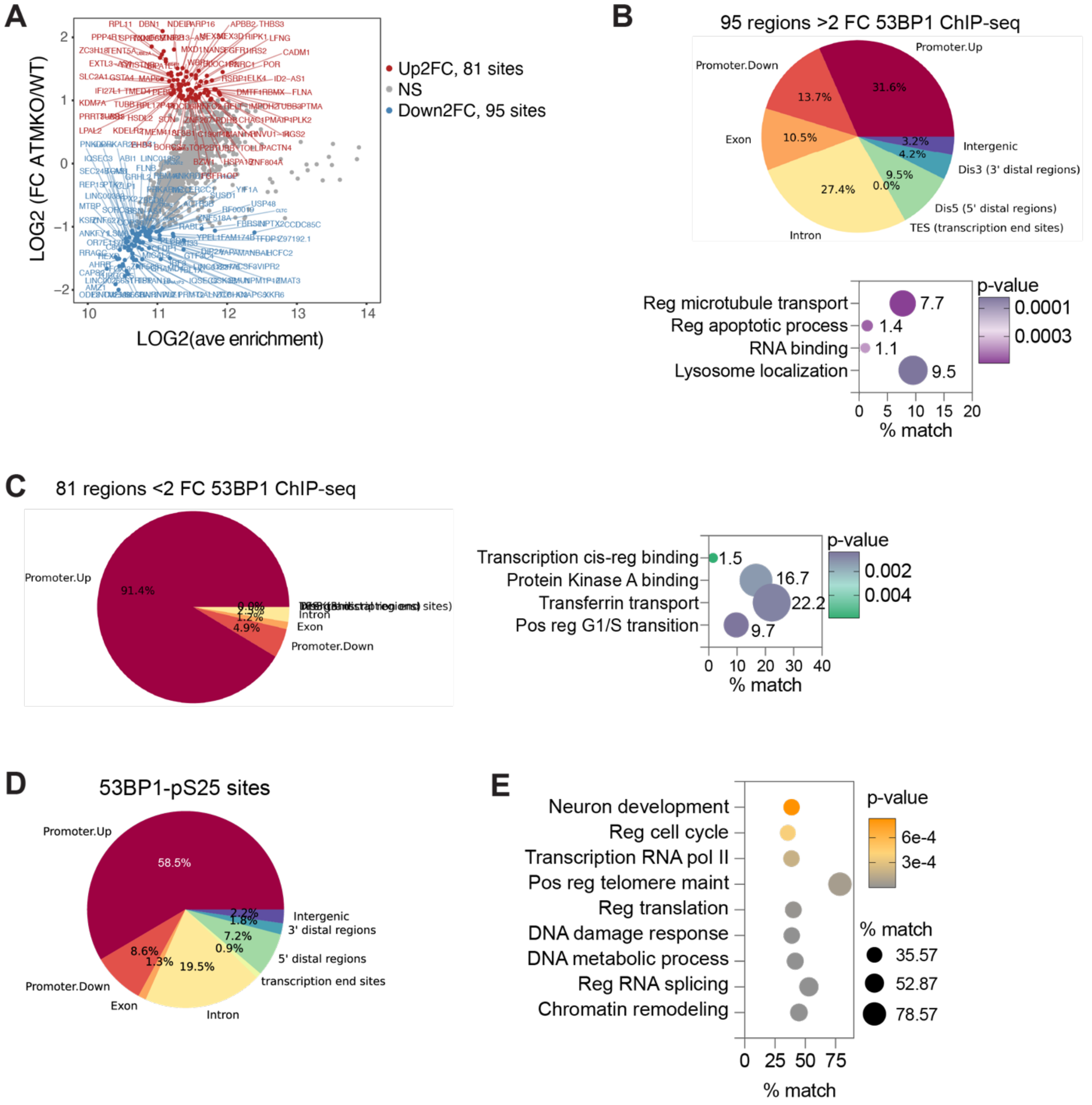

Supplementary Figure 14. 53BP1 ChIP-seq and 53BP1-pS25 CUT&RUN.

(A) MA plot displays 53BP1 ChIP-seq signals at genomic sites that are significantly different in *ATM*-KO vs. WT NPCs.

Proportions of genomic features and enriched pathways of genes with (B) higher or (C) lower 53BP1 binding in *ATM*-KO vs. WT NPCs.

(D) Proportions of genomic features with 53BP1-pS25 binding in NPCs.

(E) Top enriched pathways of genes that had both 53BP1 ChIP-seq and 53BP1-pS25 CUT&RUN signals.

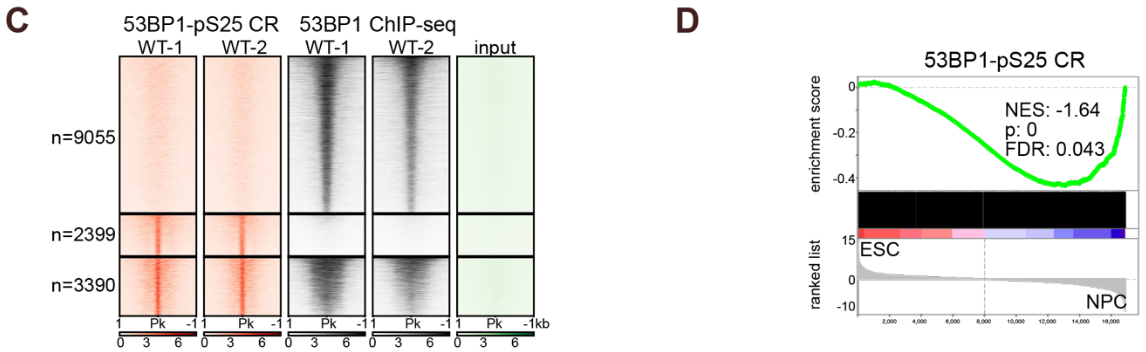

Figure 7.
(C) (7C) Heatmaps aligning peaks with significantly different 53BP1-pS25 CUT&RUN and 53BP1 ChIP-seq signals, using the criteria of FC>2 and $p<0.05$. Chromatin input sample is shown as a negative control. n= numbers of regions with differential and overlapped bindings.
(D) GSEA graph showing enrichment of 53BP1-pS25 CUT&RUN signals in genes that were lower in ESCs vs. NPCs, which were upregulated in NPCs. *P* values were calculated by the hypergeometric test, assuming normal data distribution.

- Earlier work showed this 53BP1-depedentn regulation of neuronal function was not conserved between mouse and man. This manuscript should be transparent in stating this is not a well conserved mechanism.

We appreciate the opportunity to clarify and have attempted to present this poor conservation in a more positive light. Revised line 31-32 now has "Intriguingly, the 53BP1–UTX interaction is observed in humans but not in mice (6); this mechanism is not well conserved and likely regulates primate neurodevelopment." And "in humans but not in mice" was added in line 31.

- If this is all dependent on ATM signalling, what is then stimulating ATM activity, and how its this guiding this activity specifically at promoters?

We agree that this is a very interesting question.

We tried to address this question by manipulating our differentiation system, given the current understanding about regulation of ATM signaling is restricted to DNA damage response and not related to neural differentiation. The data point to HH signaling for ATM regulation. We added to line 392-401, "We next tried to identify a regulation of ATM, whose protein levels increased in NPCs (Fig 1D). This led us to test whether and how inhibitors of TGFβ, WNT, and HH signaling control protein levels of ATM, 53BP1, and pS25-53BP1 by removing one inhibitor at a time from the cortical organoid differentiation media (Supp Fig 16A). As we could not successfully identify physical presence of ATM at promoters, WB analysis is most apt to study ATM level and activity. By day 4 of neural differentiation, although 53BP1 protein levels were reduced by the withdrawal of SB431542 (TGFβ inhibitor) or IWR1-endo (WNT inhibitor), pS25-53BP1 was not altered (Supp Fig 16B-C). By day 10 of neural differentiation, the withdrawal of cyclopamine (HH inhibitor) reduced pS25-53BP1 level (but not ATM or 53BP1 proteins; Supp Fig 16D-E). These data suggest that HH signaling pathway modulates ATM activity during neural differentiation."

To the "Discussion" section, we edited lines 429-440 to "We have shown that neural differentiation promotes ATM protein levels, and ATM-dependent phosphorylation predominantly

impacts factors involved in neurogenesis, neuronal differentiation, cell morphogenesis, and microtubule cytoskeleton. HH signaling likely inhibits S25-53BP1 phosphorylation by ATM, and TGFβ and WNT signaling pathways reduced ATM protein levels. The activity of ATM can be regulated by DNA damage response, reactive oxygen species, hypothxia, hypothermia, and phosphatase WIP1 [31-33]. The exact clarification of mechanisms promoting ATM activities, especially in directing its kinase activity at specific promoters, is beyond the scope of this study. Additionally, we have identified kinases involved in ATM, BNDF, and WNT signaling, G2/M checkpoint, and p53 regulation as being influenced by ATM-dependent phosphorylation during cortical organoid differentiation. These findings have broad implications about diseases associated with ATM, including ataxia telangiectasia [34-36]."

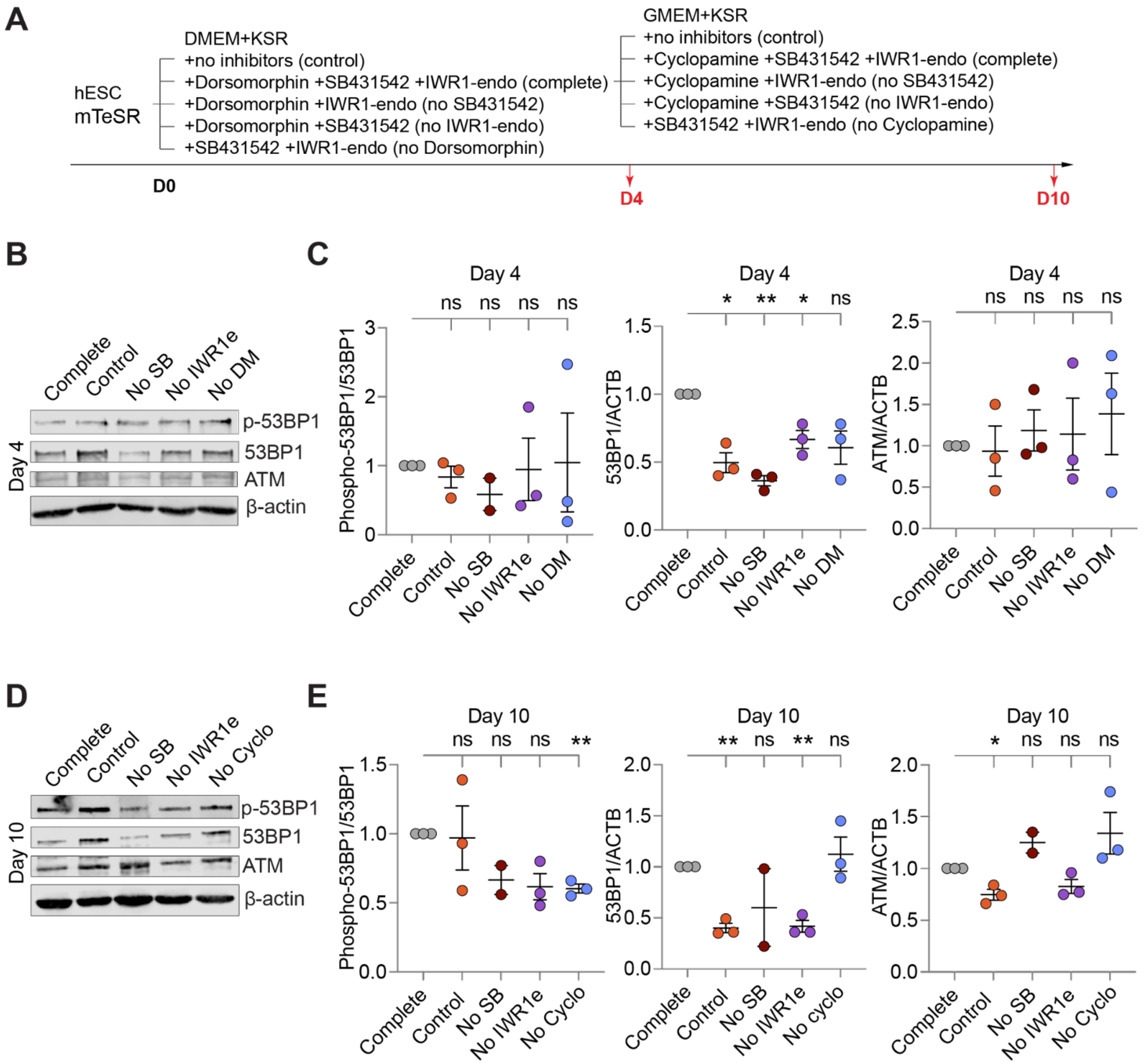

Supplementary Figure 16. **Analysis of ATM activities during the inhibition of TGFβ and WNT signaling.**

(A) Schematic diagram of neural specification of hESCs with TGFb (SB421542 & dorsomorphin) and WNT (IWR1e & cyclopamine) signaling inhibitors. Nuclear extract was harvested on Day 4 and Day 10.

(B) WB analysis of Day 4 samples.
(C) Quantification of Day 4 WB. Data are presented as the mean ± SEM and Student's t test was performed for pairwise comparisons. n.s., *, and ** indicate not significant, p<0.05, and p<0.01, respectively.
(D) WB analysis of Day 10 samples.
(E) Quantification of Day 10 WB. Data are presented as the mean ± SEM and Student's t test was performed for pairwise comparisons. n.s., *, and ** indicate not significant, p<0.05, and p<0.01, respectively.

- The author speculate this might be controlled by gH2AX-dependent recruitment of 53BP1 (presumably to sites of DNA damage). If so, then this would be controlled by gH2AX-MDC1-RNF8-RNF168-H2AK15ub axis which is essential for 53BP1 chromatin binding. RNF168 KO cells would test or refute this hypothesis and such an experiment would be needed to substantiate such a claim.

We designed 2 gRNAs targeting exon 3 and 2 gRNAs targeting exon 4 of the *RNF168* locus to generate *RNF168*-KO. Screening of 384 hESC clones by MiSeq analysis identified several clones with KO mutations in silico; >95% of the clones had in-frame deletions of <10 amino acids. WB analysis revealed that clone 44 had depletion of RNF168. Due to time constraints associated with neural differentiation and subsequent analyses of NPCs for RNA-seq and CUT&RUN, we focused our analyses on clone 44. We recognize that a question might arise about the similarities between *RNF168*-KO and *ATM*-KO. While no downregulated genes were shared, 68 downregulated genes were shared. The top GSEA terms for these shared genes were neuron differentiation and central nervous system development. These data suggest that RNF168 affects 53BP1-pS25 on chromatin and the expression of key genetic programs for neural differentiation.

We added to line 402-420, "Next, we tested whether another DNA damage response factor, apart from ATM, influences 53BP1-pS25. RNF168 plays a central role in the γH2AX-MDC1-RNF8-RNF168-H2AK15ub axis, which governs the binding of 53BP1 to chromatin with DNA damage (19203579). We generated *RNF168*-KO hESC clone 44, which maintained pluripotency and genome integrity (Supplementary Fig 17A-D and Table 1). *RNF168*-KO hESCs were differentiated to NPCs, which expressed NPC markers similar to WT NPCs (Supplementary Fig 17E). RNA-seq analysis comparing 2 datasets each from *RNF168*-KO44 and WT NPCs revealed that upregulated genes were enriched in neuronal differentiation, translation and ribosome, and cell cycle transition (Supplementary Fig 17F) while downregulated genes were enriched in cilium movement, H3K27me3 targets, H3K4me3 targets, astrocyte markers, signaling pathways, and positive regulation of NPC proliferation (Supplementary Fig 17G). We performed 53BP1-pS25 CUT&RUN and showed that *RNF168*-KO disrupted 53BP1-pS25 localization on chromatin (Supplementary Fig 17H). *RNF168*-KO increased 53BP1-pS25 levels at genes enriched in neuronal differentiation, cell morphogenesis, and stem cell maintenance, whereas *RNF168*-KO decreased 53BP1-pS25 levels at genes enriched in cell cycle transition, signaling receptor regulation, anterior-posterior patterning, and transcription activator (Supplementary Fig 17I). The altered 53BP1-pS25 localization correlated with differential gene expression in RNF168-KO vs. WT NPCs (Supplementary Fig 17J). Altogether, these data suggest that DNA damage signaling regulates 53BP1 binding to chromatin, affecting genetic programs related to signaling pathways, protein translation, and NPC proliferation and differentiation."

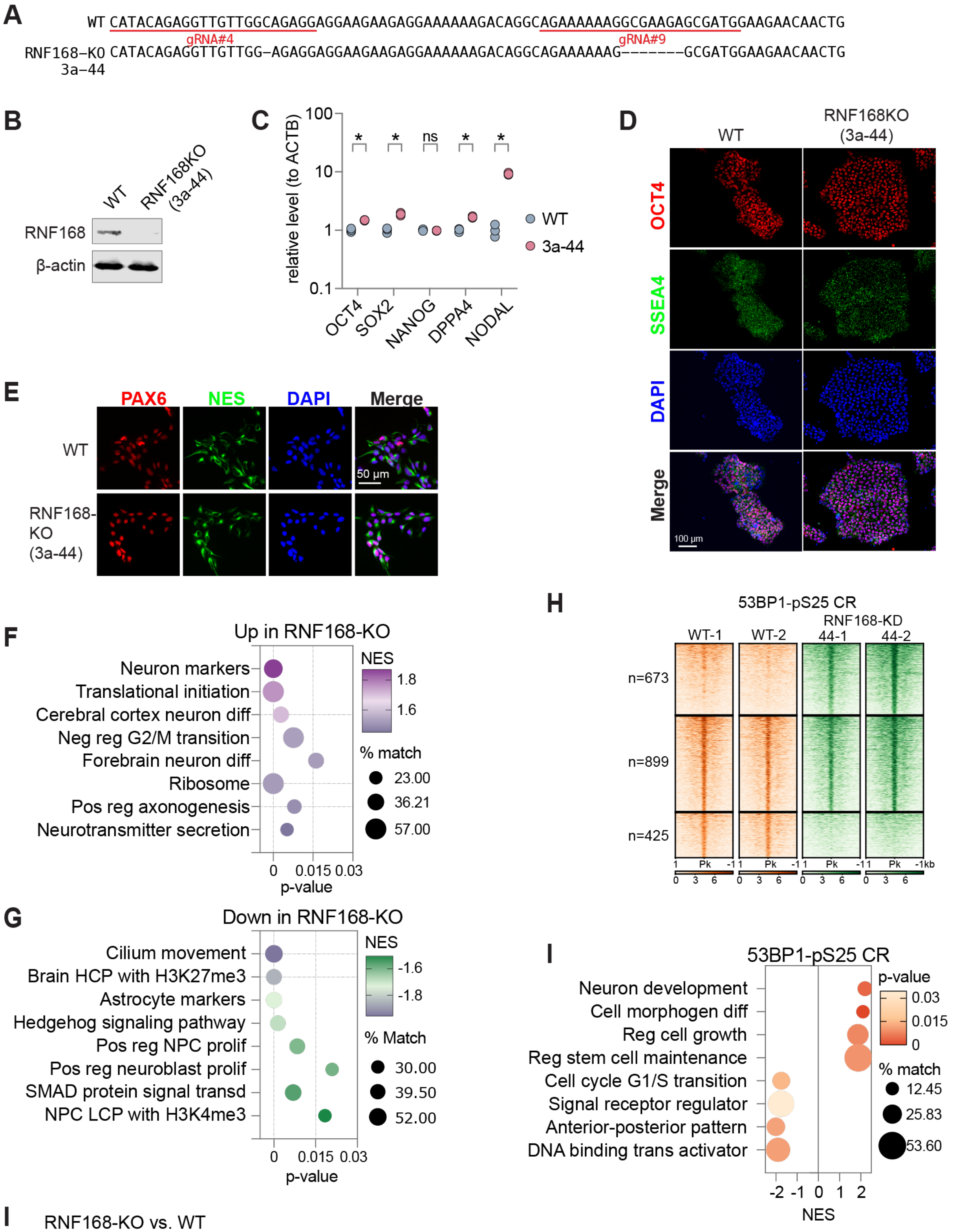

I

RNF168-KO vs. WT

	DEG	targets	higher	lower CR
Up	838	187	59	27
Down	923	171	17	57

p<0.05

Supplementary Figure 17. *RNF168*-KO alters key genetic programs and 53BP1-pS25 binding to chromatin.
(A) Alignment of WT and *RNF168*-KO mutation sequences in the *RNF168* locus. Red indicates the gRNA sequences.
(B) WB analysis of WT and *RNF168*-KO hESCs.
(C) RT-qPCR analysis showing that pluripotent genes in *RNF168*-KO were expressed higher or the same as those in WT. *RNF168*-KO did not reduce pluripotent gene expression. *, $p<0.05$; ns, not significant by xx {Yurika?} text.
(D) Immunofluorescence showed similar expression of OCT4 and SSEA4 proteins in WT and *RNF168*-KO hESCs. Bar, 100 µm.
(E) Immunofluorescence of showed similar expression of PAX6 and NES in NPCs. WT and *RNF168*-KO NPCs. Bar, 50 µm.
Functional terms that are highly enriched in (F) upregulated and (G) downregulated genes in *RNF168*-KO D35 cortical organoids. NES, normalized enrichment score. % Match, % of genes in the enriched term that overlap the differentially expressed genes or proteins.
(H) Heatmaps aligning peaks with 53BP1-pS25 CUT&RUN signals that were gained, the same, or lost in *RNF168*-KO vs. WT NPCs, using the criterion of fold-change (FC)>2 and $p<0.05$. n= numbers of peaks. Regions with the same signals, are n=899, which showed the least changes after voom normalization and served as semi-independent validation of differential ChIP-seq analysis.
(I) Functional terms that were enriched in genes with altered 53BP1-pS25 binding in *RNF168*-KO vs. WT NPCs. NES, normalized enrichment score. % Match, % of genes in the enriched term that overlap the differentially bound genes.
(J) Number of differentially expressed genes identified by comparison of *RNF168*-KO vs. WT NPCs at $p<0.05$. Of these genes, we list the numbers of 53BP1-pS25-bound targets and targets with higher or lower 53BP1-pS25 CUT&RUN signals in *RNF168*-KO NPCs.

- Line 93-94: "By D55, a subset of ATM-KO cortical organoids appeared smaller than controls, but this difference was not statistically significant (Supplementary Fig 3A-B). " - if it not significant this conclusion should be removed.

We revised the text to “By D55, *ATM*-KO cortical organoids had similar size distribution as the control (Supplementary Fig 5F-G).” in line 117.

- Line 33-36 "to localize to chromatin with double-stranded breaks, 53BP1 uses its BRCT domain to bind to gH2AX, the Tudor domain to bind to H4K27 dimethylation, and its UDR segment to bind to ubiquitinated H2AK15 (7, 8, 9, 10). " is incorrect; 53BP1 tudor recognise H4K20-methylation not this other state.

We appreciate the opportunity to correct this error. The text has been corrected.

Reviewer #2: In the manuscript “Phosphorylation of 53BP1 by ATM controls neurodevelopmental programs in cortical brain organoids" by Lim et al., the authors generated ATM-KO brain organoids and found severe neural developmental defects in these organoids. Next, they generated hESC lines, in which 53BP1-S25 cannot be phosphorylated by ATM anymore, by mutating serine 25 to alanine or aspartic acid using CRISPR/Cas9. By generating brain organoids from these cell lines, the authors found that 53BP1-S25A and 53BP1-S25D brain organoids - while different in comparison to the ATM-KO organoids - exhibit abnormal neural development. Lastly, the authors identified direct target genes of 53BP1 by ChIP-seq. Thus, the authors provide mechanistic insight into the role of 53BP1 phosphorylation by ATM during neural development. As this study is very interesting and provides novel insight into the role of ATM and 53BP1 during neural development, it seems to be suitable for publication in PLOS Biology. However, I have some concerns, which need to be addressed before considering the manuscript for publication. Mainly, the phenotypic analysis of the ATM-KO, 53BP1-S25A and 53BP1-S25D brain organoids in terms of analyzed time points (1 to maximum 2 points depending on the mutation) and features/markers studied is not sufficient to draw the conclusions that the authors have drawn. A more thorough phenotypic analysis is needed to understand which neurodevelopmental processes are disturbed in these brain organoids and which contribution ATM and 53BP1 have in this process. For details, please see below.

We appreciate these suggestions for improving our analyses and the entire manuscript.

1) Almost all analyses of brain organoids were performed at d35 with the exception of a few analyses at d55. On which basis has this time point of analyses been chosen? Brain organoids change during development in terms of morphology and cell type composition, so one or two time points of analyses are normally not sufficient. Could the authors please address the following questions? Was this the only time point when the phenotype was present or strongest? How do early-stage brain organoids look like? When did first abnormalities appear? For completeness, it would be good to add this information to the manuscript.

We appreciate the opportunity to improve our analytical of mutant phenotypes. We chose D35 to focus our analyses because it was the earliest time point that the 53BP1-S25A and S25D cortical organoids were consistently significantly smaller than WT.

We analyzed D14, D21, D28, and D35 cortical organoids. We performed fluorescence-activated cell sorting (FACS) analyses of proliferation marker KI67, differentiation marker CTIP2, NPC marker PAX6, and apoptotic marker cleaved-caspase 3. We also performed immunofluorescence of PAX6, ZO-1, and CTIP2 to study D21, D28, and D35 cortical organoids. Data from FACS enabled us to identify D28 as the time point that mutant cortical organoids consistently exhibited cellular defects. We did not include most of the negative results to streamline data presentation.

Altogether, the expanded analyses indeed helped us better understand the effects of the mutations on cortical organoids. Below, we specifically responded points 2-5 and provided text and figure additions to the manuscript at the end of our response to point 5.

2) In line with the previous question, in d35 ATM-KO organoids, at least in the pictures shown in Figure 1G, no ventricle-like structures are visible. This raises the question, if these structures degenerated during brain organoid development or if they were never present?

ZO-1 quantification suggest that *ATM*-KO ventricular surfaces did not differ from that in WT at D28 (Supplementary Fig 5B-D) but decreased by D37 (Fig 1G-I, 2D). It is not clear whether the ventricular surfaces indeed become degenerated during cortical organoid development. Cellular

disorganization around ventricular surfaces and higher neuronal differentiation (Fig 2) likely also contributed to the developmental defects.

3) The ATM-KO brain organoid exhibit many PAX6-positive cells but these cells are not (well) organized in VZ-like structures (not densely packed, no radial organization). This raises several questions. (i) How did the authors identify the VZ (size)? Would ZO-1 stainings helpful to identify the ventricular surface? (ii) Are the Pax6-positive cells apical radial glia (with an apical contact, here ZO-1 stainings could be helpful again) or did they differentiate to a different progenitor type (without apical contact; basal progenitors?)? (iii) It seems so that there are many more Pax6-positive cells in ATM-KO in comparison to WT brain organoids. Is this true? Could the authors please quantify Pax6-positive cells in WT and ATM-KO brain organoids? This seems to also be the case for Ki67. Could the authors also quantify Ki67? These data would be important to better understand the effects of ATM on neural development.

(i) We identified the VZ-like regions as PAX6-positive areas that are bordered by CTIP2-positive cells. We added this information to the Methods section. ZO-1 signals help identify the apical ventricular surfaces.

(ii) The analysis of ZO-1 immunofluorescence images in Fig 2D and Supplementary Fig 5B-E suggests that WT and *ATM*-KO indeed generate apical radial glia.

(iii) Using FACS and immunofluorescence, we quantified PAX6, KI67, and PH3 in D21, D28, and D35 cortical organoids. To streamline data presentation, we presented D28 and D35 data: KI67/DAPI, PH3/DAPI, and KI67+PAX6+/PAX6 in ATM-KO did not significantly differ from those in WT (Supplementary Fig 4).

4) In line 92/93 authors claim to observe reduced neuronal differentiation. How do the authors know this? From the images presented in Figure 1G, it seems so that this is true for some ATM-KO clones but not for all. Could the authors validate this by quantifications. Moreover, the authors present just CTIP2 stainings, which is a marker for deep-layer neurons. Could it be that just deep-layer neurons are affected? Why not using a general neuronal marker like NeuN? Would upper-layer neurons at later stages also be affected?

We are thankful for the opportunity to improve our phenotypic observations.

Also in response to Reviewer 2's comment, we quantified NPC marker PAX6, immature neuronal marker NEUN, and mature neuronal marker CTIP2 in D28 and D37 cortical organoids. At both D28 and D37, PAX6/CTIP2 in *ATM*-KO were significantly lower than those in WT (Fig 2A-B), but NEUN/DAPI in *ATM*-KO was lower than that in WT (Fig 2C). These data suggest that *ATM*-KO shortens the immature neuronal phase to fasten to the neuronal maturation phase.

5) How do the authors know that there are lower levels of proliferation in ATM-KO organoids? For this they would need to quantify the number of KI67 positive cells (see above). Information on mitotic (e.g. PH3 positive) cells would also be helpful, as apical radial glia normally divide at the ventricular surface. Is there reduced mitotic cells because there is no ventricular surface or can these cells now divide freely within in the organoid? ZO-1 stainings in combination with PH3 would be helpful.

We quantified KI67/DAPI, PH3/DAPI, and the organization of KI67-positive and PH3-positive cells in combination of ZO-1-positive ventricular surfaces.

Combining results contributing to our response to points 2-5, we added to lines 99-120, "We quantitatively compared NPC proliferation, neuronal differentiation, cell death, and cell organization in *ATM*-KO versus WT cortical organoids. Examination of endogenous DNA damage, by γH2AX immunofluorescence, did not reveal marked difference (Supplementary Fig 3A), confirming our WB results in Supplementary Fig 1I. Although quantification of cell death marker cleaved-caspase 3 by FACS revealed a modest increase of cell death in D21 ATM-KO cortical organoids, FACS and immunofluorescence quantification showed that D28 and D35 *ATM*-KO and WT cortical organoids are similar in cell death frequencies (Supplementary Fig 3B-E). Cell proliferation frequencies did not significantly differ between D28 and D35 *ATM*-KO and WT cortical organoids (Supplementary Fig 4). We next quantified immature neuronal marker NEUN and PAX6/CTIP2 ratios. Despite lower levels of immature neuronal differentiation, ATM-KO exhibited higher neuronal maturation (Fig 2A-C, Supplementary Fig 5A). These data suggest that *ATM*-KO fastens the phase of immature neuronal differentiation, leading to enhanced neuronal maturation. Finally, we quantified ZO-1-positive ventricular surfaces and the organization of PH3-positive and PAX6-positive cells around ventricular surfaces. The *ATM*-KO ventricular surfaces were similar to WT at D28 (Supplementary Fig 5B-D) but the number was much reduced by D37 (Fig 2D). NPC organization around the ventricular surfaces that formed were similarly organized in D37 (Fig 2D, Supplementary 5E); however, fewer *ATM*-KO proliferative cells were adjacent to ventricular surfaces (Fig 2E). These data suggest that ATM-KO enhances neuronal maturation and cellular disorganization in developing cortical organoids."

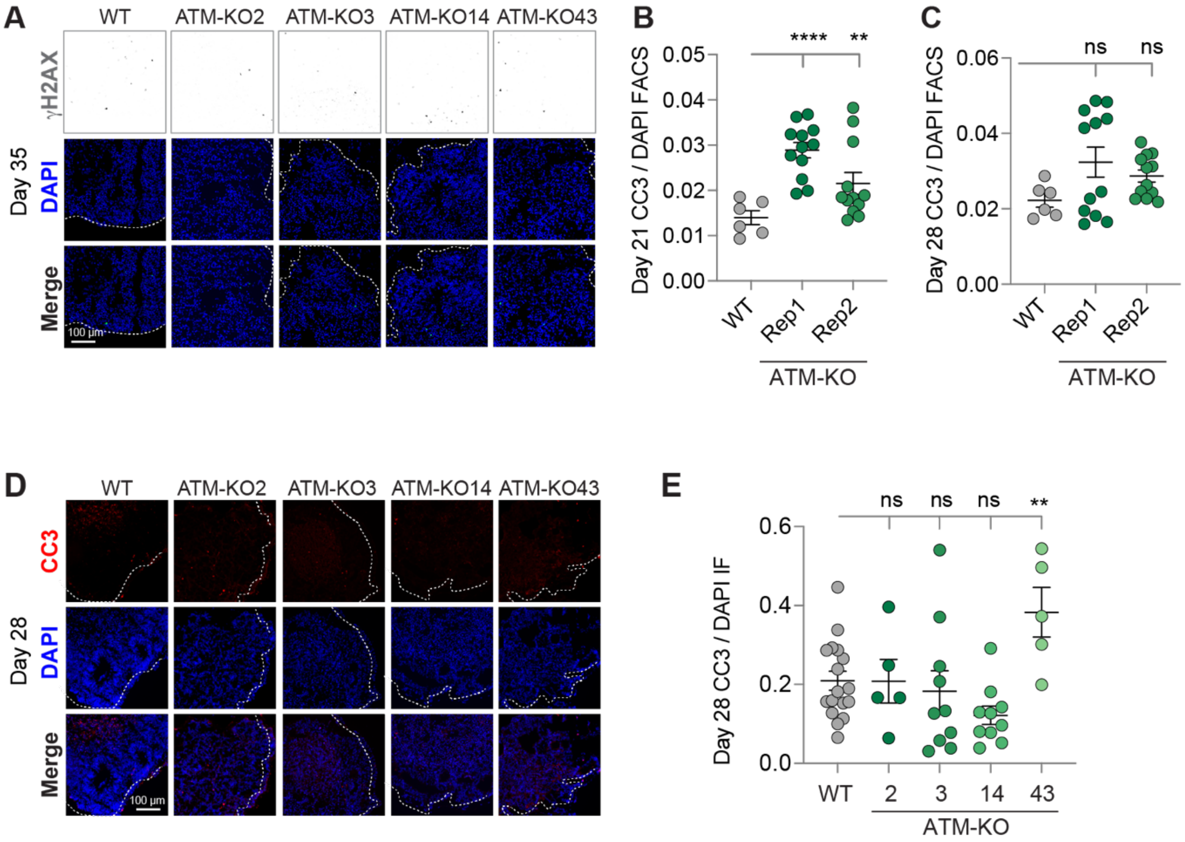

Supplementary Figure 3. Analysis of gH2AX and cleaved-caspase 3 (CC3) in cortical organoids.
(A) Immunofluorescence showed D35 *ATM*-KO and WT cortical organoids had similar γH2AX foci. Bar, 100 μm.
FACS analysis of CC3 in (B) D21 and (C) D28 cortical organoids. Two biological replicates were done, and each data point was based on 3 technical replicate analyses of 10-12cortical organoids.
(D, E) Immunofluorescence and quantification of CC3 in D28 cortical organoids. Bar, 100 μm.
Graphs are presented in ratios (out of 1), with **, $p<0.01$; ****, $p<0.0001$; ns, not significant by Two-way ANOVA test.

For figure below:
Supplementary Figure 4. Analysis of cell proliferation in cortical organoids.
FACS analysis of PAX6 and KI67 in (A, B) D28 and (C) D35 cortical organoids. Each data point was based on the 3 technical replicate analyses of 10 cortical organoids.
(D) Quantification of KI67/PAX6 ratios in immunofluorescence of D35 cortical organoids. Each data point represents quantification of cells in 1 cortical organoid.
(E, F) Immunofluorescence and quantification of H3-pS10 (PH3) in D28 cortical organoids. Bar, 100 μm.
(F-H) Immunofluorescence and quantification of PH3 and KI67 in D35 cortical organoids. Bar, 100 μm.
***, $p<0.001$; ns, not significant by Two-way ANOVA test.

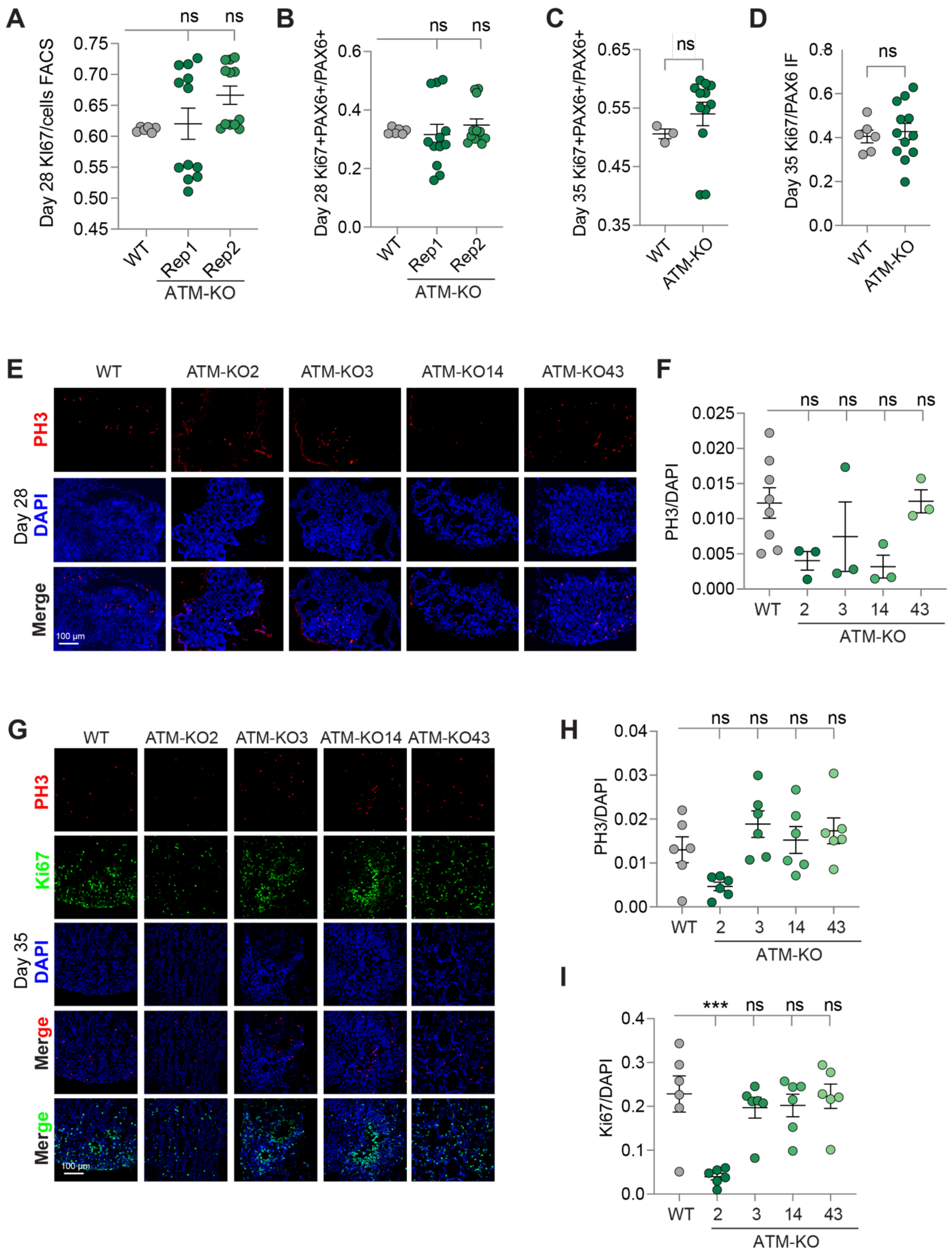
A
Day 28 KI67/cells FACS
0.75
0.70
0.65
0.60
0.55
0.50
0.45
ns
ns
WT
Rep1
Rep2
ATM-KO
B
Day 28 Ki67+PAX6+/PAX6+
0.6
0.4
0.2
0.0
ns
ns
WT
Rep1
Rep2
ATM-KO
C
Day 35 Ki67+PAX6+/PAX6+
0.65
0.55
0.45
0.35
ns
WT
ATM-KO
D
Day 35 Ki67/PAX6 IF
0.8
0.6
0.4
0.2
0.0
ns
WT
ATM-KO
E
WT
ATM-KO2
ATM-KO3
ATM-KO14
ATM-KO43
PH3
Day 28 DAPI
Merge
100 µm
F
PH3/DAPI
0.025
0.020
0.015
0.010
0.005
0.000
ns
ns
ns
ns
WT
2
3
14
43
ATM-KO
G
WT
ATM-KO2
ATM-KO3
ATM-KO14
ATM-KO43
PH3
Ki67
Day 35 DAPI
Merge
Merge
100 µm
H
PH3/DAPI
0.04
0.03
0.02
0.01
0.00
ns
ns
ns
ns
WT
2
3
14
43
ATM-KO
I
Ki67/DAPI
0.4
0.3
0.2
0.1
0.0
***
ns
ns
ns
WT
2
3
14
43
ATM-KO

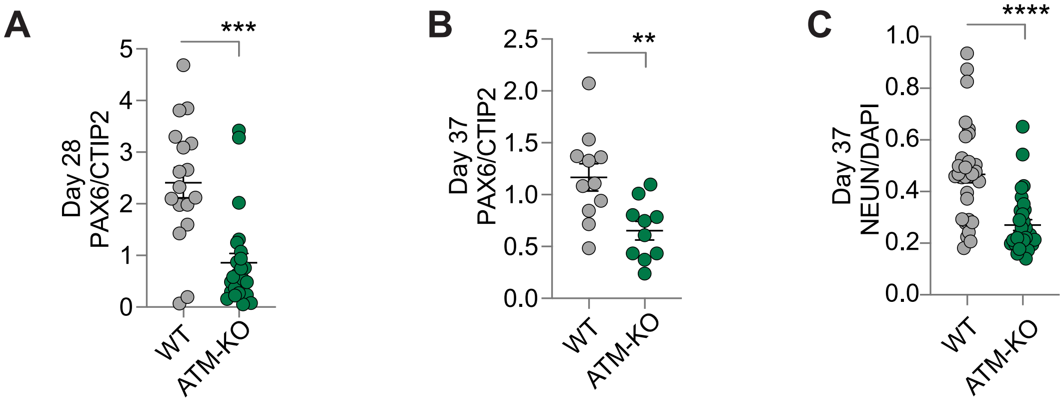

D

Day 37

**Line**	**#ZO1 rings**	**% organized PAX6**
WT	27	77.78
ATM-KO	7	85.71

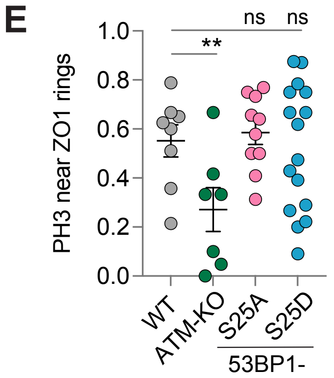

Figure 2. Analysis of neuronal differentiation and cell organization in cortical organoids.
Quantification of PAX6/CTIP2 ratios in (A) D28 and (B) D37 cortical organoids.
(C) Quantification of NEUN/DAPI in D37 cortical organoids.
(D) In D37 cortical organoids, 6 organoids were surveyed to count ZO-1-positive apical surfaces and proportions of PAX6-positive NPCs that are organized around the apical surfaces.
(E) Proportions of PH3-positive cells that are adjacent to ZO-1-positive apical surfaces (“rings”). Data from 53BP1-S25A and S25D were included for comparison.
**, p<0.01; ***, p<0.001; ****, p<0.0001; ns, not significant by Welch’s t-test in (A-C) and Two-way ANOVA test in (D).

For figure below:
Supplementary Figure 5. Immunofluorescence analyses of cortical organoids and NPCs.
Immunofluorescence of (A) NEUN and (E) ZO-1 and PAX6 in D37 cortical organoids. Bar, 100 µm.
Quantification of the (B) number and (C) surface area of ZO-1-positive ventricles.
(D) Immunofluorescence of ZO-1 in D28 cortical organoids. Bar, 100 µm.
(F-H) Immunofluorescence of PAX6 and NES in NPCs. Bar, 50 µm.
*, p<0.05; ***, p<0.001; ns, not significant by Two-way ANOVA test.

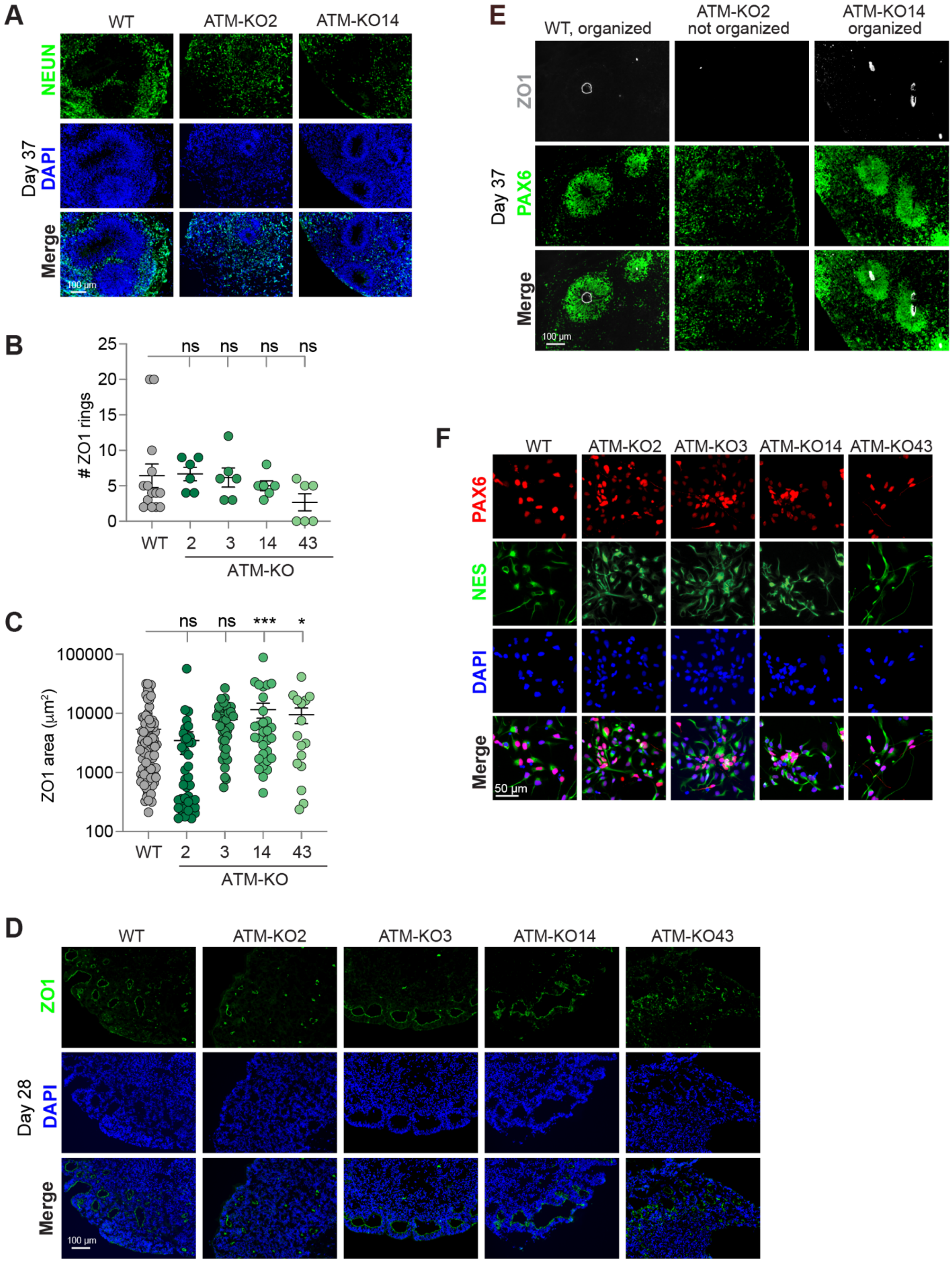
A
WT
ATM-KO2
ATM-KO14
NEUN
Day 37 DAPI
Merge
100 µm
B
ns ns ns ns
ZO1 rings
25
20
15
10
5
0
WT 2 3 14 43
ATM-KO
C
ns ns *** *
ZO1 area (µm²)
100000
10000
1000
100
WT 2 3 14 43
ATM-KO
D
WT
ATM-KO2
ATM-KO3
ATM-KO14
ATM-KO43
ZO1
Day 28 DAPI
Merge
100 µm
E
WT, organized
ATM-KO2 not organized
ATM-KO14 organized
ZO1
Day 37 PAX6
Merge
100 µm
F
WT
ATM-KO2
ATM-KO3
ATM-KO14
ATM-KO43
PAX6
NES
DAPI
Merge
50 µm

6) WT and ATM-KO brain organoids seem to have a very different cell type composition. This might bias the transcriptome analyses: Instead of getting mechanistic insight in what ATM-KO is changing within a given cell population, one would get differences which are caused by different cell type composition. Here, single cell RNAseq or enrichment for certain cell populations (e.g. progenitors vs neurons) would be helpful. Another option would be to perform transcriptomics on NPCs, which the authors could easily produce from WT and ATM-KO cells.

We appreciate this suggestion and performed RNA-seq analysis *ATM*-KO and WT NPCs. These analyses showed that ATM-KO NPCs and cortical organoids had similar altered genetic programs, suggesting that ATM indeed regulates key genetic programs in NPCs and cortical organoids.

We added to line 123-136 (replacing some texts), “To investigate the molecular basis of the cellular defects we observed in *ATM*-KO, we performed RNA-seq analyses to compare *ATM*-KO to WT NPCs and D35 cortical organoids derived from WT and *ATM*-KO hESCs. The expression of forebrain markers was similar between WT and *ATM*-KO (and low expression of midbrain and hindbrain markers; Supplementary Table 2), suggesting that the *ATM*-KO cortical organoids specified to the forebrain lineage. Using a false discovery rate (FDR) <0.05, we identified 590 upregulated genes and 1,084 downregulated genes in 8 *ATM*-KO versus 6 WT datasets. A false discovery rate (FDR) <0.05 was used to identify differentially expressed genes. Gene set enrichment analysis (GSEA) showed that upregulated genes in *ATM*-KO NPCs were enriched in forebrain development, axis specification, and metabolic pathways (Fig 2F and Supplementary Fig 6A), whereas downregulated genes were enriched in neuronal differentiation, epithelial mesenchymal transition, and tube morphogenesis (Fig 2G). Comparison of transcriptomes profiles of D35 cortical organoids from 8 *ATM*-KO versus 6 WT datasets yielded similar GSEA terms (Fig 3A-B). These data suggest that ATM regulates genetic programs related to forebrain development, metabolism, neuronal differentiation in NPCs and cortical organoids. Dysregulated genetic programs likely contributed to enhanced neuronal maturation and cellular disorganization in *ATM*-KO cortical organoids.”

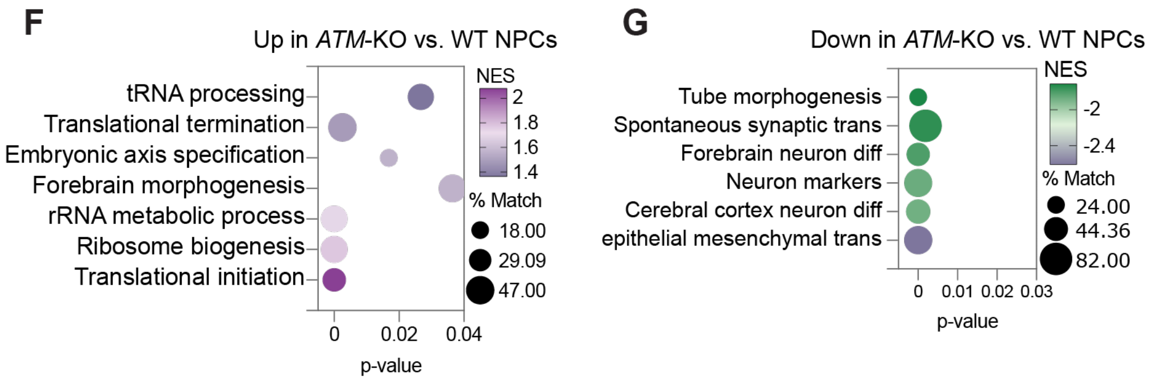

Figure 2. From gene set enrichment analysis, functional terms that are highly enriched in (F) upregulated and (G) downregulated genes in *ATM*-KO vs. WT NPCs. NES, normalized enrichment score. % Match, % of genes in the enriched term that overlap the differentially expressed genes or proteins.

7) Brain organoids and KO lines were derived from one cell line (H9/WA09). Would these phenotypes also be observed, if another cell line would be used in this study? Organoid studies normally using more than one cell line to address biological variation.

We appreciate the opportunity to address this question. Our study focuses on the fundamental and noncanonical role of DNA damage response in developmental gene regulation. To analyze ATM-

KO, 53BP1-S25A, and 53BP1-S25D, we enhanced research rigor by using 4 separate mutant lines for analyses. The scope of our overall study does not permit the use of a separate parental cell line for generating and quality-controlling mutation lines.

8) As ATM phosphorylates 53BP1 and therefore at least partially functions through 53BP1 phosphorylation, I would expect 53BP1-S25A and 53BP1-S25D to have similar phenotypes as ATM-KO. However, the phenotypes are quite different, e.g. number and organization of PAX6-positive cells. How do the authors interpret this? Could the authors please add their considerations to the discussion part?

This is an important point. Additional analyses showed that accelerated neuronal maturation and cellular disorganization in *ATM*-KO, 53BP1-S25A, and 53BP1-S25D are more similar than we originally observe. We hope the Reviewer agrees that, given the new data, we have included these findings in Results and have chosen not to make drastic changes to the Discussion.

We performed additional, separate analyses of the RNA-seq datasets from D35 cortical organoids of WT, ATM-KO, 53BP1-S25A, and 53BP1-S25D (Fig 4G, Supplementary 7E-G). We added in “Results,” pg 299-301, “GSEA showed a significant enrichment of concordantly differentially expressed genes amongst ATM-KO vs. WT, 53BP1-S25A, and 53BP1-S25D (Fig 6G, Supplementary Fig 13A-C).”

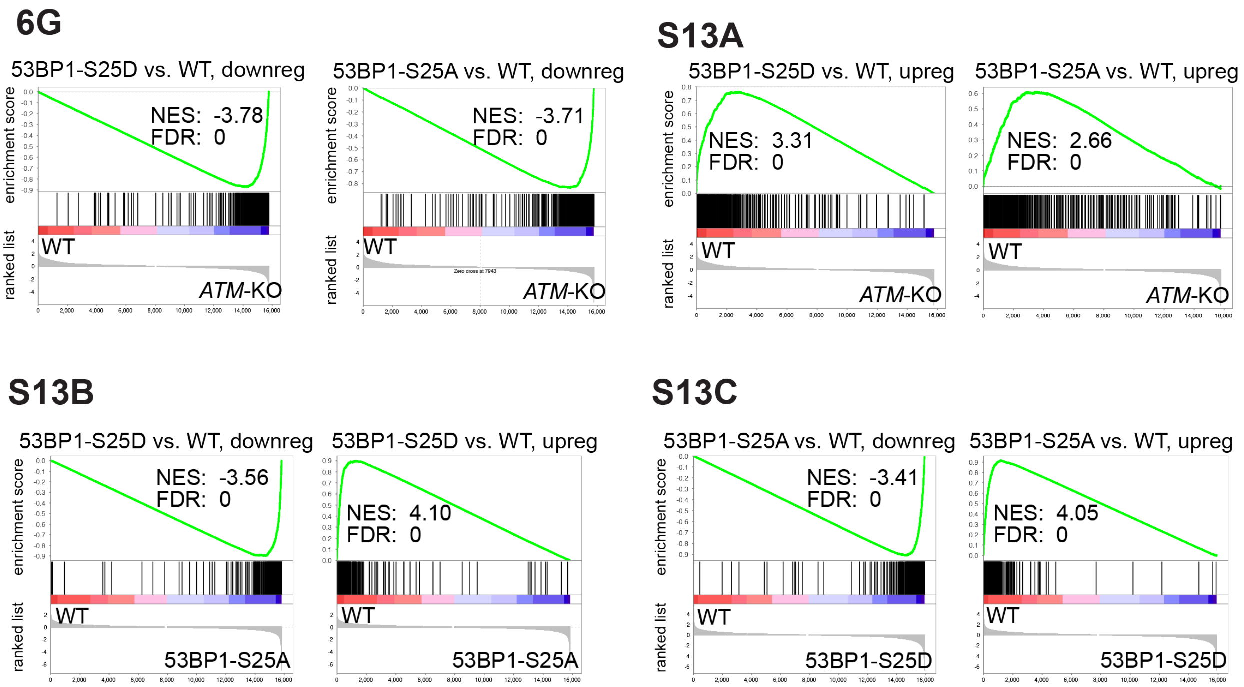

GSEA showed that differentially expressed genes in ATM-KO vs. WT were significantly and concordantly enriched in those found in (Fig 6G) 53BP1-S25D vs. WT and (Supp Fig 13A) 53BP1-S25A vs. WT. (Supp Fig 13B, C) Similar GSEA results were obtained when analyzing differentially expressed genes in 53BP1-S25A vs. WT and 53BP1-S25D vs. WT. *P* values were calculated by the hypergeometric test, assuming normal data distribution.

9) The number of CTIP2-positive cells, from the pictures presented in Figure 3D, looks very different between the various 53BP1-S25A and 53BP1-S25D clones. Could the authors please quantify CTIP2-positive cell numbers and compare between 53BP1-S25A and 53BP1-S25D clones and WT?

In Fig 3D, we had replaced images for 53BP1-S25D 14-15, 14-19, and 17 to better reflect the similarity between 53BP1-S25A and S25D clones. We thank the Reviewer for improving our data presentation. In our original submission, we had quantified CTIP2/DAPI (original Supplementary Fig 6A), which we replaced with the PAX6/CTIP2 ratios to reduce confusion for the readers. In response to Reviewer 3's point 3, we had calculated the PAX6/CTIP2 ratios, which improved our response to point 10 below.

10) Why are there fewer progenitor cells in 53BP1-S25A and 53BP1-S25D organoids? Do they proliferate less or have a longer cell cycle or is there increased apoptosis or increased neuronal differentiation? As already mentioned above the characterization of brain organoids is very limited and needs to be expanded to understand the effect of 53BP1-S25A and 53BP1-S25D on neural development, especially as the transcriptomic studies seem not provide a clear mechanism.

We appreciate this opportunity to better understand the effects of 53BP1-S25A and S25D. Also in response to Reviewer 2's point 1, we analyzed cortical organoids at D14, D21, D28, and D35 of differentiation. We performed FACS analyses of proliferation marker KI67, differentiation marker CTIP2, NPC marker PAX6, and apoptotic marker cleaved-caspase 3. We also performed immunofluorescence of PAX6, ZO-1, and CTIP2 to study D21, D28, and D35 cortical organoids. Data from FACS enabled us to identify D28 as the time point that 53BP1-S25A and S25D cortical organoids consistently exhibited cellular defects. We added to line 230-235, "Examination of endogenous DNA damage and cell death, assessed by γH2AX and cleaved-caspase 3, respectively, did not reveal significant differences between 53BP1-S25A, S25D and WT (Supplementary Fig 9). To explore the developmental timing of the cellular phenotypes, we quantified KI67, NPC marker PAX6, and neuronal marker CTIP2 in D14, D21, D28, and D35 cortical organoids. At D28 and D35, 53BP1-S25A and S25D cortical organoids had significantly lower cell proliferation and higher neuronal differentiation (Fig 5)."

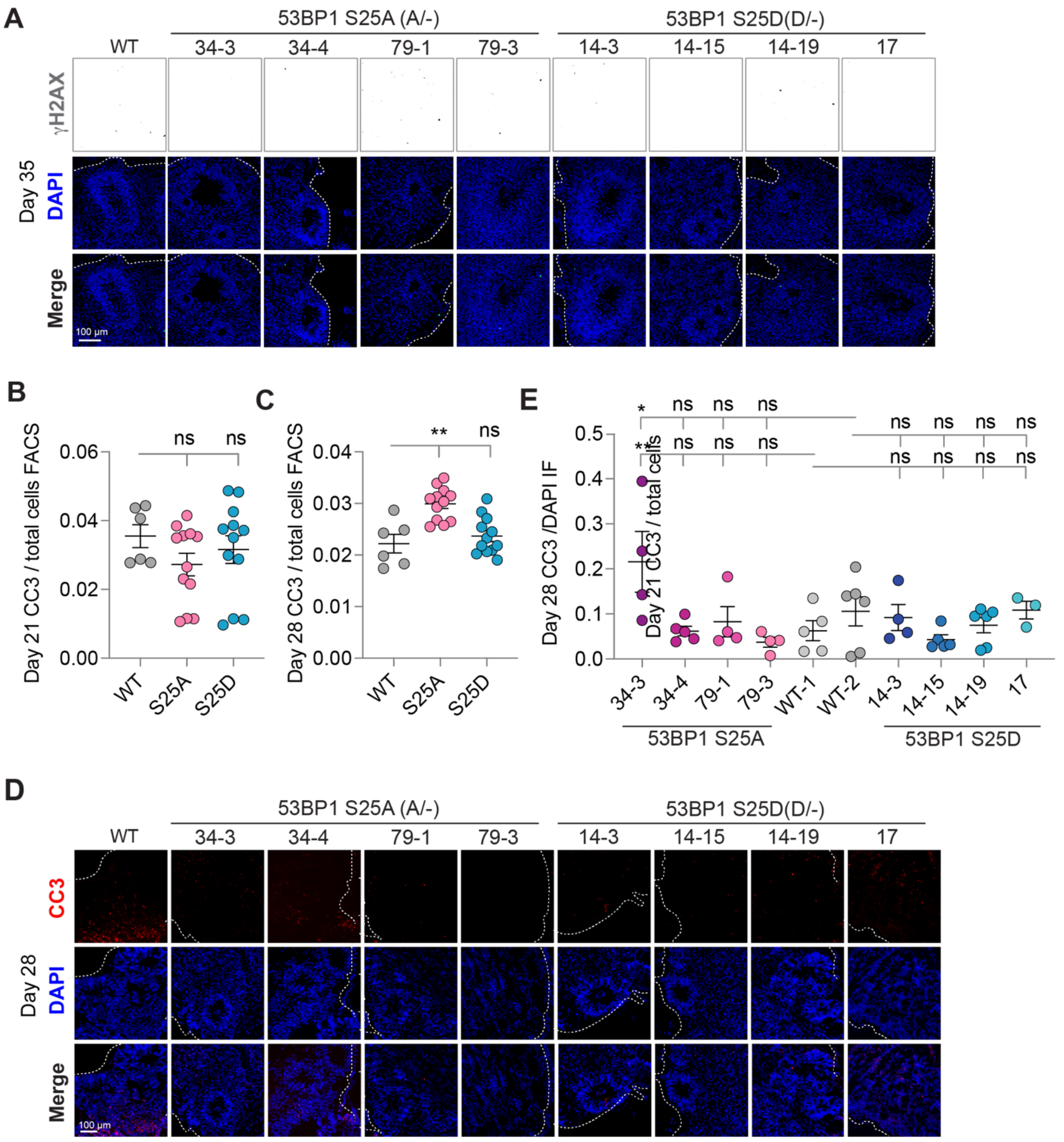

Supplementary Figure 9. Analysis of γH2AX and cleaved-caspase 3 (CC3) in cortical organoids. Immunofluorescence of (A) γH2AX in D35 cortical organoids and (D) cleaved caspase 3 (CC3) in D28 cortical organoids. Bar, 100 µm.

CC3 quantification by FACS of (B) D21 and (C) D28 cortical organoids. For each datapoint, 10-12 organoids from each line were analyzed via 3 technical replicates, and data from 4 mutant lines were consolidated to achieve rigorous comparisons. **, $p<0.01$ and ns, not significant by Two-way ANOVA test.

(E) CC3 quantification of immunofluorescence images of D28 cortical organoids. For each line, 4-6 images and >10,000 cells were analyzed. *, $p<0.05$; **, $p<0.01$; ns, not significant by Two-way ANOVA test.

Graphs in (B, C, E) are presented in ratios (out of 1).

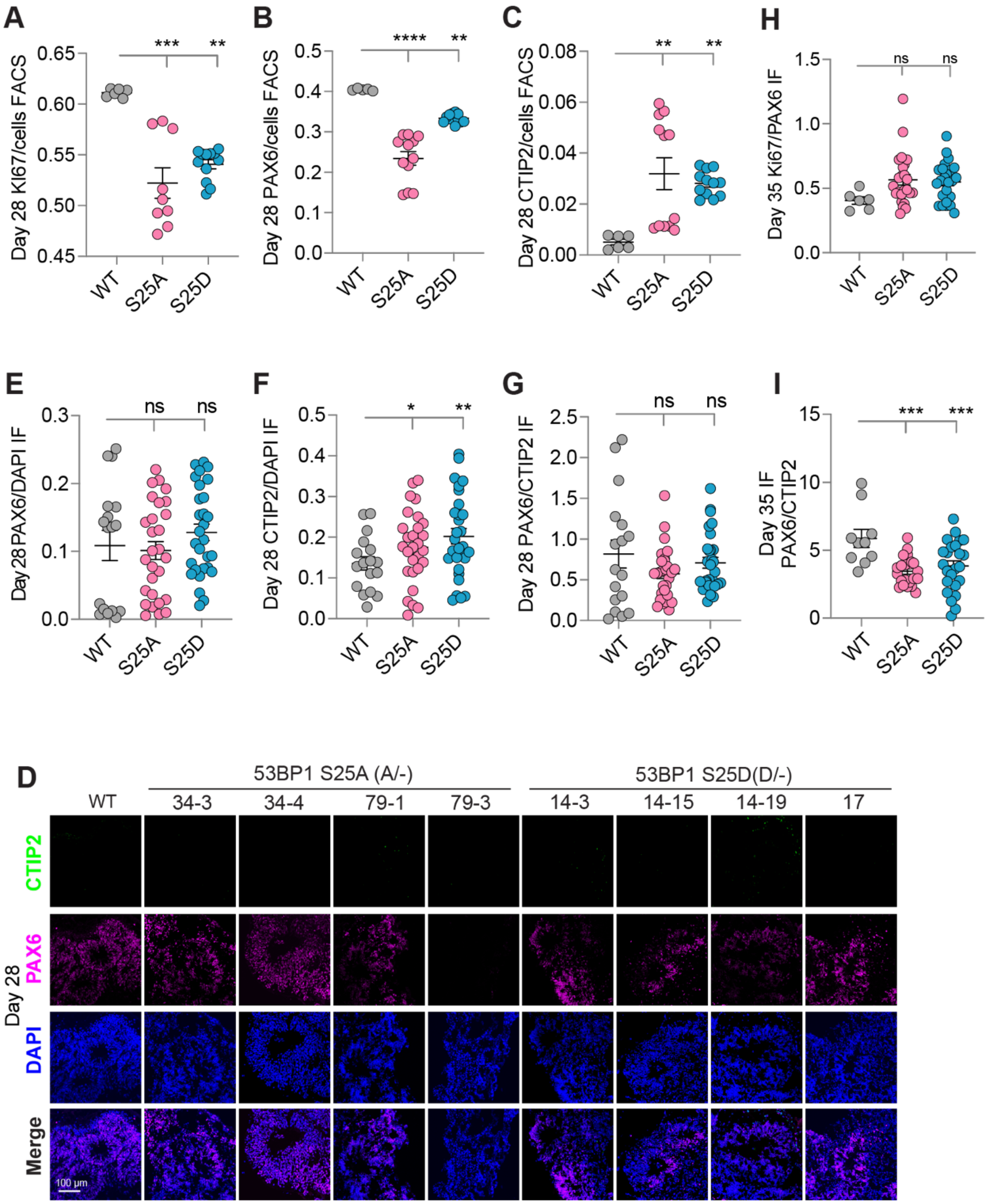

Figure 5. 53BP1-S25A and S25D lower cell proliferation in cortical organoids.
FACS quantified ratios of (A) KI67, (B) PAX6, and (C) CTIP2 to total cells in D28 cortical organoids. (D) Immunofluorescence of PAX6 and CTIP2 in D28 cortical organoids. Bar, 100 µm. Quantification of immunofluorescence signals of (E) PAX6/DAPI, (F) CTIP2/DAPI, and (G) PAX6/CTIP2 in D28 cortical organoids. Each data point represents quantification of cells in 1 cortical organoid.

Quantification of (H) KI67/PAX6 and (I) PAX6/CTIP2 ratios in immunofluorescence of D35 cortical organoids. Each data point represents quantification of cells in 1 cortical organoid.
*, $p<0.05$; **, $p<0.01$; ***, $p<0.001$; ****, $p<0.0001$; ns, not significant by Two-way ANOVA test.

11) The transcriptome analysis of 53BP1-S25A and 53BP1-S25D brain organoids does not provide a clear explanation of the observed phenotypes. As described in comment 6) might this be due to the different cell type composition between 53BP1-S25A, 53BP1-S25D and WT organoids? Could the transcriptome analysis not also be performed on cultured NPCs, as this would be a clean population and as this would provide a direct comparison to the ChIP-seq data which was performed on these cells anyway?

This suggestion helped us clarify our understanding of how 53BP1-S25A and S25D alter NPCs and neuronal differentiation.

We added to line 271-282, “It remained unclear whether the higher expression of neuronal genetic programs in the 53BP1 mutants occurred in NPCs or neurons. Therefore, we compared the transcriptomes of 53BP1-S25A and S25D to WT NPCs, which had similar expression of NPC markers PAX6 and NES (Supplementary Fig 12A). Upregulated genetic programs in 53BP1-S25A and S25D NPCs shared categories such as translation control and ribosome (Supplementary Fig 12B-C), whereas 53BP1-S25A NPCs also upregulated cell cycle control and chromosome segregation (Supplementary Fig 12C). Surprisingly, genes related to neuronal differentiation were downregulated in 53BP1-S25A and S25D NPCs (Supplementary Fig 12D-E). The downregulated genetic programs in NPCs are similar to neuronal programs that became upregulated in 53BP1-S25A and S25D versus WT cortical organoids. These data suggest that 53BP1-S25 phosphorylation promotes the appropriate expression of neurogenic programs in NPCs and fastens neuronal differentiation (initiated by differentiation cues) in cortical organoids.”

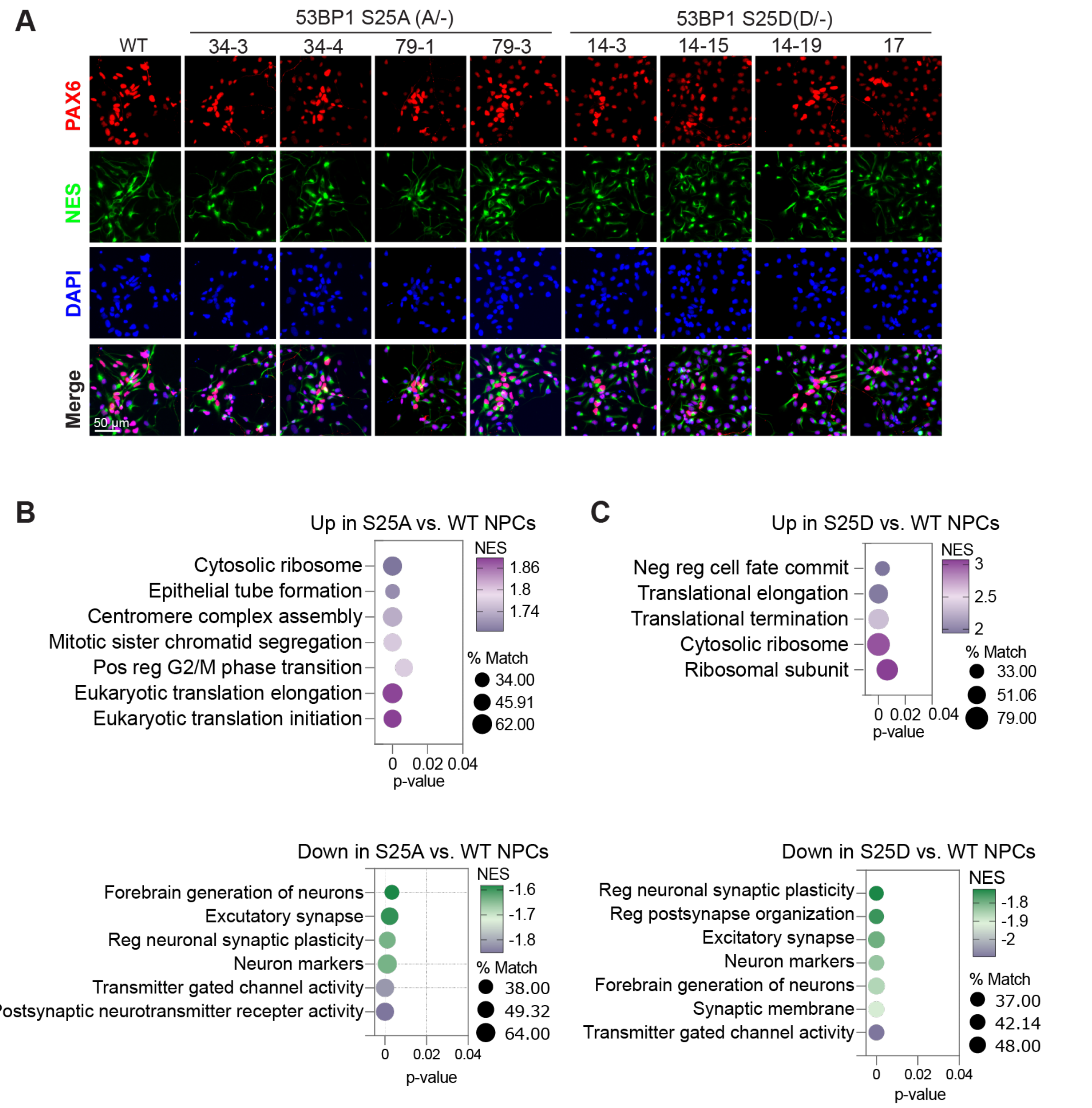

Supplementary Figure 12. Characterization of NPCs and comparative analyses of RNA-seq data. (A) Immunofluorescence of NPC markers PAX6 and NESTIN. Bar, 50 μm.
Gene set enrichment analysis identified top enrichment of differentially expressed genes in (B) 53BP1-S25A or (C) S25D versus WT NPCs. NES, normalized enrichment score. % Match, % of genes in the enriched term that overlap the differentially expressed genes or proteins.

Minor comments:

We appreciate the opportunity to correct these errors.

1) The legend of Figure 1A is missing.

The revised figure legend now has “(A)” label inserted to indicate the figure legends.

2) How did the authors confirm that the generation of NPCs were successful?

We generated the NPCs by using a standard commercial kit, STEMdiff SMADi Neural Induction Kit (STEMCELL Technologies, 05835). NPCs were then analyzed by immunofluorescence and RNA-seq.

We used RNA-seq analysis to compare WT control ESCs, NPCs, D10 cortical organoids, and D17 cortical organoids. Principle component analysis showed that the transcriptomes of WT NPCs were most similar to those of D17 cortical organoids (Supplementary Fig 1B); this is not surprising given NPCs were harvested at after 18 days of neural induction. GSEA showed that compared to WT ESCs, WT NPCs had downregulated ESC gene module (238/330 genes; Supplementary Fig 1C) and upregulated genetic programs related to development of forebrain, cerebral cortex, and neuron differentiation (Supplementary Fig 1C).

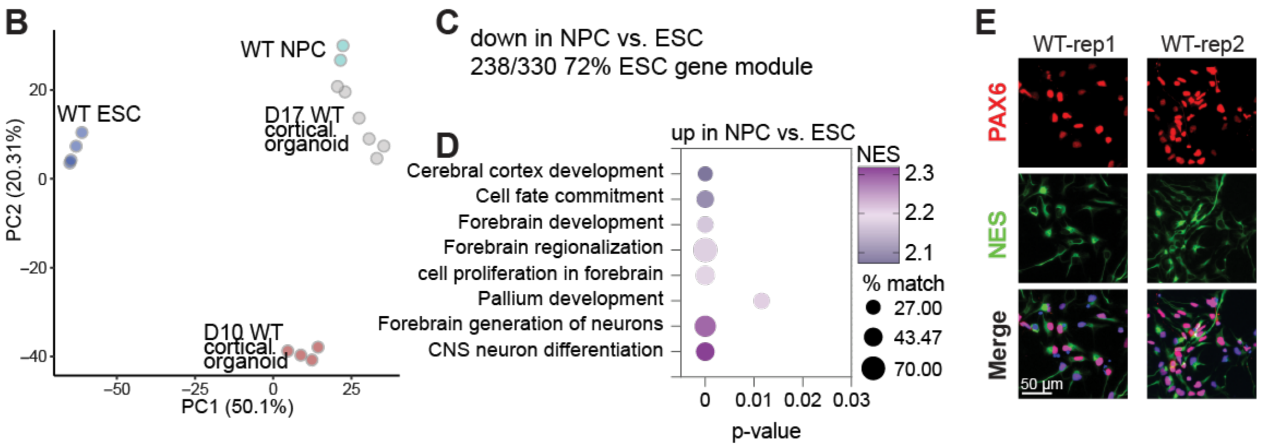

Supplementary Figure 1.

(B) Principal component analysis of WT ESCs, NPCs, day 10 (D10) cortical organoids, and D17 cortical organoids.

GSEA terms that are highly enriched in significantly (C) downregulated and (D) upregulated genes in WT NPCs compared to ESCs. NES, normalized enrichment score. % Match, % of genes in the enriched term that overlap the differentially expressed genes or proteins.

(E) Immunofluorescence of NPC markers PAX6 and NESTIN. Bar, 50 μm.

3) While the authors have checked, if their CRISPR/Cas9-edited cells were still pluripotent, they have not checked, if the karyotype of these cells is still normal.

We have analyzed the cell lines, including the 2 newly made RNF168-KO lines. These cell lines have normal karyotype. Representative data are presented in Supplementary Table 1 with accompanied text changes in lines 87-89, “All cell lines underwent karyotyping analysis and were characterized as karyotypically normal (Supplementary Table 1). *ATM*-KO lines had minor abnormalities, as expected due to the requirement of ATM for DNA damage repair.”

Supplementary Table 1

Cell line	# cells analyzed	Karyotype normal	Karyotype abnormal
**ATM-KO 2**	30	97% 46,XX	3% 46,XX,del(11-q13)
**ATM-KO 3**	40	75% 46,XX	25% 47,XX,+8
**ATM-KO 14**	20	100% 46,XX	0%
**ATM-KO 43**	30	97% 46,XX	3% 46,XX,del(11-q21)
**53BP1-S25A 34-3**	20	100% 46, XX	0%
**53BP1-S25A 34-4**	20	100% 46, XX	0%
**53BP1-S25A 79-1**	20	100% 46, XX	0%
**53BP1-S25A 79-3**	20	100% 46, XX	0%
**53BP1-S25D 14-3**	20	100% 46, XX	0%
**53BP1-S25D 14-15**	20	100% 46, XX	0%
**53BP1-S25D 14-19**	20	100% 46, XX	0%
**53BP1-S25D 17**	20	100% 46, XX	0%
**RNF168-KO 3a-44**	20	100% 46, XX	0%

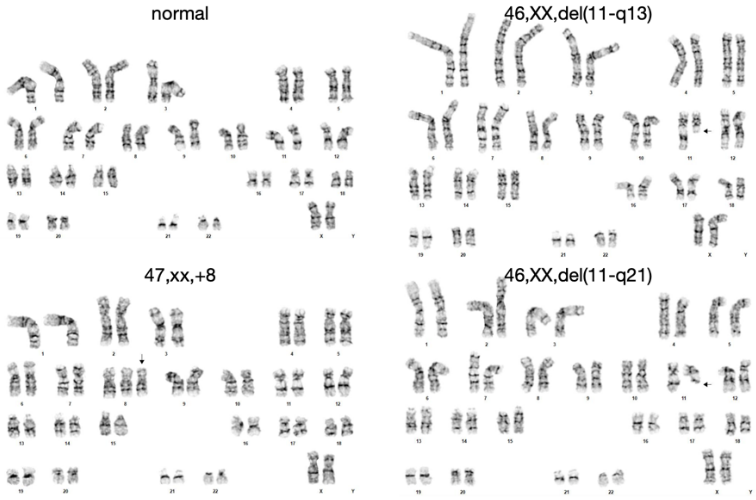

Supplementary Table 1. All cell lines generated for this study were treated with trypsin and Wright's stain, then analyzed by the Cytogenetic Shared Resource at St. Jude. Typically normal karyotypes and 3 abnormalities are shown.

4) In Figure 1F and Supplemental Figure S2B, why is there a band visible for ATM in case of some ATM-KO cell lines?

There are multiple possible reasons for cross-contamination of wildtype and ATM-KO samples during the initial WB analyses; the same pipet tips were used for changing the culture media, preparing the protein samples, or loading the protein samples into the gel. We now have repeatedly performed ATM WB to analyze the ATM-KO lines and showed that proteins were completely depleted. We apologize for this error and have corrected the figure panels.

5) In Figure 2E, what do the error bars indicate?

The revised figure legend now contains "The error bars depict the mean and standard error of the mean values, which were calculated based on the normalized levels of each phospho-peptide in the protein."

Reviewer #3: In this study, Lim, Djekidel and colleagues investigate the role of ATM and its target 53BP1 in cortical development. After confirming that 53BP1 is a target of ATM on residue S25, they generate a series of KO and KI ES cell lines for these factors and generate cerebral organoids. In both ATM KO and 53BP1 SA and SD, they observe major developmental defects, including proliferation, fate and overall growth. Because ATM expression and 53BP1 phosphorylation increase between ES cells and NPCs in the absence of increased gamma-H2AX, the authors propose that the observed function of 53BP1 phosphorylation is independent of DNA damage. They next use an extensive array of omics methods to better characterise these defects, including transcriptomics, proteomics, phospho-proteomics and ChIP-seq. These experiments identify a series genes and pathways altered by ATM and 53BP1 mutations and demonstrate that 53BP1 phosphorylation controls its position on chromatin. While the study of ATM / 53BP1 during cortical development is certainly an important topic, the manuscript falls short of providing novel conceptual advance. The organoid defects are certainly clear but could arise from any type of defect, including organoid specification defects. The multi-omics analysis is impressive, but we are left with large numbers of altered genes and pathways, a lot of them probably as consequences of organoid defects, and none of this information is used to identify how ATM / 53BP1 may affect early neurogenesis.

We appreciate the suggestions to improve this manuscript on many levels. Indeed, some mechanistic details remain unclear about the noncanonical role of DNA damage signaling in controlling neurodevelopmental genetic programs. We respectfully hope that the improved understanding of the direct targets of 53BP1, the effect of ATM on 53BP1 binding to chromatin, and the identification of RNF168’s impact on 53BP1 binding to chromatin and gene expression improve this revised manuscript.

Specific comments

1/ The authors suggest that the role of ATM / P-53BP1 during neurodevelopment could be independent of DNA damage, based on absence of increase of gamma-H2AX in NPCs, as compared to ES cells. However, for this suggestion to hold, DNA damage should be looked at in cerebral organoids where the phenotypic analysis was performed. Related to this, do the authors detect increased apoptosis in their organoid models (as suggested by their omics data).

We agree and have examined gamma-H2AX and cleaved caspase 3 (CC3) in WT, *ATM*-KO, 53BP1-S25A, and 53BP1-S25D by immunofluorescence and fluorescence activated cell sorting (FACS). At D35 cortical organoids, gamma-H2AX foci did not differ amongst the 4 groups. Also in response to the feedback from Reviewer 2, we examined CC3 in cortical organoids at D21 and D28. We observed very modest CC3 changes that were inconsistent amongst D21 and D28 WT, *ATM*-KO, 53BP1-S25A, and 53BP1-S25D cortical organoids. Therefore, we concluded that *ATM*-KO, 53BP1-S25A, and 53BP1-S25D did not lead to consistent changes in DNA break or cell death. We added in line 100-106, “We quantitatively compared NPC proliferation, neuronal differentiation, cell death, and cell organization in cortical organoids. Examination of endogenous DNA damage, by γH2AX immunofluorescence, did not reveal marked difference (Supplementary Fig 3A), confirming our WB results in Supplementary Fig 1I. Although quantification of cell death marker cleaved-caspase 3 by FACS revealed a modest increase of cell death in D21 ATM-KO cortical organoids, FACS and immunofluorescence quantification showed that D28 and D35 *ATM*-KO and WT cortical organoids are similar in cell death frequencies (Supplementary Fig 3B-E).

To line 230, “Examination of endogenous DNA damage and cell death, assessed by γH2AX and cleaved-caspase 3, respectively, did not reveal significant differences between 53BP1-S25A, S25D and WT (Supplementary Fig 9).”

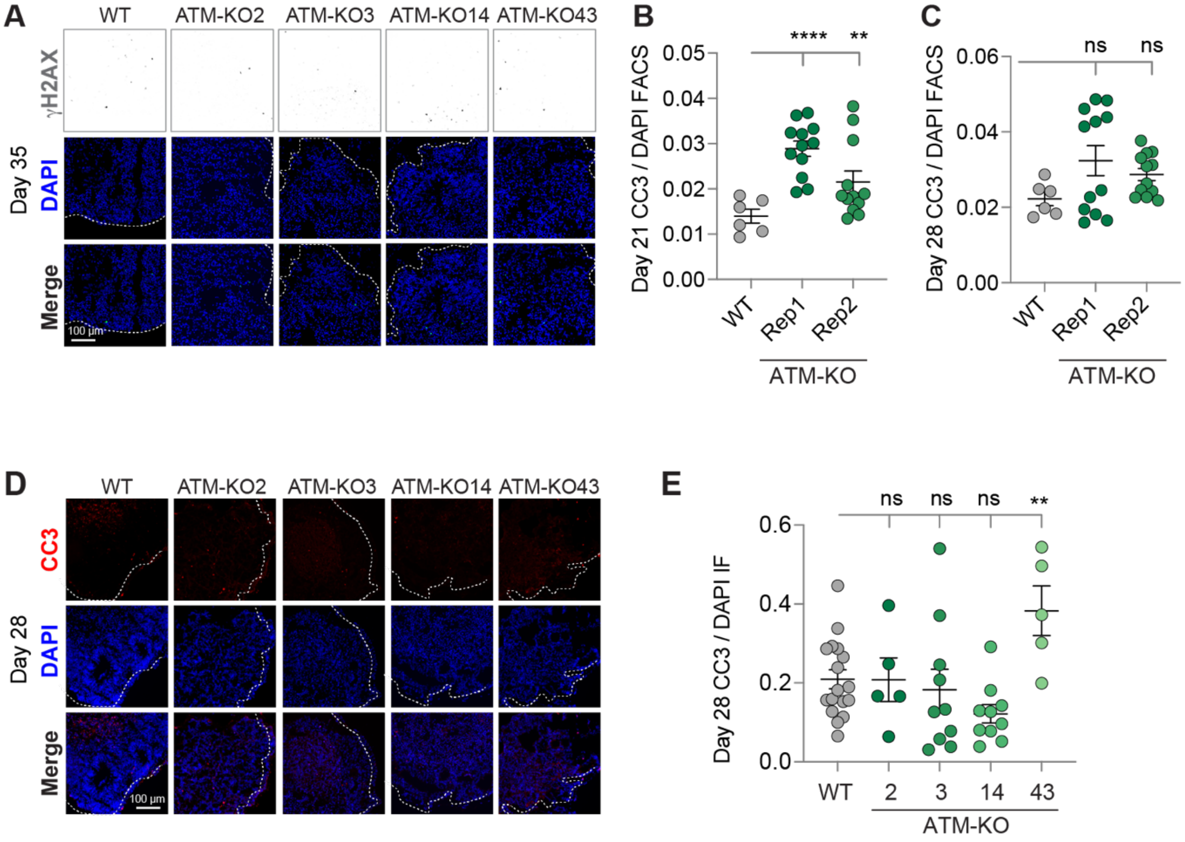

Supplementary Figure 3. Analysis of gH2AX and cleaved-caspase 3 (CC3) in cortical organoids.
(A) Immunofluorescence showed D35 *ATM*-KO and WT cortical organoids had similar γH2AX foci. Bar, 100 µm.
FACS analysis of CC3 in (B) D21 and (C) D28 cortical organoids. Two biological replicates were done, and each data point was based on 3 technical replicate analyses of 10-12-cortical organoids.
(D, E) Immunofluorescence and quantification of CC3 in D28 cortical organoids. Bar, 100 µm.
Graphs are presented in ratios (out of 1), with **, $p<0.01$; ****, $p<0.0001$; ns, not significant by Two-way ANOVA test.

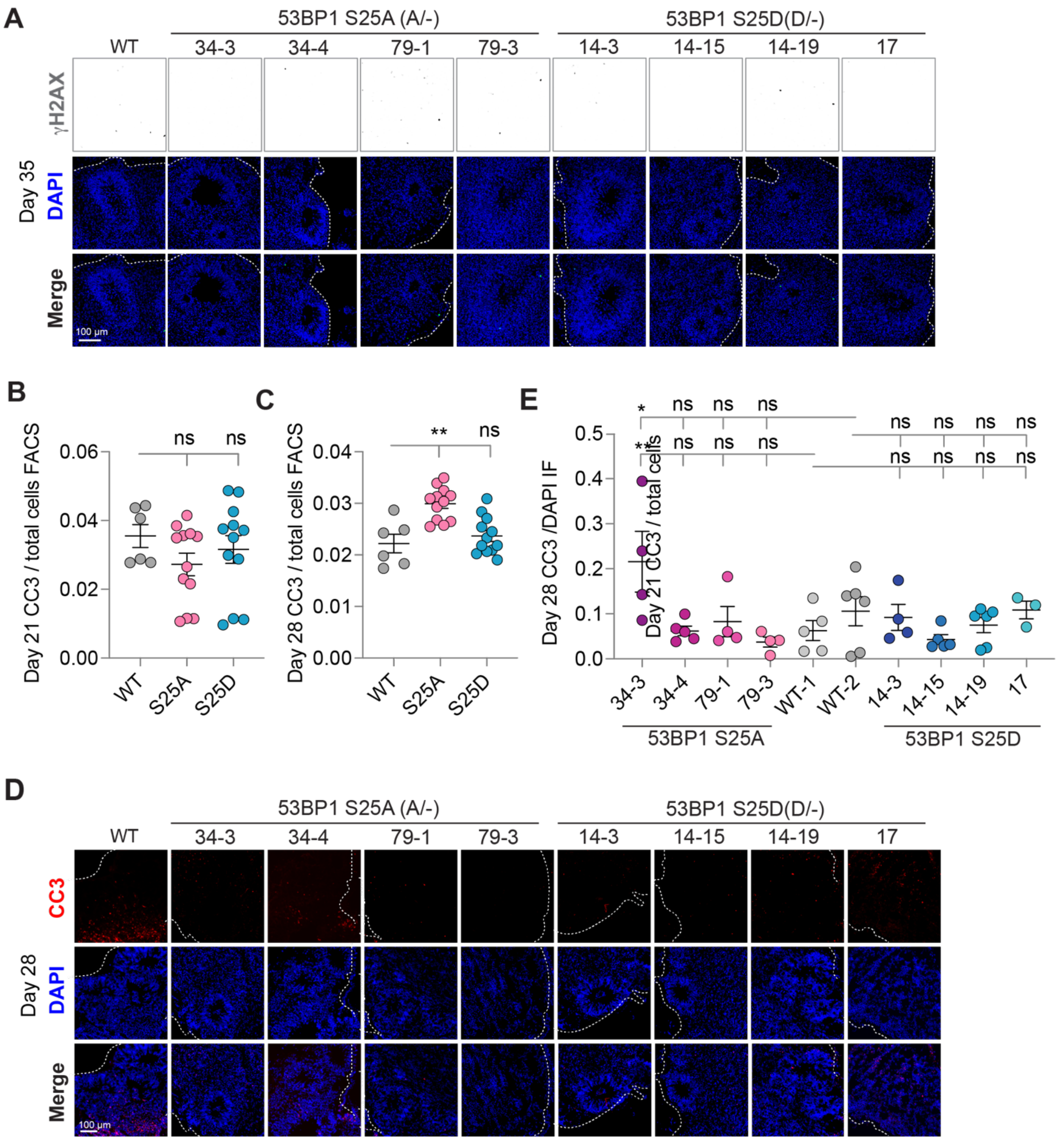

Supplementary Figure 9. Analysis of γH2AX and cleaved-caspase 3 (CC3) in cortical organoids. Immunofluorescence of (A) γH2AX in D35 cortical organoids and (D) cleaved caspase 3 (CC3) in D28 cortical organoids. Bar, 100 µm.

CC3 quantification by FACS of (B) D21 and (C) D28 cortical organoids. For each datapoint, 10-12 organoids from each line were analyzed via 3 technical replicates, and data from 4 mutant lines were consolidated to achieve rigorous comparisons. **, p<0.01 and ns, not significant by Two-way ANOVA test.

2/ The fact that mutated organoids are altered is very clear and convincing, but it is hard to grasp what is causing the defect. The organoids are highly disorganized without clear luminal structures. Is organoid specification correct? In figure 3D for example, PAX6 staining appears severely reduced, which could reflect incorrect identity of the organoids.

This is an important point.

Using RNA-seq datasets of D35 cortical organoids, we examined the expression of markers of forebrain, midbrain, and hindbrain. Supplementary Table 2 lists the TPM (transcripts per million) values of each marker and shows that the mutant organoids specified to forebrain as well as the WT. Compared to forebrain markers, midbrain and hindbrain markers had much lower expression. In line 125-127 of the text, we added "The expression of forebrain markers was similar between WT and *ATM*-KO (and low expression of midbrain and hindbrain markers; Supplementary Table 2), suggesting that the *ATM*-KO cortical organoids specified to the forebrain lineage."

3/ In figures 1 and 3, the quantification of the defects should be properly normalized. The fraction of PH3 or Ki67 cells should be quantified out of PAX6+ progenitors, in order to conclude whether progenitor proliferation is affected. For cell fate, it would be good to quantify the fraction of PAX6+ and Ctip2+ cells out of total cells (DAPI) or to present a PAX6/Ctip2 ratio.

This suggestion has indeed helped us understand the mutants' phenotypes better.

For *ATM*-KO, we added to line 100-120, "We quantitatively compared NPC proliferation, neuronal differentiation, cell death, and cell organization in *ATM*-KO versus WT cortical organoids. Examination of endogenous DNA damage, by γH2AX immunofluorescence, did not reveal marked difference (Supplementary Fig 3A), confirming our WB results in Supplementary Fig 1I. Although quantification of cell death marker cleaved-caspase 3 by FACS revealed a modest increase of cell death in D21 ATM-KO cortical organoids, FACS and immunofluorescence quantification showed that D28 and D35 *ATM*-KO and WT cortical organoids are similar in cell death frequencies (Supplementary Fig 3B-E). Cell proliferation frequencies did not significantly differ between D28 and D35 *ATM*-KO and WT cortical organoids (Supplementary Fig 4). We next quantified immature neuronal marker NEUN and PAX6/CTIP2 ratios and revealed that despite lower levels of immature neuronal differentiation, ATM-KO exhibited higher neuronal maturation (Fig 2A-C, Supplementary Fig 5A). These data suggest that *ATM*-KO fastens the phase of immature neuronal differentiation, leading to enhanced neuronal maturation. Finally, we quantified ZO-1-positive ventricular surfaces and the organization of PH3-positive and PAX6-positive cells around ventricular surfaces. *ATM*-KO ventricular surfaces are similar to WT at D28 (Supplementary Fig 5B-D) but their numbers are much reduced by D37 (Fig 2D). NPC organization around the ventricular surfaces were similarly organized in D37 (Fig 2D, Supplementary 5E); however, fewer *ATM*-KO proliferative cells were adjacent to ventricular surfaces (Fig 2E). These data suggest that *ATM*-KO enhances neuronal maturation and cellular disorganization in developing cortical organoids. By D55, *ATM*-KO cortical organoids had similar size distribution as the control (Supplementary Fig 6A-B). Thus, ATM controls neuronal differentiation and cellular organization to form ventricular zone-like regions in cortical organoids."

For 53BP1-S25A and S25D, we added to lines 230-241, "Examination of endogenous DNA damage and cell death, assessed by γH2AX and cleaved-caspase 3, respectively, did not reveal significant differences between 53BP1-S25A, S25D and WT (Supplementary Fig 9). To explore the developmental timing of the cellular phenotypes, we quantified KI67, NPC marker PAX6, and neuronal marker CTIP2 in D14, D21, D28, and D35 cortical organoids. At D28, 53BP1-S25A and S25D cortical organoids had significantly lower cell proliferation and higher neuronal differentiation (Fig 5).

Quantification of the tight junction protein ZO-1 showed significantly fewer ZO-1-positive ventricular surfaces in D28 53BP1-S25A and S25D cortical organoids compared to WT (Supplementary Fig 10). For the ventricles that did form in D28 53BP1-S25A and S25D, their surface areas did not significantly differ from those of WT (Supplementary Fig 10B). These data suggest that lower ventricles formation, lower cell proliferation, and higher neuronal differentiation contributed to the depletion of progenitor pools and smaller cortical organoids in 53BP1-S25A and S25D."

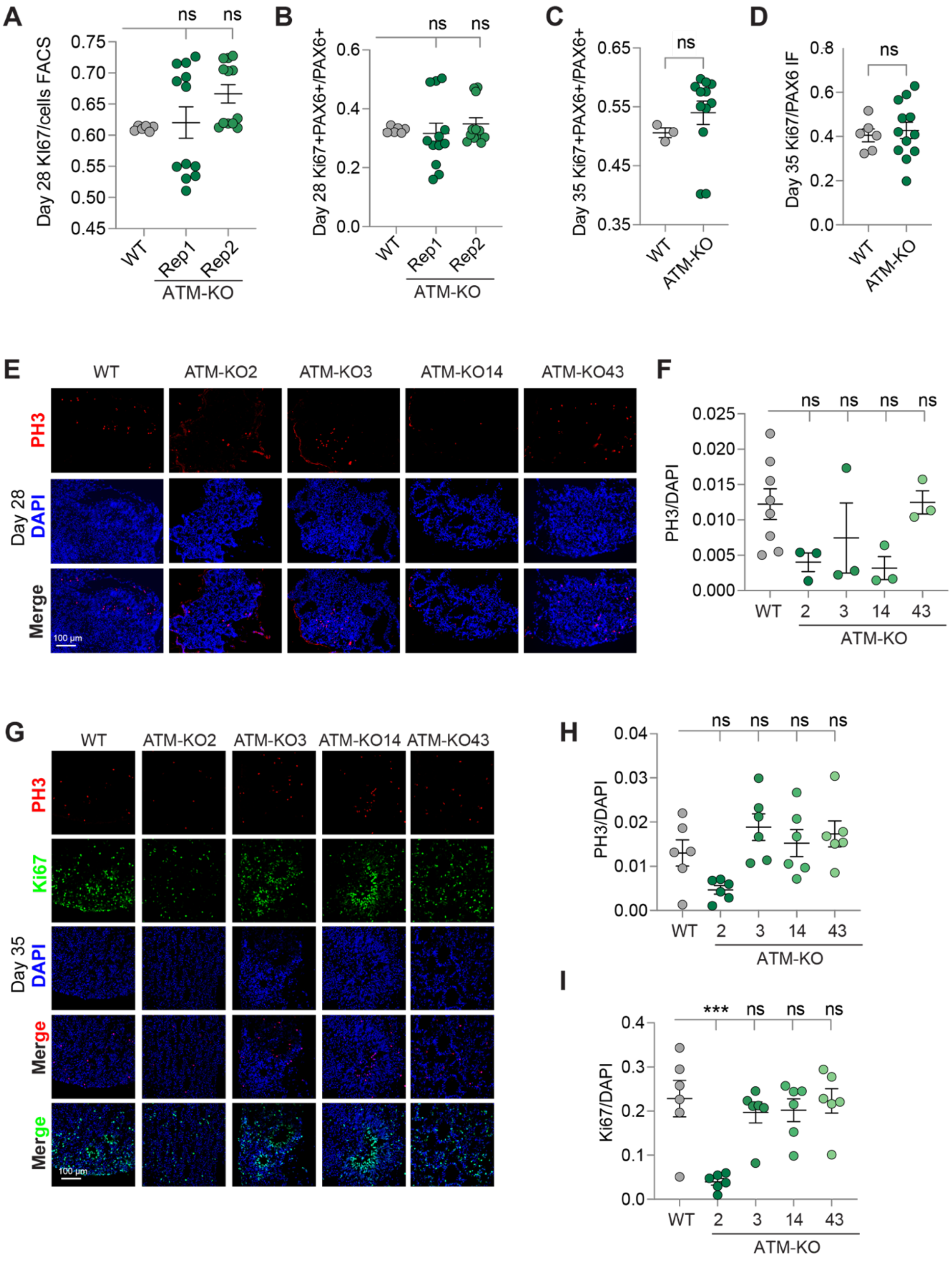

Supplementary Figure 4. Analysis of cell proliferation in cortical organoids.
FACS analysis of PAX6 and KI67 in (A, B) D28 and (C) D35 cortical organoids. Each data point was based on the 3 technical replicate analyses of 10 cortical organoids.
(D) Quantification of KI67/PAX6 ratios in immunofluorescence of D35 cortical organoids. Each data point represents quantification of cells in 1 cortical organoid.
(E, F) Immunofluorescence and quantification of H3-pS10 (PH3) in D28 cortical organoids. Bar, 100 μm.
(F-H) Immunofluorescence and quantification of PH3 and KI67 in D35 cortical organoids. Bar, 100 μm.
***, p<0.001; ns, not significant by Two-way ANOVA test.

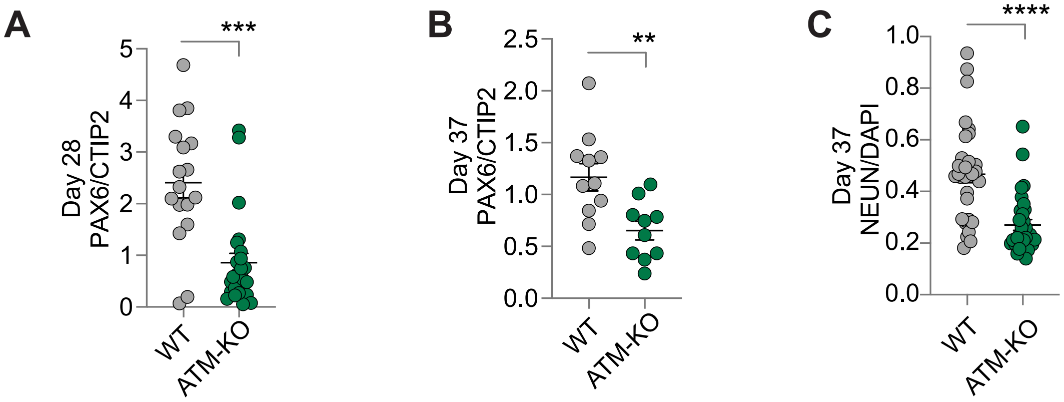

D

Day 37

Line	#ZO1 rings	% organized PAX6
WT	27	77.78
ATM-KO	7	85.71

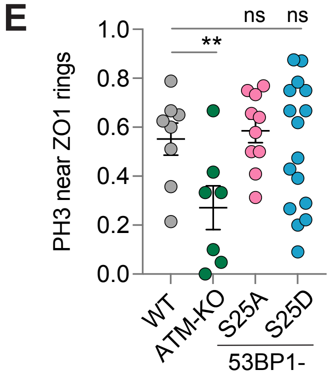

Figure 2. Analysis of neuronal differentiation and cell organization in cortical organoids.
Quantification of PAX6/CTIP2 ratios in (A) D28 and (B) D37 cortical organoids.
(C) Quantification of NEUN/DAPI in D37 cortical organoids.
(D) In D37 cortical organoids, 6 organoids were surveyed to count ZO-1-positive apical surfaces and proportions of PAX6-positive NPCs that are organized around the apical surfaces.
(E) Proportions of PH3-positive cells that are adjacent to ZO-1-positive apical surfaces (“rings”). Data from 53BP1-S25A and S25D were included for comparison.
**, p<0.01; ***, p<0.001; ****, p<0.0001; ns, not significant by Welch’s t-test in (A-C) and Two-way ANOVA test in (D).

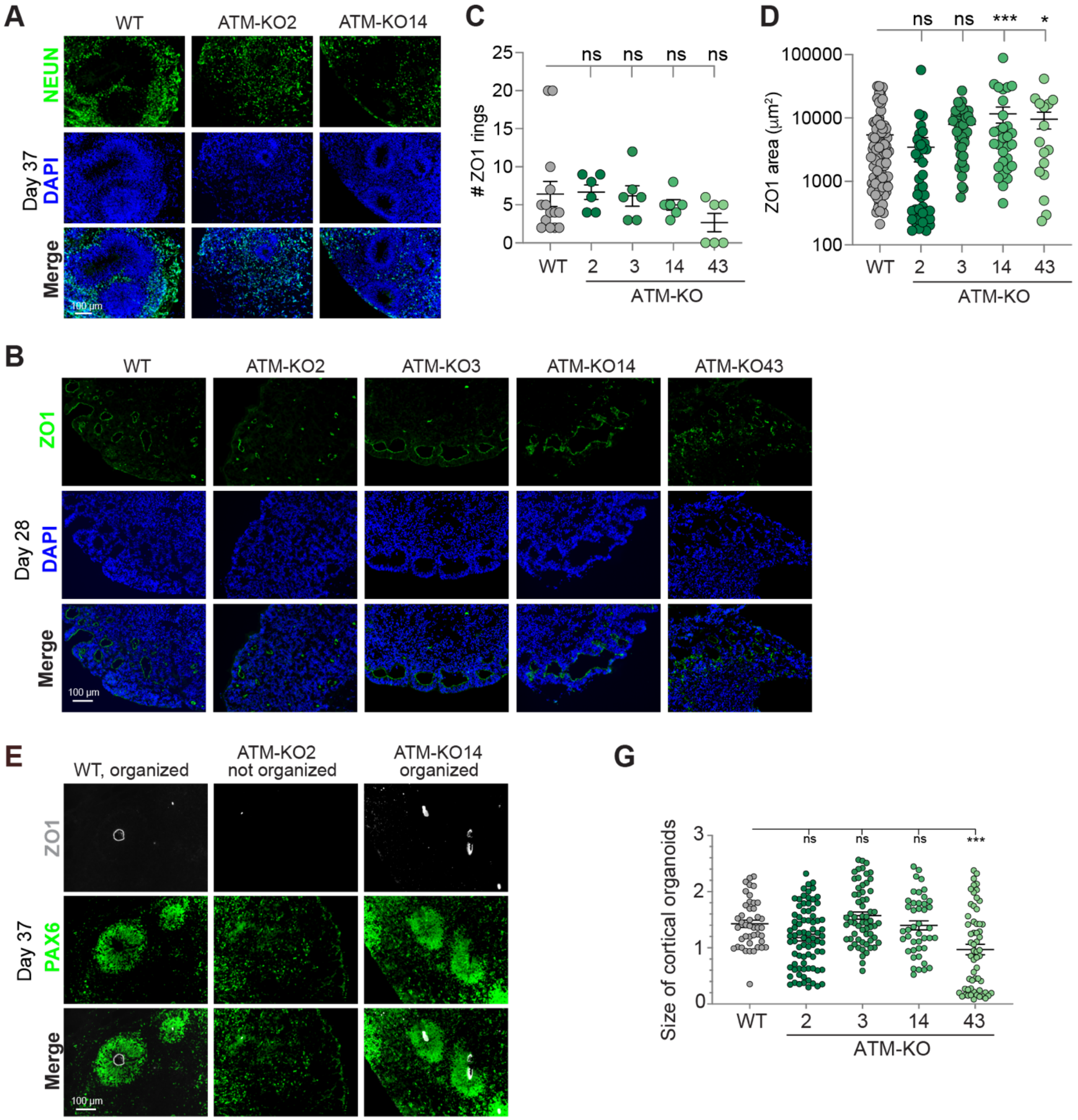

Supplementary Figure 5. Immunofluorescence analyses of cortical organoids and NPCs.
Immunofluorescence of (A) NEUN and (E) ZO-1 and PAX6 in D37 cortical organoids. Bar, 100 µm.
(B) Immunofluorescence of ZO-1 in D28 cortical organoids. Bar, 100 µm.
Quantification of the (C) number and (D) surface area of ZO-1-positive ventricles in D28 cortical organoids. *, p<0.05; ***, p<0.001; ns, not significant by Two-way ANOVA test.
(F) Bright-field images of cortical organoids formed by *ATM*-KO2, 3, 14, 43, and WT control at day 55 of differentiation. Bar, 1.5 mm.
(G) The size of cortical organoids was compared between groups by one-way ANOVA with Dunnett's multiple comparisons test, with ns, not significant and ***, *p*<0.001. n = 13 organoids/group.

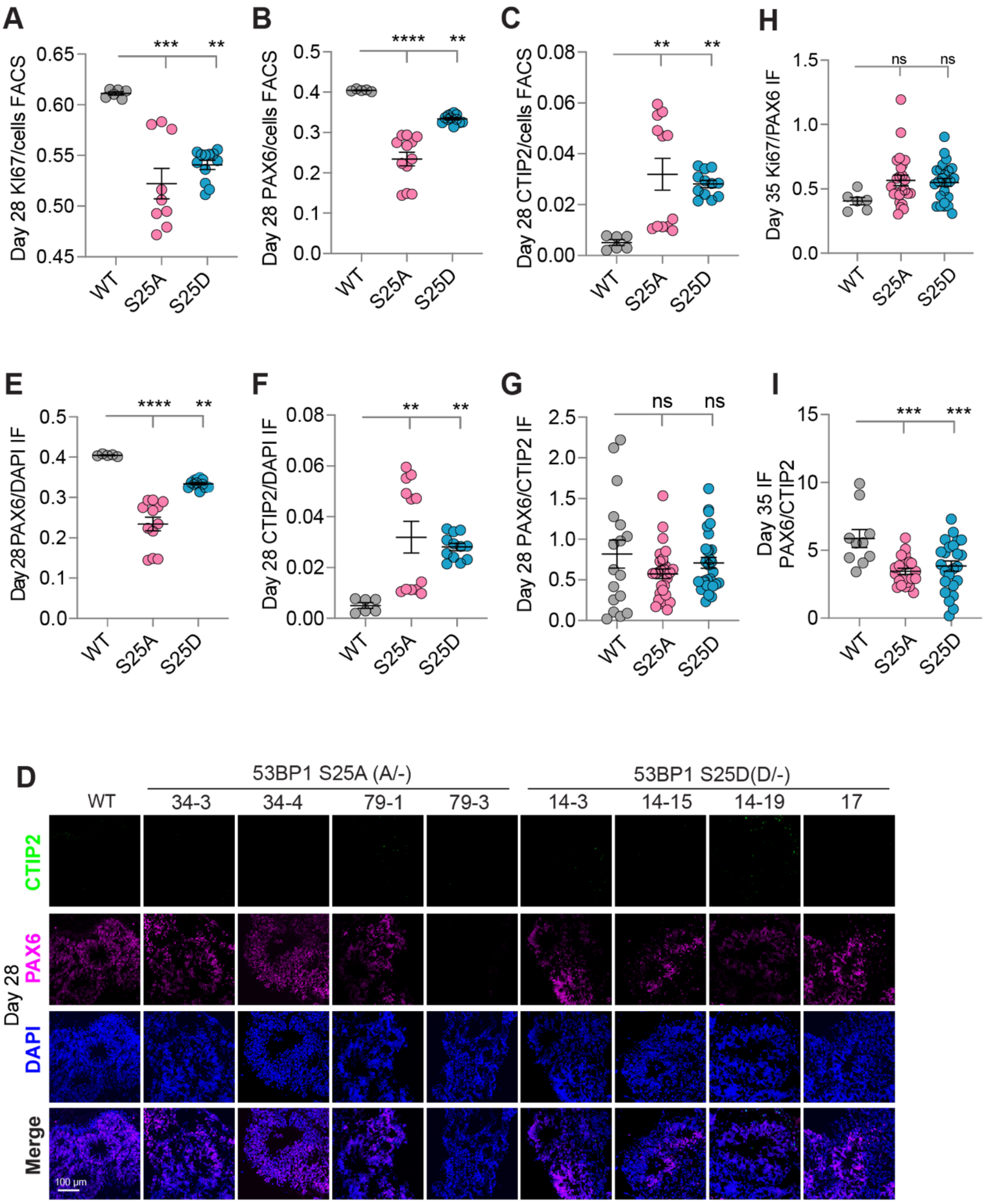

Figure 5. 53BP1-S25A and S25D lower cell proliferation in cortical organoids.

FACS quantified ratios of (A) KI67, (B) PAX6, and (C) CTIP2 to total cells in D28 cortical organoids. (D) Immunofluorescence of PAX6 and CTIP2 in D28 cortical organoids. Bar, 100 μm. Quantification of immunofluorescence signals of (E) PAX6/DAPI, (F) CTIP2/DAPI, and (G) PAX6/CTIP2 in D28 cortical organoids. Each data point represents quantification of cells in 1 cortical organoid. Quantification of (H) KI67/PAX6 and (I) PAX6/CTIP2 ratios in immunofluorescence of D35 cortical organoids. Each data point represents quantification of cells in 1 cortical organoid.

*, $p<0.05$; **, $p<0.01$; ***, $p<0.001$; ****, $p<0.0001$; ns, not significant by Two-way ANOVA test.

4/ The authors should provide a ZO-1 staining (or similar) to quantify apical surface area and the formation (or absence of formation) of lumen.

This is a very good point.

Also in response to the feedback from Reviewer 2, we first evaluated the formation of ZO-1-positive lumen in WT cortical organoids at days 24, 28, and 35 of differentiation. This showed that WT cortical organoids robustly formed ZO1-1-positive lumen at day 28 of differentiation. Next, we quantified ZO-1-positive lumen numbers and apical surface areas in D28 WT and mutant cortical organoids.

For *ATM*-KO, we added to line 111-117, "Finally, we quantified ZO-1-positive ventricular surfaces and the organization of PH3-positive and PAX6-positive cells around ventricular surfaces. *ATM*-KO ventricular surfaces are similar to WT at D28 (Supplementary Fig 5B-D) but their numbers are much reduced by D37 (Fig 2D). NPC organization around the ventricular surfaces were similarly organized in D37 (Fig 2D, Supplementary 5E); however, fewer *ATM*-KO proliferative cells were adjacent to ventricular surfaces (Fig 2E). These data suggest that ATM-KO enhances neuronal maturation and cellular disorganization in developing cortical organoids."

For 53BP1-S25A and S25D, we added to line 235-241, "Quantification of the tight junction protein ZO-1 showed significantly fewer ZO-1-positive ventricular surfaces in D28 53BP1-S25A and S25D cortical organoids compared to WT (Supplementary Fig 10). For the ventricles that did form in D28 53BP1-S25A and S25D, their surface areas did not significantly differ from those of WT (Supplementary Fig 10B). These data suggest that lower ventricle formation, lower cell proliferation, and higher neuronal differentiation contributed to the depletion of progenitor pools and smaller cortical organoids in 53BP1-S25A and S25D."

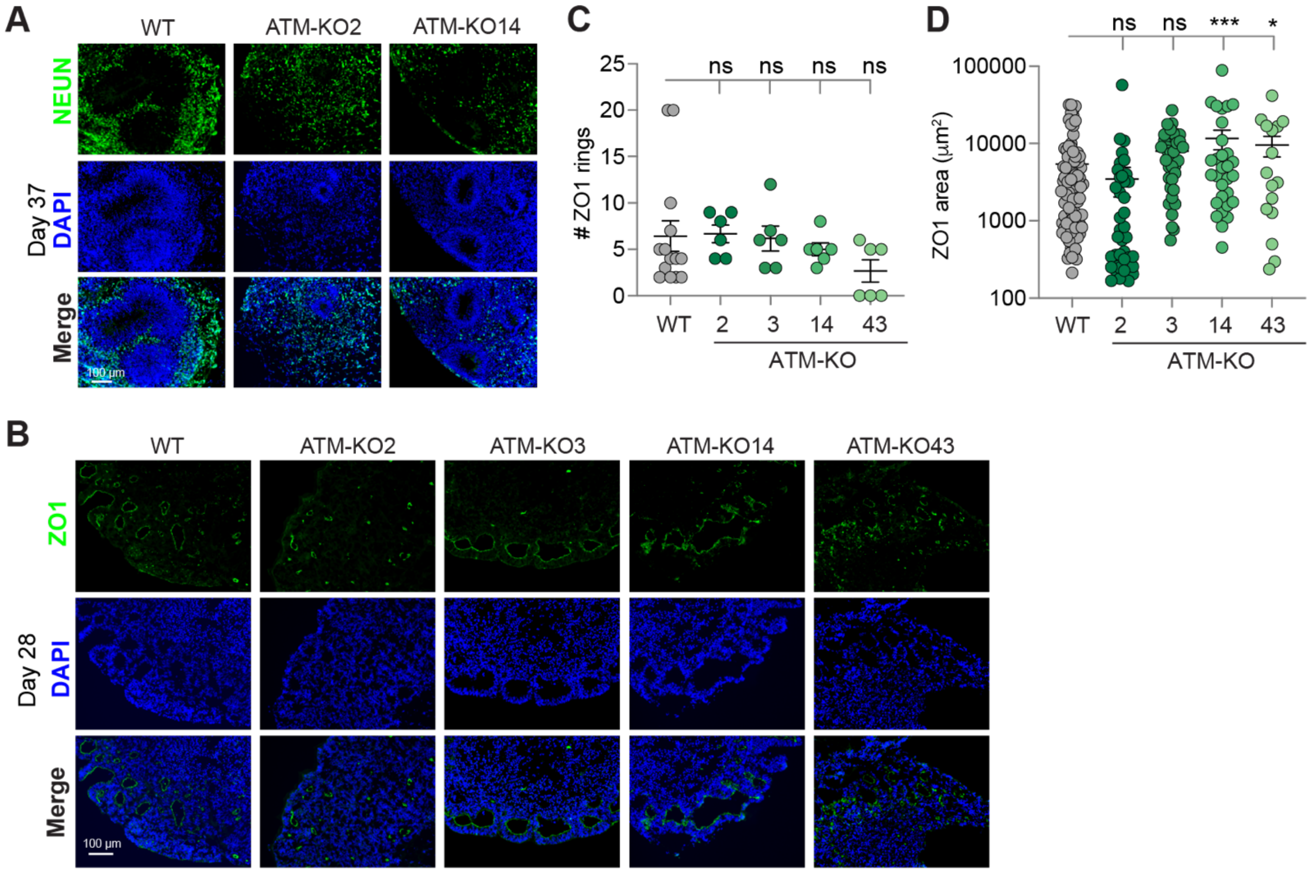

Supplementary Figure 5. Immunofluorescence analyses of cortical organoids and NPCs.
(A) Immunofluorescence of NEUN in D37 cortical organoids. Bar, 100 µm.

(B) Immunofluorescence of ZO-1 in D28 cortical organoids. Bar, 100 µm.
Quantification of the (C) number and (D) surface area of ZO-1-positive ventricles in D28 cortical organoids. *, $p<0.05$; ***, $p<0.001$; ns, not significant by Two-way ANOVA test.

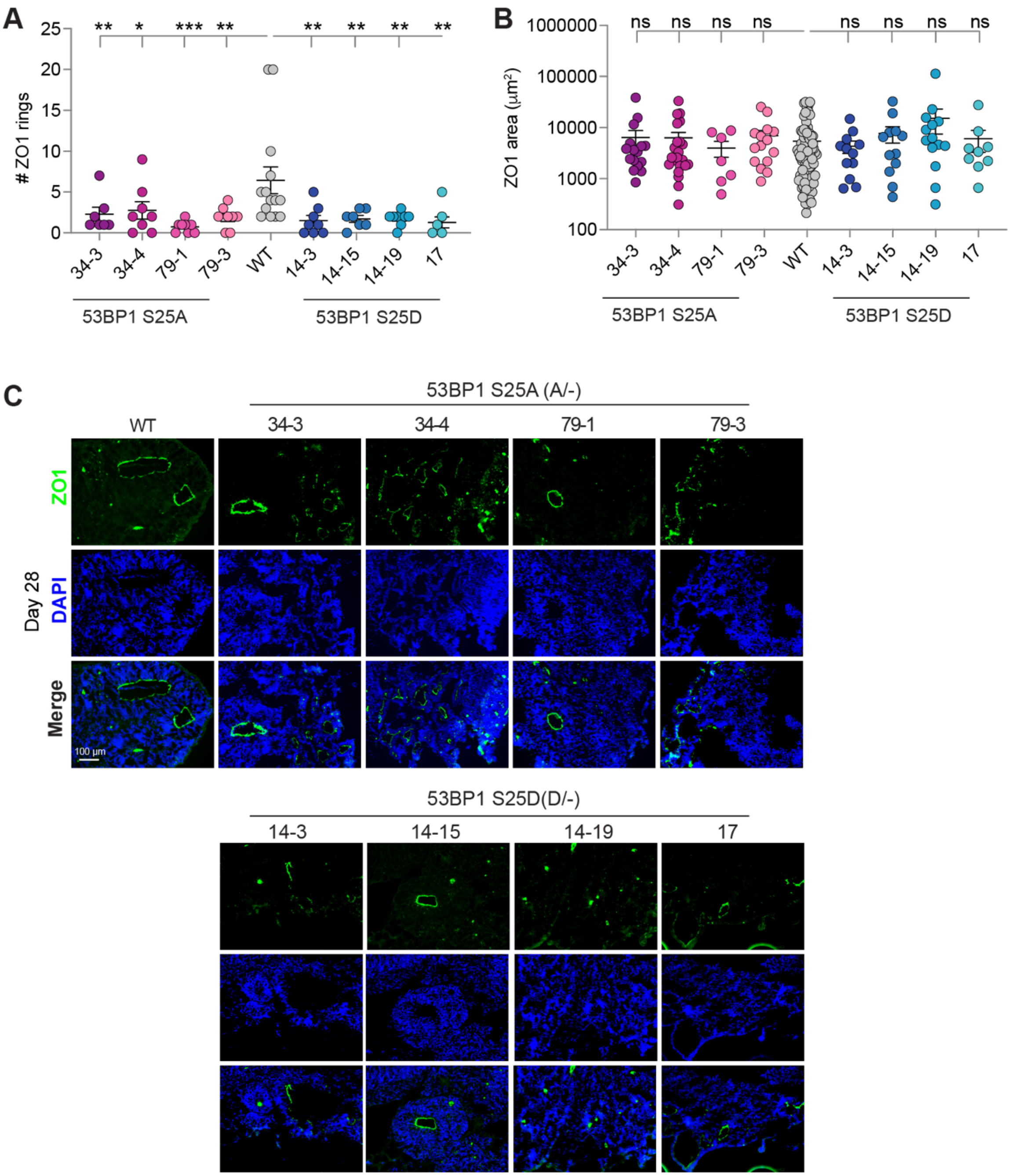

Supplementary Figure 10. 53BP1-pS25 promotes ventricle formation in cortical organoids.
Quantification of the (A) number and (B) surface area of ZO-1-positive ventricles.
(C) Immunofluorescence of ZO-1 in D28 cortical organoids. Bar, 100 µm.
*, $p<0.05$; **, $p<0.01$; ***, $p<0.001$; ns, not significant by Two-way ANOVA test.

5/ The PAX6 staining appears very different between ATM KO and 53BP1 KI conditions. Does this reflect different molecular alterations?

This is indeed important. New RNA-seq analyses showed that ATM-KO, 53BP1-S25A, and 53BP1-S25D NPCs shared similar disruptions to genetic programs (Fig 2F-G and Supplementary Fig 12) Additional analyses showed that fastened neuronal maturation and cellular disorganization in *ATM*-KO, 53BP1-S25A, and 53BP1-S25D are more similar than we originally reported.

Additional, separate analyses of the RNA-seq datasets from D35 cortical organoids of WT, ATM-KO, 53BP1-S25A, and 53BP1-S25D (Fig 4G, Supplementary 7E-G). We added in "Results," pg 299-301, "GSEA showed a significant enrichment of concordantly differentially expressed genes amongst ATM-KO vs. WT, 53BP1-S25A, and 53BP1-S25D (Fig 6G, Supplementary 13A-C)."

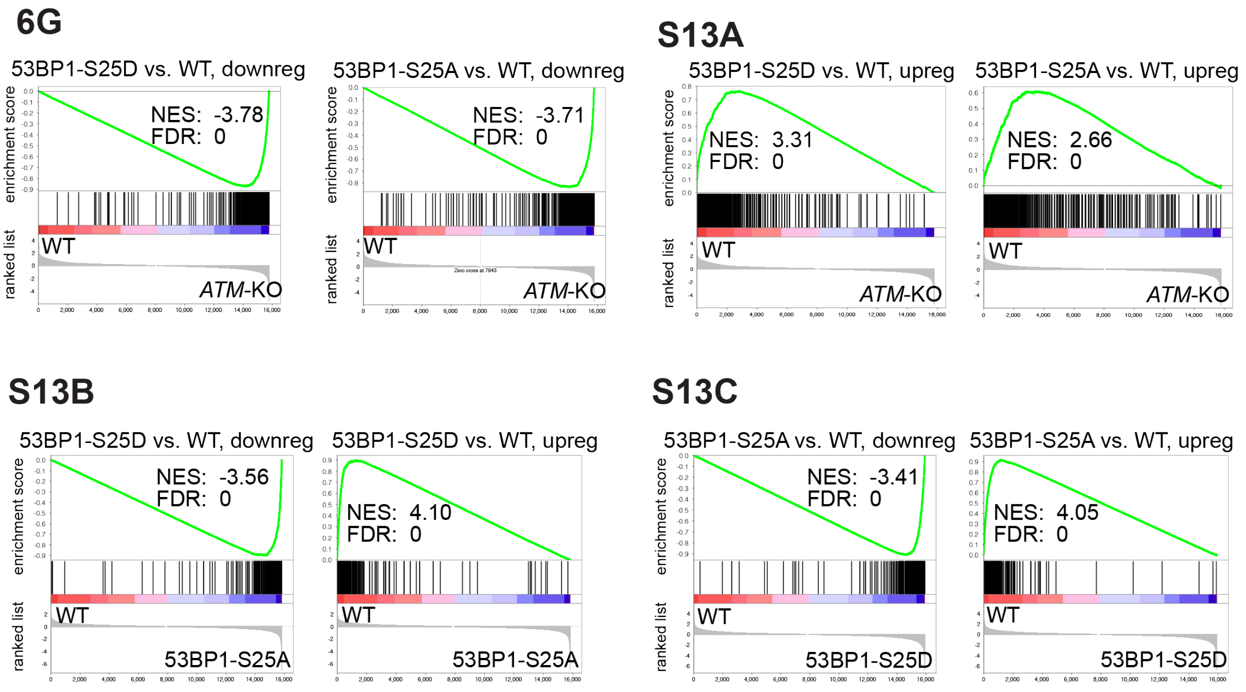

GSEA showed that differentially expressed genes in ATM-KO vs. WT were significantly and concordantly enriched in those found in (Fig 6G) 53BP1-S25D vs. WT and (Supp Fig 13A) 53BP1-S25A vs. WT. (Supp Fig 13B, C) Similar GSEA results were obtained when analyzing differentially expressed genes in 53BP1-S25A vs. WT and 53BP1-S25D vs. WT. *P* values were calculated by the hypergeometric test, assuming normal data distribution.
