# Supplementary material for: Phosphorylation of the DNA damage repair factor 53BP1 by ATM kinase controls neurodevelopmental programs in cortical brain organoids
Source: PLoS Biol. 2024 Sep 3;22(9):e3002760. doi: 10.1371/journal.pbio.3002760 (PMC11398655; doi:10.1371/journal.pbio.3002760)
Supplement: Attachment — Submitted filename: response.pdf [file pbio.3002760.s029.pdf]

We appreciate the reviewers' their thoughtful comments, which have significantly improved all sections of this manuscript. We agree with Reviewer 1 and the editorial office's decision to reduce our discussion about the influence of HH signaling over ATM kinase.

Reviewer #1: The manuscript remains descriptive, since the authors are still unable to explain how the ATM-gH2AX-RNF168-53BP1 is activated, and how/why this non-canonical role of DNA damage signaling is controlling specific genetic programs in NPCs and cortical organoids, while in other somatic tissues the gH2AX-RNF169-53BP1 pathway is a well-established to specifically regulate DNA repair. They have tried to address the activating stimulus of ATM, and in new experiments with cyclopamine (HH inhibitor) they find reduced pS25-53BP1 levels and suggest that in cortical neurons, hedgehog signalling is likely required to activate ATM. Again, HH would be an unexpected and non-canonical activator of ATM, and the claimed alterations of pS25-53BP1 levels in new immunoblot data in new Supp Fig 16 aren't clear-cut enough to convince me that these conclusions have sufficiently substantiated evidence basis. The author rebuttal states that "the exact clarification of mechanisms promoting ATM activities, especially in directing its kinase activity at specific promoters, is beyond the scope of this study. " yet nevertheless they also state that this study "has broad implications about diseases associated with ATM, including ataxia telangiectasia". Although, I acknowledge that the authors have attempted to address my concerns with new experiments to explore the role of gH2AX, and add new correlative data regarding RNF168, there absence of solid evidence to explain the mechanism basis of this non-canonical role of the ATM-gH2AX-53BP1 axis, and so I remain cautiously sceptical of the proposed mechanisms and their overall impact.

We understand Reviewer 1's rationale and agree with their assessment about HH signaling. We made multiple text changes to reduce statements about the effect of HH signaling on ATM. Line 387 was changed to "HH signaling pathway may affect pS25-53BP1 levels during neural differentiation." Line 419 was changed to "HH, TGFβ, and WNT signaling pathways likely affect ATM during neural induction." And line 425 was changed to "These molecular pathways may function in diseases associated with ATM,"

Reviewer #2: The authors addressed all my comments and improved the manuscript substantially. I can recommend the current version of the manuscript for publication in PLOS Biology.

We thank the Reviewer for the encouraging response and guidance to improve the manuscript.

Reviewer #3: The authors have now addressed all of my previous concerns: the absence of DNA damage increase with differentiation, the correct specification of mutant organoids (comments 1 and 5), the normalization method for the quantifications, and the use of ZO-1 staining to quantify the lumen defects.

We thank the Reviewer for the encouraging response and guidance to improve the manuscript.
